# A measles and rubella vaccine microneedle patch in The Gambia: a phase 1/2, double-blind, double-dummy, randomised, active-controlled, age de-escalation trial

**DOI:** 10.1016/S0140-6736(24)00532-4

**Published:** 2024-05-11

**Authors:** Ikechukwu Adigweme, Mohammed Yisa, Michael Ooko, Edem Akpalu, Andrew Bruce, Simon Donkor, Lamin B Jarju, Baba Danso, Anthony Mendy, David Jeffries, Anne Segonds-Pichon, Abdoulie Njie, Stephen Crooke, Elina El-Badry, Hilary Johnstone, Michael Royals, James L Goodson, Mark R Prausnitz, Devin V McAllister, Paul A Rota, Sebastien Henry, Ed Clarke

**Affiliations:** a Vaccines and Immunity Theme, Medical Research Council Unit The Gambia at the London School of Hygiene & Tropical Medicine, Banjul, The Gambia; b HJ-Clinical Trial Consultancy, George, South Africa; c Micron Biomedical, Atlanta, GA, USA; d Global Immunization Division, Global Health Center, Centers for Disease Control and Prevention, Atlanta, GA, USA; e Division of Viral Diseases, National Center for Immunization and Respiratory Diseases, Centers for Disease Control and Prevention, Atlanta, GA, USA

## Abstract

**Background:**

Microneedle patches (MNPs) have been ranked as the highest global priority innovation for overcoming immunisation barriers in low-income and middle-income countries. This trial aimed to provide the first data on the tolerability, safety, and immunogenicity of a measles and rubella vaccine (MRV)-MNP in children.

**Methods:**

This single-centre, phase 1/2, double-blind, double-dummy, randomised, active-controlled, age de-escalation trial was conducted in The Gambia. To be eligible, all participants had to be healthy according to prespecified criteria, aged 18–40 years for the adult cohort, 15–18 months for toddlers, or 9–10 months for infants, and to be available for visits throughout the follow-up period. The three age cohorts were randomly assigned in a 2:1 ratio (adults) or 1:1 ratio (toddlers and infants) to receive either an MRV-MNP (Micron Biomedical, Atlanta, GA, USA) and a placebo (0·9% sodium chloride) subcutaneous injection, or a placebo-MNP and an MRV subcutaneous injection (MRV-SC; Serum Institute of India, Pune, India). Unmasked staff ransomly assigned the participants using an online application, and they prepared visually identical preparations of the MRV-MNP or placebo-MNP and MRV-SC or placebo-SC, but were not involved in collecting endpoint data. Staff administering the study interventions, participants, parents, and study staff assessing trial endpoints were masked to treatment allocation. The safety population consists of all vaccinated participants, and analysis was conducted according to route of MRV administration, irrespective of subsequent protocol deviations. The immunogenicity population consisted of all vaccinated participants who had a baseline and day 42 visit result available, and who had no protocol deviations considered to substantially affect the immunogenicity endpoints. Solicited local and systemic adverse events were collected for 14 days following vaccination. Unsolicited adverse events were collected to day 180. Age de-escalation between cohorts was based on the review of the safety data to day 14 by an independent data monitoring committee. Serum neutralising antibodies to measles and rubella were measured at baseline, day 42, and day 180. Analysis was descriptive and included safety events, seroprotection and seroconversion rates, and geometric mean antibody concentrations. The trial was registered with the Pan African Clinical Trials Registry PACTR202008836432905, and is complete.

**Findings:**

Recruitment took place between May 18, 2021, and May 27, 2022. 45 adults, 120 toddlers, and 120 infants were randomly allocated and vaccinated. There were no safety concerns in the first 14 days following vaccination in either adults or toddlers, and age de-escalation proceeded accordingly. In infants, 93% (52/56; 95% CI 83·0–97·2) seroconverted to measles and 100% (58/58; 93·8–100) seroconverted to rubella following MRV-MNP administration, while 90% (52/58; 79·2–95·2) and 100% (59/59; 93·9–100) seroconverted to measles and rubella respectively, following MRV-SC. Induration at the MRV-MNP application site was the most frequent local reaction occurring in 46 (77%) of 60 toddlers and 39 (65%) of 60 infants. Related unsolicited adverse events, most commonly discolouration at the application site, were reported in 35 (58%) of 60 toddlers and 57 (95%) of 60 infants that had received the MRV-MNP. All local reactions were mild. There were no related severe or serious adverse events.

**Interpretation:**

The safety and immunogenicity data support the accelerated development of the MRV-MNP.

**Funding:**

Bill & Melinda Gates Foundation.

## Introduction

Substantial progress in reducing the global burden of measles and rubella infections has been made through vaccination. Between 2000 and 2021 the annual number of measles deaths has fallen by around 83%, from 761 000 to 128 000,^1^ and an estimated 56 million measles deaths have been averted in this period.^1^ Similarly, in 2020, the annual number of rubella cases fell by 96%, from 670 894 to 10 194.^2^

All six WHO regions have committed to measles elimination.^3^ To achieve this, WHO recommends that countries consistently achieve at least 95% two-dose measles vaccine coverage across all districts.^4^ In countries with ongoing measles transmission, the first dose is recommended in children aged 9 months, and the second dose at age 15–18 months.^4^ Through the use of the combined measles and rubella vaccine (MRV), these targets also support rubella elimination, for which around 80% population immunity is required to prevent transmission.^5^

**Research in context**

**Evidence before this study**

We searched PubMed to identify articles published from database inception to July 1, 2023, using the following search terms with appropriate Boolean operators: “microneedle patch”, “microarray patch”, “measles”, “rubella”, “vaccin*”, “immun*”, “safety”, “clinical trial”, and “systematic review”. We additionally identified relevant publications in the grey literature through search engines and links with key organisations working in the field. The Vaccine Innovation Prioritization Strategy, developed through a partnership between WHO, UNICEF, Gavi, the Vaccine Alliance, PATH, and the Bill & Melinda Gates Foundation, has recently defined microneedle patch (MNP) development as the number one global priority for overcoming barriers to vaccination in low-income and middle-income countries (LMICs) and for attaining coverage targets and vaccine equity. The technology is considered to be key to achieving measles and rubella elimination, for which all WHO regions now have goals that have been consistently missed due to the exceptionally high coverage required to interrupt transmission. To this end, a Target Product Profile has been published by WHO and UNICEF that defines what are seen as the key attributes for measles and rubella MNPs if elimination goals are to be met. Five studies have been conducted in adults examining the administration of the inactivated influenza vaccine by dissolvable (n=2) or solid (n=3) microneedle patches. The studies confirmed the tolerability and safety of the patches and reported similar immunogenicity to vaccines delivered subcutaneously or intramuscularly, and in some cases included antigen dose sparing. One study has been conducted using dissolvable MNPs to administer a Japanese encephalitis vaccine. Tolerability, safety, and dose sparing was demonstrated. There are no published data on the use of MNPs to deliver vaccines to children or infants (the key target group) or on the use of the technology to administer the measles and rubella vaccine (MRV). Preclinical data on the measles and rubella MNP supported the initiation of this trial.

**Added value of this study**

This trial provides the first data on the use of MNPs to deliver vaccines to children and infants, and the first data on dissolving measles and rubella MNPs. In adults, MRV-primed toddlers aged 15–18 months, and MRV-naive infants aged 9–10 months, the MRV-MNPs were well tolerated and safe. Induration at the application site was common, occurring in nearly half of all toddlers and infants but was mild in all cases and resolved without treatment. None of the local reactions were of any safety concern. Discolouration at the application site, almost exclusively hyperpigmentation, was also common, occurring in nearly 50% of toddlers and over 80% of infants. Over half of these reactions resolved within 42 days, and almost all by day 180. There were no severe or serious adverse events considered to be related to the MNP. The immunogenicity of the MRV when administered by MNP was similar to its immunogenicity when administered subcutaneously by needle and syringe in all three age groups. Based on serum neutralising antibodies, the gold-standard correlates of protection for measles and rubella, 93% of infants who were measles seronegative at baseline seroconverted following the MRV-MNP, compared with 90% of infants who had the vaccine administered subcutaneously. All rubella seronegative infants in both groups seroconverted. Over 90% of infants remained seropositive for measles and 100% of infants remained seropositive for rubella at day 180. Despite high baseline antibody concentrations in the toddlers, reflecting their previous measles and rubella vaccination, increases in the antibody concentrations to both antigens occurred and were similar across the two methods of administration. These are the first data demonstrating directly that MNPs are viable for the delivery of vaccines to children and infants.

**Implications of all the available evidence**

MRV administration by MNP is well tolerated and safe in adults, toddlers, and infants. The immunogenicity of the vaccine delivered by MNP is similar to the immunogenicity of the vaccine when administered subcutaneously by needle and syringe. MNPs are considered to be of highest priority for overcoming barriers to immunisation in LMICs and to achieving measles and rubella elimination. This phase 1/2 trial data supports their accelerated development for this purpose as well as ongoing work to apply the technology for the delivery of other priority vaccines.

Globally, first dose measles vaccine coverage increased from 72% in 2000 to 86% in 2019.^1^ Coverage in the WHO African region increased from 53% to 70% over the same period.^1^ In 2021, second dose measles vaccine coverage was 71% globally, although it was only 41% in the WHO African region.^1^ Furthermore, there is significant heterogeneity both between and within countries.^6^ In 2019, four of the 15 countries in west Africa had a first dose coverage of at least 90%, while the coverage in two countries was below 60%. In Nigeria, coverage ranged from 80% in some regions to less than 20% in others.^6^, ^7^ Thus, despite progress, the coverage achieved through routine immunisations remains considerably below the elimination targets.^8^

Consequently, periodic supplementary immunisation activities (SIA), which target an entire population (typically children aged 9 months to <5 years) for vaccination during an intensive campaign period, continue to be necessary to increase population immunity and as a response to measles outbreaks.^1^, ^9^ Over 150 million children received measles vaccines through SIA in 2021.^1^ Such vertical approaches tend to improve coverage equity compared with routine immunisation services, which vulnerable people have the most difficulty in accessing.^10^, ^11^ However, SIA are personnel intensive, logistically complex, and variable in their capacity to reach those known as zero-dose children, who have received no previous measles vaccines.^11^, ^12^ Thus, alternative strategies to equitably improve routine coverage and to facilitate SIA are essential.

Microneedle patches (MNPs) offer a number of programmatic advantages over needle and syringe-based MRV administration.^13^ Indeed, the Vaccine Innovation Prioritization Strategy, a consortium including WHO, UNICEF, and Gavi, the Vaccine Alliance, recently ranked the development of MNP as the highest global priority for achieving equity of vaccine coverage in low-income and middle-income countries.^14^ A target product profile for MRV-MNP, describing the key product attributes, has also been published.^15^

The MRV-MNP used in this trial contains live-attenuated MRV embedded in an array of microneedles. On application of the MNP to the skin, the microneedles penetrate the epidermis and upper dermis, dissolve, and release the vaccine. Application is designed to allow for administration by people who are not health-care professionals, and is largely painless.^16^ The MRV-MNPs are expected to have improved thermostability, facilitating vaccine administration beyond the end of the cold chain.^17^ They are a single-dose presentation, allowing every opportunity to vaccinate a child to be taken while minimising wastage from multi-dose vials. They do not generate sharps waste or risk sharps injury.^13^

This clinical trial was undertaken based on supportive preclinical data for MRV-MNP, and data on the use of the same dissolving MNP technology to deliver influenza vaccines to adults.^18^, ^19^ It aimed to assess the tolerability, safety, and immunogenicity of an MRV-MNP in adults, MRV-vaccinated toddlers aged 15–18 months, and MRV-naive infants aged 9–10 months. This trial provides the first data on the use of MNP to deliver vaccines to children.

## Methods

### Study design and participants

This single-centre, phase 1/2, double-blind, double-dummy, randomised, age de-escalation trial was conducted by the Medical Research Council Unit in The Gambia (MRCG). Participants were recruited at an MRCG clinical trial facility within the compound of Bundung Maternal and Child Health Hospital, located in the western region of The Gambia. Eligible adults aged 18–40 years, toddlers aged 15–18 months, and infants aged 9–10 months were recruited in series. To be eligible, participants had to be healthy according to the inclusion and exclusion criteria defined for the trial (appendix pp 3–5). All participants or parents or guardians of participants provided written informed consent. The study was approved by The Gambia Government/MRC Joint Ethics Committee (LEO 22420), the London School of Hygiene & Tropical Medicine Research Ethics Committee, and the Gambian Medicines Control Agency.

### Randomisation and masking

45 eligible adults were randomly assigned in a 2:1 ratio to receive either an MRV-MNP and a subcutaneous placebo injection (0·9% sodium chloride), or a placebo-MNP and an MRV by subcutaneous injection (MRV-SC). 120 eligible toddlers and 120 eligible infants were randomly assigned in a 1:1 ratio to the same combinations of active and placebo products. Random allocation was done using a predefined randomisation scheme generated by an independent statistician. Unmasked trial staff undertook random allocation using an online application, prepared the MNP and subcutaneous injection for administration, but had no role in endpoint data collection. The MRV-MNP and placebo-MNP were indistinguishable in appearance. The MRV and placebo subcutaneous injections were drawn into identical syringes and masked with opaque tape in case of minor colour differences, and thus were also indistinguishable in apprearance. Once prepared, the MNP and subcutaneous injection were handed to study nurses who were masked to the contents, who then applied the MNP and administered the subcutaneous injection. Participants, parents, and study staff assessing trial endpoints were also masked to treatment allocation.

### Procedures

At the screening visit (visit zero) participants were screened for eligibility and blood samples were collected for baseline safety bloods and immunogenicity endpoints (appendix p 9). At visit one (day 0), within 2 weeks of the screening visit, participants had final eligibility confirmed, were randomly allocated, and had the MNP administered followed by the subcutaneous injection. Participants had additional clinic visits on day 7 (visit two) and day 14 (visit three) after MNP administration, during which solicited adverse event data were reviewed, any new unsolicited adverse events were recorded, and safety bloods were collected (on days 7 and 14 for adults and on day 7 only for toddlers and infants). Additional clinic visits took place on day 42 (visit four) and day 180 (visit five), during which blood samples were collected for immunogenicity endpoints. Throughout the study, participants and parents were encouraged to contact the study team in the event of any illnesses, allowing study clinicians to assess, treat, and record all unsolicited adverse events. Additional immunisations according to the routine schedule in The Gambia were given to participants on or after the day 42 visit (appendix p 10).

Both the MRV-MNP (Micron Biomedical, Atlanta, GA, USA) and the single 0·5 mL dose of the MRV for subcutaneous injection (Serum Institute of India, Pune, India) contained not less than 1000 cell culture infectious dose (CCID_50_) of the live-attenuated Edmonston-Zagreb measles virus and not less than 1000 CCID_50_ of the live-attenuated Wistar RA 27/3 rubella virus. The bulk vaccine viruses (Serum Institute of India, Pune, India) were incorporated into dissolvable microneedles made up of pharmaceutical-grade excipients found in the US Food and Drug Administration's Inactive Ingredient Database for Approved Drug Products. The MRV-MNPs were designed to deliver similar doses of the vaccine viruses to those delivered by the subcutaneous injection. Full dissolution of the microneedles following application was confirmed by microscopy. The placebo-MNPs contained the same excipients as those contained in the MRV-MNP, but without the vaccine viruses. Good manufacturing practice was used throughout. The placebo for subcutaneous injection consisted of 0·5 mL of 0·9% weight by volume sterile sodium chloride (Hameln Pharmaceuticals, Gloucester, UK).

The MNP was applied to the dorsal aspect of the wrist for 5 min then removed. Participants were observed closely throughout this time to prevent the MNP from being disturbed. The subcutaneous injection was administered over the mid-deltoid region of the contralateral arm in adults and into the thigh in toddlers and infants. All subcutaneous injections were given with a 23 gauge, 25 mm, 0·5 mL auto-disable needle and syringe.

Solicited systemic adverse events and local adverse events at the MNP application site and subcutaneous injection site were collected and graded for severity (appendix pp 11–16) on the day of study product administration (day 0) and for a further 13 days, through home visits conducted by trained field workers. Unsolicited adverse events were collected from the day of administration until day 180, categorised by preferred term according to the Medical Dictionary for Regulatory Affairs and graded for severity (appendix p 17).

Safety haematology and biochemistry testing was done in the accredited MRCG clinical laboratories using validated assays. Serum was separated from blood samples, collected at baseline, day 42, and day 180, and frozen at below –70°C within 4 h before undergoing immunogenicity testing by the US Centers for Disease Control and Prevention laboratories (appendix p 18). Measles virus serum neutralising antibodies (SNA) were measured using a plaque reduction neutralisation test based on a WHO-recommended protocol.^20^ Rubella virus SNA were measured using a direct immunocolourimetric assay.^21^ Measles and rubella virus IgG was measured using a multiplex bead array.^22^ All antibody results were calibrated to appropriate WHO standards (appendix p 19).

Age de-escalation between cohorts was based on an unmasked review of all safety data to day 14 following study product administration in the preceding cohort by an independent data monitoring committee.

### Outcomes

The safety outcomes were the incidence and severity of solicited local adverse events (pain, erythema, and induration; with pruritus as an additional adverse event in the adult cohort) and systemic adverse events (fever, vomiting, diarrhoea, headache, fatigue, myalgia, arthralgia, and rash in adults; and fever, vomiting, diarrhoea, irritability, drowsiness, reduced appetite, and rash in toddlers and infants) on the day of study product administration and for a further 13 days; the incidence and severity of unsolicited adverse events (including serious adverse events) from the day of study product administration until 180 days; and the incidence and severity of biochemical and haematological laboratory abnormalities on day 7 and, in adults only, day 14 after study product administration. The relatedness of solicited systemic adverse events, unsolicited adverse events, and laboratory abnormalities, to study product administration was assessed (appendix p 20)

The immunogenicity outcomes were assessed using both SNA and IgG binding antibodies to measles and rubella, and were seroconversion rates (the percentage of participants who were seronegative at baseline and seropositive at day 42); rates of four-fold antibody rise (the percentage of participants who were seropositive at baseline and who had a four-fold increase in antibody concentrations by day 42); immune response rates (combining the number of participants undergoing seroconversion and experiencing a four-fold rise in antibodies); the percentage of participants who were seropositive; the geometric mean antibody concentrations (GMCs) at day 42 and day 180; and the geometric mean fold rise (GMFR) in antibody concentrations between baseline and day 42. Seropositivity was defined as antibody concentrations in international units (IUs) of 200 mIU/mL or greater for measles and 10 IU/mL or greater for rubella (appendix p 19).

### Statistical analysis

The trial was designed to provide descriptive data on the safety and immunogenicity of the MRV-MNP and comparator data on MRV-SC to guide product development decisions, rather than by a power calculation to test a formal statistical hypothesis. We chose 60 as our cohort size for infants and toddlers, as 60 participants provides a probability of 99**·**8% that at least one episode of a given safety event would occur, and a probability of 98**·**6% that at least two episodes of an event would occur in each of the given cohorts based on a true event rate of 10% in the vaccinated cohort (appendix pp 21–27). The safety population consists of all vaccinated participants, and analysis was conducted according to route of MRV administration, irrespective of subsequent protocol deviations. The primary immunogenicity population consisted of all vaccinated participants who had a baseline and day 42 visit result available and who had no protocol deviations considered to substantially impact on the immunogenicity endpoints. Descriptive 95% CIs without adjustment for multiplicity are provided throughout. CIs around proportions were calculated using the Wilson's score method without continuity correction,^23^ and CIs around difference in proportions were calculated using the Newcombe method without continuity correction.^24^ Having confirmed the log-normality assumption was appropriate, 95% CIs around measles and rubella GMCs and GMC ratios were calculated using the Student's *t* test for log_2_ transformed antibody concentrations. Analysis was conducted in R version 4.2.2. The trial was registered with the Pan African Clinical Trials Registry, PACTR202008836432905.

### Role of the funding source

The funder of the study had no role in data collection, analysis, interpretation, or in either writing of the report or the decision to submit for publication.

## Results

**Figure 1:**
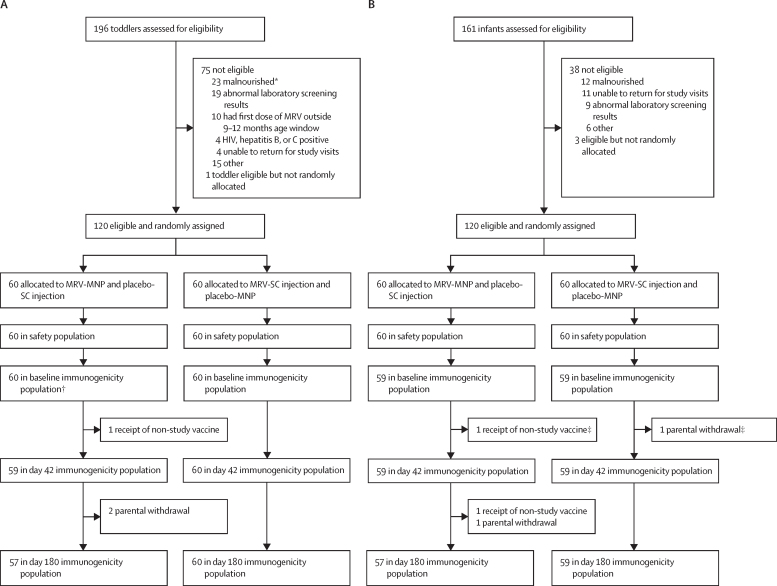
Trial profile—toddler and infant cohorts (A) Toddler cohort (B) Infant cohort. MNP=microneedle patch. MRV=measles and rubella vaccine. SC=subcutaneous. *Defined as weight-for-length Z score of <2 SDs below the mean. †In the toddler MRV-MNP group the baseline immunogenicity sample was analysed for the toddler who received a non-study vaccine between baseline and day 42, thus 60 baseline sample results were available. ‡One infant in the MRV-MNP group and one infant in the MRV-SC group were withdrawn between baseline and day 42. The baseline samples for these two infants were not analysed, hence 59 infants were included in the immunogenicity population at baseline as well as day 42.

Between May 18, 2021, and May 27, 2022, cohorts of 89 adults, 196 toddlers, and 161 infants were screened in series for enrolment into the trial. 45 (51%) adults were randomly allocated and vaccinated, all of whom were included in the primary immunogenicity population (appendix p 30). 120 (61%) toddlers were randomly allocated and vaccinated (figure 1A). 59 (98%) toddlers who received the MRV-MNP and placebo subcutaneous injection, and 60 (100%) toddlers who received the placebo-MNP and MRV-SC were included in the primary immunogenicity population. Of the infants, 120 (75%) were randomly allocated and vaccinated (figure 1B). 59 (98%) of the 60 infants in each of the MRV-MNP and placebo-MNP groups were included in the primary immunogenicity population. All vaccinated participants were included in the safety population. Baseline data for the adult cohorts are shown in the appendix (pp 31–35). There were no significant differences between the groups. 25 (83%) of 30 participants in the MRV-MNP group and 13 (87%) of 15 participants in the placebo-MNP group were male; 5 (17%) of 30 and 2 (13%) of 15 participants were female, respectively. All participants were of African origin. There were no acute allergic reactions in adults. Eight adults (27%) had a local solicited adverse event at the MRV-MNP application site, most commonly pruritus (5 [17%] of 30), compared with three adults (20%) at the placebo-MNP site (appendix p 49). All local reactions were mild and resolved without intervention. 15 adults (50%) had a mild or moderate systemic solicited event in the MRV-MNP group, compared with seven adults (47%) in the MRV-SC group. There were no severe local or systemic solicited adverse events in adults. 25 adults (83%) in the MRV-MNP group and all adults in the MRV-SC group had at least one unsolicited adverse event (appendix p 50). One adult (3%) in the MRV-MNP group had a severe unrelated serious adverse event, compared with two adults (13%) in the MRV-SC group (appendix p53). 16 adults (53%) had mild related adverse events at the MRV-MNP application site, most commonly discolouration in the form of hyperpigmentation (12 [40%]), compared with two (13%) at the placebo-MNP application site (appendix pp 50–51). All related events in adults resolved spontaneously before day 180 (appendix p 52). There were no related serious or severe adverse events in the adult cohort, and no clinically significant related changes in laboratory parameters on day 7 or day 14 in adults (appendix pp 32–36).

Based on SNA, measles GMC in the MRV-MNP group went from 242·7 mIU/mL (95% CI 143·4–410·7) at baseline to 1107·4 (839·3–1461·3) on day 42 (GMFR 4·6 [95% CI 3·0–6·8]), and in the MRV-SC group GMC went from 199·2 mIU/mL (95% CI 130·6–303·8) at baseline to 590·2 (424·9–819·7) on day 42 (GMFR 3·0 [95% CI 1·9–4·7]; appendix pp 55, 58). 15 (50% [95% CI 33·2–66·9]) of 30 adults had an immune response to measles following the MRV-MNP, compared with six (40% [19·8–64·3]) of 15 adults following MRV-SC.

In adults, rubella SNA GMC in the MRV-MNP group went from 172·0 IU/mL (95% CI 123·9–238·7) at baseline to 354·3 (302·1–415·6) on day 42 (GMFR 2·1 [95% CI 1·6–2·7]), and from 279·0 IU/mL (95% CI 194·4–400·3) at baseline to 342·0 (227·9–513·4) on day 42 (GMFR 1·2 [95% CI 0·8–1·8]) in the MRV-SC group. In total, four (13% [95% CI 5·3–29·7]) of 30 adults had an immune response to rubella following MRV-MNP compared with one (7% [1·2–29·8]) of 15 adults following the MRV-SC.

The median age of the toddlers was 15 months, and there was an equal split between males and females in both the MRV-MNP group and the placebo-MNP group (table 1). All the toddlers were African.

**Table 1:** Demographic and baseline data for the toddler and infant cohorts

	**Toddlers**		**Infants**	
	MRV-MNP and placebo SC (n=60)	MRV-SC and placebo MNP (n=60)	MRV-MNP and placebo SC (n=60)	MRV-SC and placebo MNP (n=60)
Age, months*				
Median (IQR)	15 (15 to 16)	15 (15 to 16)	9 (9 to 9)	9 (9 to 9)
**Sex**				
Male	30 (50%)	30 (50%)	26 (43%)	25 (42%)
Female	30 (50%)	30 (50%)	34 (57%)	35 (58%)
**Ethnicity**				
African	60 (100%)	60 (100%)	60 (100%)	60 (100%)
**Tribe**†				
Mandinka	39 (65%)	27 (45%)	35 (58%)	28 (47%)
Wolof	6 (10%)	7 (12%)	4 (7%)	7 (12%)
Fula	4 (7%)	9 (15%)	6 (10%)	3 (5%)
Jola	4 (7%)	12 (20%)	10 (17%)	7 (12%)
Other	7 (12%)	5 (8%)	5 (8%)	15 (25%)
**Weight, kg**‡				
Median (IQR)	8·9 (8·4 to 9·7)	9·0 (8·7 to 9·8)	8·2 (7·5 to 9·1)	8·0 (7·3 to 8·8)
**Length, cm**‡				
Median (IQR)	77·4 (75·5 to 79·4)	77·0 (75·4 to 79·5)	70·5 (69·3 to 72·5)	70·0 (68·7 to 72·0)
**Weight-for-length Z score**‡				
Median (IQR)	−0·9 (−1·6 to −0·5)	−1·0 (−1·4 to −0·2)	−0·3 (−0·8 to 0·5)	0·0 (−1·0 to 0·1)

Data are n (%) unless otherwise stated. MNP=microneedle patch. MRV=measles and rubella combined vaccines. SC=subcutaneous.

* On the day of consent.

† Some percentages might not add up to 100% owing to rounding.

‡ On the day of random allocation and vaccination.

**Figure 2:**
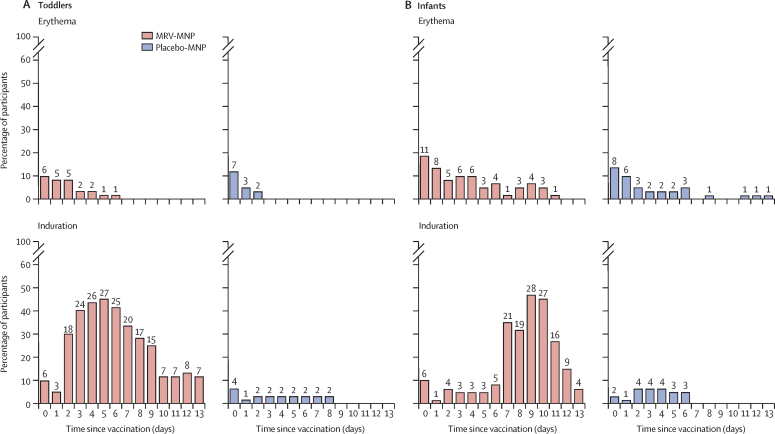
Local solicited adverse events—toddler and infant cohorts (A) Toddler cohort (B) Infant cohort. Numbers represent the absolute number of participants, from among the 60 in each randomisation group and cohort, affected on each day. All local reactions were mild in severity. In addition, one toddler had mild tenderness on day 8 following MRV-MNP and one toddler had mild tenderness on day 1 following placebo-MNP (data not shown graphically). MNP=microneedle patch. MRV=measles and rubella vaccine. SC=subcutaneous.

**Figure 3:**
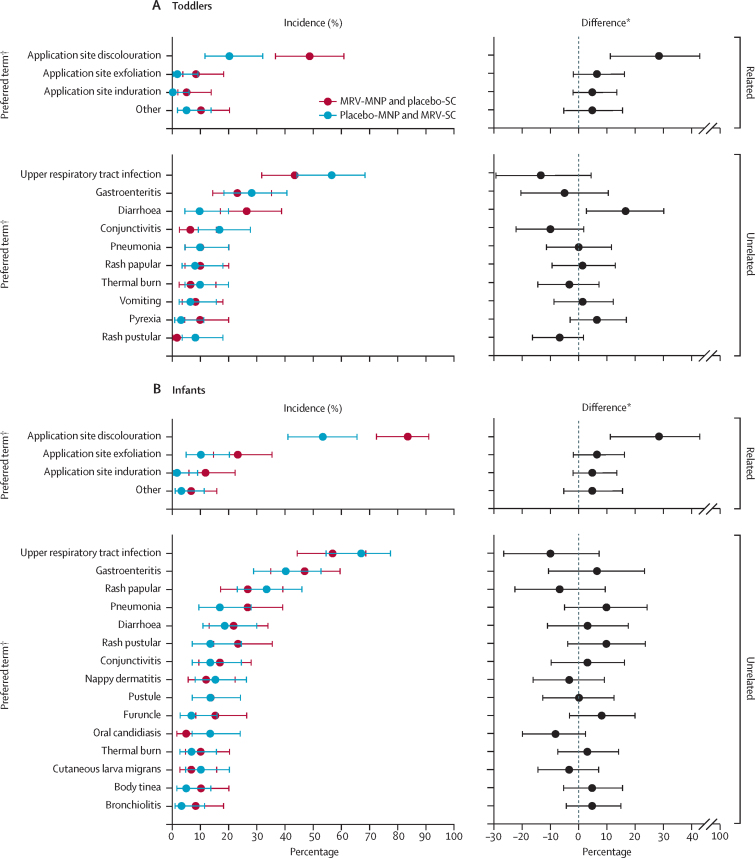
Unsolicited adverse events—toddler and infant cohorts (A) Toddler cohort (B) Infant cohort. Incidence or incidence difference and 95% CIs are shown. Only events which occurred in at least three participants in a given age cohort are included in the figure. MNP=microneedle patch. MRV=measles and rubella vaccine. SC=subcutaneous. *Percentage prevalence in MRV-MNP group minus percentage prevalence in placebo-MNP group. †Preferred term based on the Medical Dictionary for Regulatory Affairs.

There were no acute allergic reactions in toddlers (table 2). 50 toddlers (83%) had a mild local reaction at the MRV-MNP application site, compared with 18 toddlers (30%) at the placebo-MNP application site. Mild induration was the most common local reaction, and occurred in 46 (77%) toddlers who received the MRV-MNP compared with 9 (15%) of those who received the placebo-MNP. The incidence of mild induration in toddlers peaked at 45% (27 of 60) on day 5 following MRV-MNP application (figure 2A). Five toddlers (8%) had a fever following MRV-MNP, compared with 11 (18%) in the MRV-SC group. There was one severe fever (≥39·0°C) in a toddler in the MRV-SC group. 27 toddlers (45%) in the MRV-MNP group had a mild or moderate solicited systemic adverse event compared with 30 (50%) in the MRV-SC group. 59 toddlers (98%) had at least one of the 203 unsolicited adverse events reported following MRV-MNP, compared with 56 toddlers (93%) who had at least one of the 187 unsolicited adverse events reported in the MRV-SC group (table 3). Although more diarrhoea was reported in the MRV-MNP group, this is unlikely to be of clinical significance as an isolated finding (figure 3A). There was one (2%) unrelated serious adverse event in toddlers who received the MRV-MNP compared with eight unrelated events in seven toddlers (12%) in the MRV-SC group (appendix pp 53–54). 35 toddlers (58%) had at least one related unsolicited adverse event following MRV-MNP administration, compared with 16 toddlers (27%) following the placebo-MNP (table 3, figure 3A). Discolouration at the patch application site was the most common related unsolicited adverse event and was reported in 29 toddlers (48%) in the MRV-MNP group and 12 toddlers (20%) in the placebo-MNP group (figure 3A; table 3). All local related events were mild in severity. There was one moderate related adverse event, a generalised papular rash, in the MRV-MNP group. All application site related events in toddlers resolved before day 180 (appendix p 50). There were no related serious or severe adverse events in the toddler cohort. There were no clinically significant related changes in laboratory parameters in either group (appendix pp 37–42).

**Table 2:** Solicited safety events from days 0 to 13—toddler and infant cohorts

				**Toddlers**		**Infants**	
				MRV-MNP and placebo-SC, n=60	MRV-SC and placebo-MNP, n=60	MRV-MNP and placebo-SC, n=60	MRV-SC and placebo-MNP, n=60
Acute allergic reaction				0	0	0	0
Local solicited adverse events							
	MNP application site						
		Any local solicited event*					
			Total	50 (83%)	18 (30%)	46 (77%)	18 (30%)
			Mild (grade 1)	50 (83%)	18 (30%)	46 (77%)	18 (30%)
		Tenderness					
			Total	1 (2%)	1 (2%)	0	0
			Mild (grade 1)	1 (2%)	1 (2%)	0	0
		Erythema					
			Total	10 (17%)	9 (15%)	18 (30%)	14 (23%)
			Mild (grade 1)	10 (17%)	9 (15%)	18 (30%)	14 (23%)
		Induration					
			Total	46 (77%)	9 (15%)	39 (65%)	6 (10%)
			Mild (grade 1)	46 (77%)	9 (15%)	39 (65%)	6 (10%)
SC injection site							
	Any local solicited event*						
		Any reaction		8 (13%)	5 (8%)	2 (3%)	4 (7%)
			Mild (grade 1)	6 (10%)	5 (8%)	2 (3%)	4 (7%)
			Moderate (grade 2)	2 (3%)	0	0	0
Systemic solicited adverse events							
	Fever						
		Total		5 (8%)	11 (18%)	8 (13%)	4 (7%)
			Mild (grade 1)	1 (2%)	9 (15%)	5 (8%)	4 (7%)
			Moderate (grade 2)	4 (7%)	1 (2%)	3 (5%)	0
			Severe (grade 3)	0	1 (2%)	0	0
	Any systemic solicited event†						
		Total		27 (45%)	30 (50%)	31 (52%)	24 (40%)
			Mild (grade 1)	24 (40%)	23 (38%)	28 (47%)	23 (38%)
			Moderate (grade 2)	3 (5%)	7 (12%)	3 (5%)	1 (2%)

Data are n (%), where n=number of participants experiencing event by maximum severity grading. MRV=measles and rubella vaccine. MNP=microneedle patch. SC=subcutaneous.

* Tenderness, erythema, and induration.

† Vomiting, diarrhoea, irritability, drowsiness, reduced feeding, and rash.

**Table 3:** Unsolicited adverse events—toddler and infant cohorts

			**Toddlers**				**Infants**			
			MRV-MNP and placebo-SC, n=60		MRV-SC and placebo-MNP, n=60		MRV-MNP and placebo-SC, n=60		MRV-SC and placebo-MNP, n=60	
			n (%)	E	n (%)	E	n (%)	E	n (%)	E
**Adverse events**										
Total			59 (98%)	203	56 (93%)	187	60 (100%)	347	59 (98%)	285
	Mild (grade 1)		47 (78%)	190	38 (63%)	162	39 (65%)	315	44 (73%)	267
	Moderate (grade 2)		11 (18%)	12	13 (22%)	20	21 (35%)	32	14 (23%)	17
	Severe (grade 3)		1 (2%)	1	5 (8%)	5	0	0	1 (2%)	1
Serious adverse events			1 (2%)	1	7 (12%)	8	1 (2%)	1	1 (2%)	1
Adverse events resulting in discontinuation from the study			0	0	0	0	0	0	0	0
**Related adverse events**										
Total			35 (58%)	43	16 (27%)	16	57 (95%)	75	38 (63%)	41
	Mild (grade 1)		35 (58%)	42	16 (27%)	16	57 (95%)	75	38 (63%)	41
		MNP site discolouration	29 (48%)	29	12 (20%)	12	50 (83%)	50	32 (53%)	32
		MNP site exfoliation	5 (8%)	5	1 (2%)	1	14 (23%)	14	6 (10%)	6
		MNP site induration	3 (5%)	3	0	0	7 (12%)	7	1 (2%)	1
		Other	5 (8%)	5*	3 (5%)	3†	4 (7%)	4‡	2 (3%)	2§
	Moderate (grade 2)		1 (2%)	1¶	0	0	0	0	0	0
Related serious adverse events			0	0	0	0	0	0	0	0

Data are n (%), where n=number of participants experiencing event by maximum severity grading, unless otherwise stated. MRV=measles and rubella vaccine. MNP=microneedle patch. SC=subcutaneous. E=number of events by maximum severity grade.

* Two MNP site papules, one MNP site pruritus, two SC injection site induration.

† One MNP site papules, two generalised maculopapular rash.

‡ One MNP site macule, one generalised rash, one generalised papular rash; one poor infant feeding.

§ One MNP site papules, one diarrhoea.

¶ One generalised papular rash.

**Figure 4:**
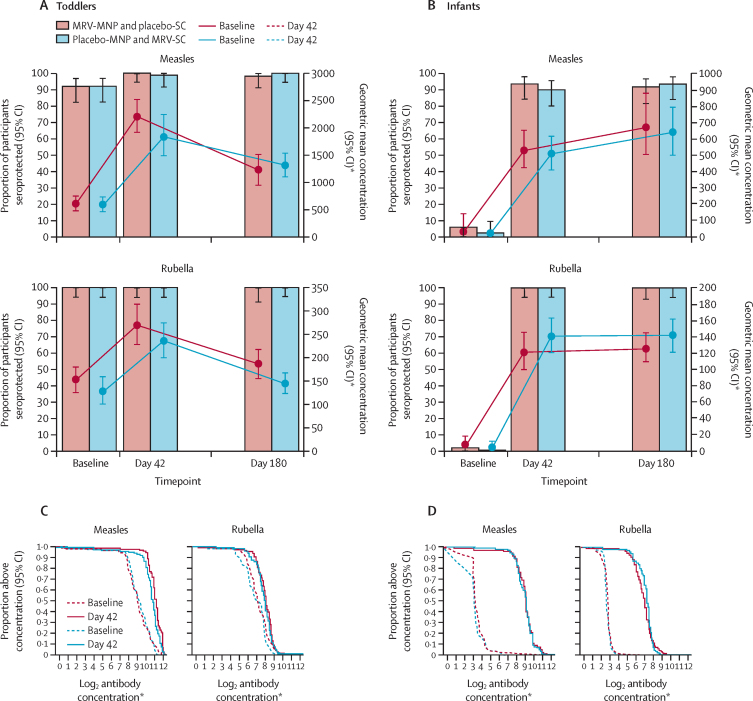
Serum neutralising antibody seroprotection levels, geometric mean antibody concentrations, and reverse cumulative distribution curves—toddler and infant cohorts Toddler cohort (A) and infant cohort (B) measles and rubella serum neutralising antibody seroprotection rates (solid bars) and 95% CIs. Seroprotection rates are defined as the percentage of evaluable participants with an antibody concentration higher than 200 mIU/mL for measles and higher than 10 IU/mL for rubella. Toddler cohort (C) and infant cohort (D) measles and rubella serum neutralising antibody baseline and day 42 reverse cumulative distributions curves. MNP=microneedle patch. MRV=measles and rubella vaccine. SC=subcutaneous. IU=international unit. *Measles geometric mean concentrations are measured in mIU/mL. Rubella geometric mean concentrations are reported in IU/mL

There were no notable differences in measles serological status between toddlers in the MRV-MNP group and toddlers in the MRV-SC group at baseline based on SNA (table 4; figure 4A). Over 90% of toddlers in both groups were seroprotected. Measles GMC went from 572·8 mIU/mL (95% CI 450·1–729·1) at baseline to 2182·9 mIU/mL (1905·6–2500·5) on day 42 (GMFR 3·8 [95% CI 3·0–4·9]) in the MRV-MNP group; and from 566·9 mIU/mL (95% CI 448·8–716·1) at baseline to 1811·5 mIU/mL (1480·6–2216·4) on day 42 (GMFR 3·2 [95% CI 2·5–4·1]) in the MRV-SC group. In total, 47% of toddlers (28 of 59; 95% CI 35·3–60·0) had an immune response to measles in the MRV-MNP group compared with 42% of toddlers (25 of 60; 95% CI 30·1–54·3) in the MRV-SC group. Measles GMC remained substantially above baseline at day 180.

**Table 4:** Measles and rubella serum neutralising antibodies—toddler and infant primary immunogenicity populations

		**Measles**			**Rubella**		
		MRV-MNP and placebo-SC	MRV-SC and placebo-MNP	Ratio*or difference†	MRV-MNP and placebo-SC	MRV-SC and placebo-MNP	Ratio*or difference†
**Toddlers**							
Baseline							
	Median (IQR)	489 (279 to 1159)	591 (319 to 961)	NA	152 (85 to 275)	151 (73 to 241)	NA
	GMC (95% CI)	572·8 (450·1 to 729·1)	566·9 (448·8 to 716·1)	1·01* (0·73 to 1·41)	151·6 (126·2 to 182·2)	126·4 (101·2 to 157·9)	1·20* (0·90 to 1·59)
	Seroprotection, n/N (%; 95% CI)	54/59 (92%; 81·7 to 96·3)	55/60 (92%; 81·9 to 96·4)	0·0† (−11·1 to 10·7)	59/59 (100%; 93·9 to 100·0)	60/60 (100%; 94·0 to 100·0)	0·0†(−6·1 to 6·0)
Visit 4 (day 42)							
	Median (IQR)	2222 (1678 to 3447)	1791 (1284 to 2807)	NA	278 (182 to 406)	247 (174 to 338)	NA
	GMC (95% CI)	2182·9 (1905·6 to 2500·5)	1811·5 (1480·6 to 2216·4)	1·21* (0·95 to 1·53)	268·2 (228·3 to 315·0)	234·3 (199·6 to 274·9)	1·14* (0·91 to 1·43)
	GMFR (95% CI)	3·8 (3·0 to 4·9)	3·2 (2·5 to 4·1)	1·19* (0·85 to 1·68)	1·8 (1·4 to 2·2)	1·9 (1·5 to 2·9)	0·96* (0·71 to 1·28)
	Seroprotection, n (%; 95% CI)	59/59 (100%; 94·0 to 100·0)	59/60 (98%; 91·1 to 99·7)	1·7† (−4·6 to 8·9)	59/59 (100%; 93·9 to 100)	60/60 (100%; 94·0 to 100)	0·0†(−6·1 to 6·0)
	Baseline seronegative, n	5	5	NA	0	0	NA
	Seroconversion, n/N (%; 95% CI)	5/5 (100%; 56·6 to 100·0)	4/5 (80%; 37·6 to 96·4)	20·0† (−26·4 to 62·5)	NA	NA	NA
	Baseline seropositive, n	54	55	NA	59	60	NA
	Four-fold rise, n/N (%; 95% CI)	23/54 (43%; 30·3 to 55·8)	21/55 (38%; 26·5 to 51·4)	4·4† (−13·6 to 22·1)	5/59 (8%; 3·7 to 18·4)	8/60 (13%; 6·9 to 24·2)	−4·9† (−16·7 to 6·9)
	Immune response n/N (%; 95% CI)	28/59 (47%; 35·3 to 60·0)	25/60 (42%; 30·1 to 54·3)	5·8† (−11·8 to 22·9)	5/59 (8%; 3·7 to 18·4)	8/60 (13%; 6·9 to 24·2)	−4·9† (−16·7 to 6·9)
Visit 5 (day 180)							
	Median (IQR)	1311 (654 to 2048)	1203 (844 to 1961)	NA	181 (110 to 261)	141 (98 to 224)	NA
	GMC (95% CI)	1195·2 (958·7 to 1489·9)	1290·8 (1086·9 to 1532·8)	0·93* (0·70 to 1·22)	183·3 (154·9 to 216·9)	142·2 (121·8 to 166·1)	1·29* (1·03 to 1·62)
	Seroprotection n/N (%; 95% CI)	56/57 (98%; 90·7 to 99·7)	60/60 (100%; 94·0 to 100·0)	−1·8† (−9·3 to 4·4)	57/57 (100%; 93·7 to 100·0)	60/60 (100%; 94·0 to 100·0)	0·0†(−6·3 to 6·0)
**Infants**							
Baseline							
	Median (IQR)	8 (7 to 12)	7 (6 to 9)	NA	6 (5 to 6)	5 (5 to 6)	NA
	GMC (95% CI)	12·8 (9·5 to 17·2)	11·3 (8·5 to 15·1)	1·13* (0·75 to 1·70)	6·9 (6·4 to 7·4)	6·5 (6·2 to 6·9)	1·05* (0·96 to 1·15)
	Seroprotection n (%; 95% CI)	3/59 (5%;1·7 to 13·9)	1/59 (2%;0·3 to 9·0)	3·4† (−4·6 to 12·3)	1/59 (2%; 0·3 to 9·0)	0/59 (0·0 to 6·1)	1·7†(−4·6 to 9·0)
Visit 4 (day 42)							
	Median (IQR)	505 (309 to 716)	494 (311 to 671)	NA	123 (74 to 176)	156 (113 to 201)	NA
	GMC (95% CI)	520·9 (420·8 to 644·9)	495·2 (402·5 to 609·3)	1·05* (0·78 to 1·41)	120·3 (99·9 to 144·9)	140·3 (120·9 to 162·7)	0·86* (0·68 to 1·09)
	GMFR (95% CI)	40·8 (32·3 to 51·5)	43·7 (34·8 to 54·8)	0·93* (0·68 to 1·29)	17·5 (14·2 to 21·5)	21·4 (18·5 to 24·9)	0·82* (0·63 to 1·05)
	Seroprotection n/N (%; 95% CI)	55/59 (93%; 83·8 to 97·3)	53/59 (90%; 79·5 to 95·3)	3·4† (−7·5 to 14·5)	59/59 (100%; 93·9 to 100·0)	59/59 (100%; 93·9 to 100·0)	0·0†(−6·1 to 6·1)
	Baseline seronegative, n	56	58	NA	56	59	NA
	Seroconversion n (%; 95% CI)	52/56 (93%; 83·0 to 97·2)	52/58 (90%; 79·2 to 95·2)	3·2† (−8·1 to 14·5)	58/58 (100%; 93·8 to 100·0)	59/59 (100%; 93·9 to 100·0)	0·0†(−6·2 to 6·1)
	Baseline seropositive, n	3	1	NA	1	0	NA
	Four-fold rise n/N (%; 95% CI)	1/3 (33%; 6·2 to 79·2)	0/1 (0·0 to 79·4)	33·3† (−50·5 to 79·2)	0/1 (0·0 to 79·4)	NA	NA
	Immune response n/N (%; 95% CI)	53/59 (90%; 79·5 to 95·3)	52/59 (88%; 77·5 to 94·1)	1·7† (−10·2 to 13·7)	58/59 (98%; 91·0 to 99·7)	59/59 (100%; 93·9 to 100·0)	−1·7^‡^ (−9·0 to 4·6)
Visit 5 (day 180)							
	Median (IQR)	654 (347 to 1100)	706 (329 to 985)	NA	125 (89 to 178)	139 (94 to 216)	NA
	GMC (95% CI)	661·2 (501·5 to 871·9)	629·0 (498·7 to 793·5)	1·05* (0·74 to 1·50)	125·1 (109·0 to 143·6)	140·7 (122·1 to 161·9)	0·89* (0·73 to 1·08)
	Seroprotection n/N (%; 95% CI)	52/57 (91%; 81·1 to 96·2)	55/59 (93%; 83·8 to 97·3)	−2·0† (−13·0 to 8·6)	57/57 (100%; 93·7 to 100·0)	59/59 (100%; 93·9 to 100·0)	0·0†(−6·3 to 6·1)

Baseline and visit 4 (day 42) analysis are in the primary immunogenicity population; visit 5 (day 180) analysis is in the day 180 secondary immunogenicity population; seroconversion is defined as a change from seronegative at baseline to seropositive at day 42; four-fold rise is defined as a four-fold rise in antibody concentrations between baseline and day 42 among individuals who were seropositive at baseline; immune response includes all those who were seronegative at baseline and seroconverted on day 42 or who were seropositive at baseline and had a four-fold rise in antibody concentrations; for measles, seronegative is defined as an antibody concentration of <200 mIU/mL, seropositive or seroprotection is defined as an antibody concentration of ≥200 mIU/mL; for rubella, seronegative is defined as an antibody concentration of <10 IU/mL and seropositive or seroprotection is defined as an antibody concentration of ≥10 IU/mL. MRV=measles and rubella vaccine. MNP=microneedle patch. SC=subcutaneous. NA=not applicable. IU=international unit. GMC=geometric mean antibody concentrations reported in mIU/mL for measles and IU/mL for rubella. GMFR=geometric mean fold rise.

* Ratio [MNP]/[SC injection].

† Difference [MNP] – [SC injection]. Estimates are presented with 95% CIs. CIs for the log_2_ transformed means assume a Student's *t* test. CIs for seroprotection and seroconversion were calculated using the Wilson score method without continuity correction. CIs for differences between proportions were calculated using the Newcombe method without continuity correction.

All the toddlers were rubella seroprotected at baseline based on SNA concentrations (table 4; figure 4A). Rubella GMC went from 151·6 IU/mL (95% CI 126·2–182·2) at baseline to 268·2 IU/mL (228·3–315·0) on day 42 (GMFR 1·8 [95% CI 1·4–2·2]) in the MRV-MNP group and from 126·4 IU/mL (95% CI 101·2–157·9) at baseline to 234·3 IU/mL (95% CI 199·6–274·9) on day 42 (GMFR 1·9 [95% CI 1·5–2·9]) in the MRV-SC group. Reflecting high baseline antibody titres, under 14% of toddlers had an immune response to rubella in either the MRV-MNP group or the MRV-SC group. Rubella GMC returned towards baseline levels in both groups by day 180.

The median age of the infants was 9 months, and the sex ratio was 34 (57%) female and 26 (43%) male in the MRV-MNP group, and 35 (58%) female and 25 (42%) male in the placebo-MNP groups (table 1).

There were no acute allergic reactions in infants (table 2). 46 infants (77%) had a mild local reaction at the MRV-MNP application site compared with 18 (30%) at the placebo-MNP application site. Mild induration at the MNP application site was the most common local reaction, and was observed in 39 infants (65%) who received the MRV-MNP compared with 6 (10%) of those who received the placebo-MNP. The incidence of mild induration in infants peaked at 47% (28 of 60) on day 9 following MRV-MNP application (figure 2B). There were no moderate or severe reactions at either the MRV-MNP or placebo-MNP application site. Eight infants (13%) in the MRV-MNP group had a fever compared with four infants (7%) in the MRV-SC group. There were no severe fevers in the infant cohort. 31 infants (52%) in the MRV-MNP group had a mild or moderate solicited systemic adverse event compared with 24 (40%) in the MRV-SC group. All infants in the MRV-MNP group had at least one of the 347 unsolicited adverse events reported compared with 59 infants (98%) who had at least one of the 285 unsolicited adverse events in the MRV-SC group (table 3). There were no notable trends in the incidence of specific adverse events, judged to be unrelated to vaccination, comparing the MRV-MNP and placebo-MNP groups (figure 3B). There was one (2%) unrelated serious adverse event in an infant in each group (appendix p 54). 57 infants (95%) had at least one related unsolicited adverse event in the MRV-MNP group compared with 38 infants (63%) following the placebo-MNP (table 3; figure 3B). Discolouration at the MNP application site (all hyperpigmentation) was the most common related adverse event and was reported in 50 infants (83%) in the MRV-MNP group and 32 infants (53%) in the placebo-MNP group on day 14. Five infants (8%) in the MRV-MNP group had ongoing application site discolouration on day 180; all related events in the placebo-MNP group had resolved at the same timepoint (appendix p 52). There were no related serious or severe adverse events in the infant cohort. There were no clinically significant, related changes in laboratory parameters in either group (appendix pp 43–48).

Three of 59 infants (5% [95% CI 1·7–13·9]) in the MRV-MNP group and one of 59 (2% [0·3–9·0]) in the MRV-SC group were seroprotected against measles at baseline (figure 4B, table 4). Seroconversion occurred in 93% of infants (52/56 [95% CI 83·0–97·2]) in the MRV-MNP group compared with 90% (52/58 [95% CI 79·2–95·2]) in the MRV-SC group. Measles GMCs on day 42 were 520·9 mIU/mL (95% CI 420·8–644·9) in the MRV-MNP group and 495·2 mIU/mL (402·5–609·3) in the MRV-SC group. In infants, the SNA GMCs continued to increase to day 180 in both groups. Seroprotection rates at day 180 were 91% (52/57 [95% CI 81·1–96·2]) in the MRV-MNP group and 93% (55/59 [83·8–97·3]) in the MRV-SC group.

One of 59 infants (2% [95% CI 0·3–9·0]) in the MRV-MNP group was seroprotected against rubella at baseline (figure 4B, table 4B). All infants in the MRV-SC group were seronegative. All infants in both groups seroconverted to rubella by day 42 (MRV-MNP group 100% [95% CI 93·8–100] and MRV-SC group 100% [93·9–100]) and remained seropositive at day 180. Rubella GMCs on day 42 were 120·3 IU/mL (95% CI 99·9–144·9) in the MRV-MNP group and 140·3 IU/mL (120·9–162·7) in the MRV-SC group, and were similar at day 180.

Reverse cumulative distribution curves illustrate the alignment of the antibody distribution between MRV-MNP and MRV-SC groups before and after vaccine administration (figure 4C, D; appendix p 58). Data on measles and rubella IgG antibody responses are provided (appendix pp 58–63) and provide a similar picture to the SNA responses.

## Discussion

This phase 1/2 trial provides the first data on the use of MNP to administer vaccines to children. In adults, toddlers, and infants the MRV-MNP was well tolerated and safe. The immunogenicity of the MRV when administered by MNP was similar to the immunogenicity of the vaccine when administered subcutaneously by needle and syringe. The results are consistent with preclinical studies which showed the MRV-MNP generates protective SNA responses in non-human primates.^18^

Given the delivery of the vaccine viruses directly into the skin, mild local reactions at the MRV-MNP application site were more frequent than at the subcutaneous injection site. Induration was the most common local solicited event in toddlers and infants. The earlier peak in incidence in the toddlers (day 5) compared with the infants (day 9) suggests an anamnestic infiltration of cells primed by the previous measles and rubella vaccination in toddlers, while the timing of the response in the infants is consistent with a naive response driven by viral replication.^25^, ^26^ Despite this, it is reassuring that all the reactions were mild and of no safety concern in either group.

Application site hyperpigmentation occurred most frequently following the MRV-MNP, although it also occurred following placebo-MNP, so was not solely driven by responses to the vaccine viruses. Diverse inflammatory and other soluble mediators have been shown to increase melanin production, while post-inflammatory hyperpigmentation is also more frequent in dark skin.^27^ This is the first trial of MNP in an African population, and the findings suggest that transient hyperpigmentation should be expected and any effect on MNP acceptability should continue to be explored beyond this trial.

The doses of the attenuated viruses delivered by MNP and subcutaneous injection were similar, although the potential for antigen dose-sparing, through the delivery of vaccine by the intradermal route, directly to a network of antigen-presenting cells rather than by the subcutaneous route, warrants future consideration.^28^, ^29^ The measles seroconversion rate of 93% in infants who had the vaccine delivered by MNP is comparable to the rates reported in the published literature following subcutaneous delivery of the vaccine. Between 85% and 90% of infants are expected to seroconvert following vaccination at 9 months, with the figure rising to 90% to 95% in children vaccinated at age 12 months.^25^ Similarly, a 2021 meta-analysis reported a seroconversion rate of 99% (95% CI 98–99) in children following a single dose of a rubella vaccine containing the RA 27/3 strain and is consistent with our findings.^30^ Serum neutralising antibodies are considered to be the gold-standard correlate of protection for both vaccines, although the data linking immunogenicity and effectiveness are scarce.^25^, ^26^ However, the robust and comparable immune responses to MNP and subcutaneous vaccine administration are reassuring and also support further development of this MNP technology.

The 5 min wear time for the patch aligns with the minimum requirements set out in the target product profile for MRV-MNP.^15^ The document also emphasises that a reduction in wear time would be preferable to reduce the risk of premature removal by infants and toddlers. Premature removal is also likely to be influenced by the anatomical site of application. Generating empirical data to further understand these risks and to fully understand the programmatic effect of both wear time and site for routine immunisations and SIA is essential if the potential value of the MRV-MNP is to be maximised. Future clinical studies will evaluate a shorter wear time (eg, ≤1 min).

The trial had several strengths. The use of the double-dummy design (which allowed the staff applying the MNP and administering the subcutaneous injection, participants and parents, and all staff collecting study endpoints to be masked to allocation group) minimises the risks of performance bias during MNP application and of observer bias related to the collection of safety endpoints. The design also reduces the risk of novelty bias, where there is a tendency to report treatments as being better based on the fact they are new, which in this case could have affected safety endpoint data.^31^ Conducting the study in The Gambia also maximises the relevance of the findings to a key future target population in west Africa. The MRV used in the study has been given to many millions of children globally and is known to provide reliable protection in those who seroconvert. Thus, the immunogenicity readouts used in the trial are expected to translate into future effects on disease endpoints.

The trial had several limitations which predominantly reflect its early phase design. Although it is the largest trial of MNP conducted to date and the only trial in children, the samples size was relatively small. The analysis is descriptive and does not exclude statistically or clinically significant differences in safety and immunogenicity endpoints becoming apparent in larger trials. The eligibility criteria were deliberately restrictive in all age groups. The recruitment of healthy participants aimed to minimise the occurrence of unrelated safety events, thus increasing the chances of detecting low-level safety signals. Nonetheless, future trials should be as representative as possible, in particular including malnourished children and other vulnerable groups. Generating data in children aged 6 months will also be important in due course, considering the recommended use of MRV from this age in outbreaks.

In summary, this trial reports the first data on the use of MNP to deliver vaccines to children and infants. The MNP technology has recently been ranked as being the highest global innovation priority for achieving equity in vaccination coverage in low-income and middle-income countries, while MRV-MNPs are widely considered to be potentially instrumental for measles and rubella elimination. The tolerability, safety and immunogenicity data generated support accelerating the development of this key technology.

## Data sharing

Individual participant data will be shared after de-identification and made available from 3 months after publication until 3 years after publication. Clinical documents, including the study protocol, statistical analysis plan, and informed consent form will be available immediately after publication. Researchers who provide a scientifically sound proposal to the corresponding author and sign a data access agreement will receive access to individual participant data. Proposals will be reviewed and approved by the investigator, sponsor (Micron Biomedical), and collaborators based on scientific merit.

## Declaration of interests

SH, DVM, MRP, and MR are employees of, or affiliated with, Micron Biomedical. All other authors declare no competing interests. The findings and conclusions in this report are those of the authors and do not necessarily represent the official position of the Centers for Disease Control and Prevention.

## References

[1] Minta AA, Ferrari M, Antoni S (2022). Progress toward regional measles elimination - worldwide, 2000–2021. MMWR Morb Mortal Wkly Rep.

[2] Zimmerman LA, Knapp JK, Antoni S, Grant GB, Reef SE (2022). Progress toward rubella and congenital rubella syndrome control and elimination - worldwide, 2012–2020. MMWR Morb Mortal Wkly Rep.

[3] WHO (Feb 23, 2021). Measles and rubella strategic framework: 2021–2030. https://measlesrubellainitiative.org/measles-rubella-strategic-framework-2021-2030/.

[4] WHO (2017). Measles vaccines: WHO position paper - April 2017. Wkly Epidemiol Rec.

[5] Winter AK, Moss WJ (2022). Rubella. Lancet.

[6] Wariri O, Nkereuwem E, Erondu NA (2021). A scorecard of progress towards measles elimination in 15 west African countries, 2001–19: a retrospective, multicountry analysis of national immunisation coverage and surveillance data. Lancet Glob Health.

[7] Utazi CE, Wagai J, Pannell O (2020). Geospatial variation in measles vaccine coverage through routine and campaign strategies in Nigeria: analysis of recent household surveys. Vaccine.

[8] Orenstein WA, Hinman A, Nkowane B, Olive JM, Reingold A (2018). Measles and rubella global strategic plan 2012–2020 midterm review. Vaccine.

[9] Verguet S, Johri M, Morris SK, Gauvreau CL, Jha P, Jit M (2015). Controlling measles using supplemental immunization activities: a mathematical model to inform optimal policy. Vaccine.

[10] Chopra M, Bhutta Z, Chang Blanc D (2020). Addressing the persistent inequities in immunization coverage. Bull World Health Organ.

[11] Portnoy A, Jit M, Helleringer S, Verguet S (2020). Comparative distributional impact of routine immunization and supplementary immunization activities in delivery of measles vaccine in low- and middle-income countries. Value Health.

[12] Postolovska I, Helleringer S, Kruk ME, Verguet S (2018). Impact of measles supplementary immunisation activities on utilisation of maternal and child health services in low-income and middle-income countries. BMJ Glob Health.

[13] Hasso-Agopsowicz M, Crowcroft N, Biellik R (2022). Accelerating the development of measles and rubella microarray patches to eliminate measles and rubella: recent progress, remaining challenges. Front Public Health.

[14] Gavi, the Vaccine Alliance (Feb 9, 2024). Vaccine Innovation Prioritisation Strategy (VIPS). https://www.gavi.org/our-alliance/market-shaping/vaccine-innovation-prioritisation-strategy.

[15] UNICEF (June, 2019). Measles-rubella microarray patch (MR-MAP) target product profile. https://www.unicef.org/supply/target-product-profile-measles-rubella-microarray-patch.

[16] Arya J, Henry S, Kalluri H, McAllister DV, Pewin WP, Prausnitz MR (2017). Tolerability, usability and acceptability of dissolving microneedle patch administration in human subjects. Biomaterials.

[17] Kagucia EW, Ochieng B, Were J (2021). Impact of mobile phone delivered reminders and unconditional incentives on measles-containing vaccine timeliness and coverage: a randomised controlled trial in western Kenya. BMJ Glob Health.

[18] Joyce JC, Carroll TD, Collins ML (2018). A microneedle patch for measles and rubella vaccination is immunogenic and protective in infant rhesus macaques. J Infect Dis.

[19] Rouphael NG, Paine M, Mosley R (2017). The safety, immunogenicity, and acceptability of inactivated influenza vaccine delivered by microneedle patch (TIV-MNP 2015): a randomised, partly blinded, placebo-controlled, phase 1 trial. Lancet.

[20] Cohen BJ, Audet S, Andrews N, Beeler J (2007). Plaque reduction neutralization test for measles antibodies: description of a standardised laboratory method for use in immunogenicity studies of aerosol vaccination. Vaccine.

[21] Lambert ND, Haralambieva IH, Kennedy RB, Ovsyannikova IG, Pankratz VS, Poland GA (2015). Polymorphisms in HLA-DPB1 are associated with differences in rubella virus-specific humoral immunity after vaccination. J Infect Dis.

[22] Coughlin MM, Matson Z, Sowers SB (2021). Development of a measles and rubella multiplex bead serological assay for assessing population immunity. J Clin Microbiol.

[23] Newcombe RG (1998). Two-sided confidence intervals for the single proportion: comparison of seven methods. Stat Med.

[24] Newcombe RG (1998). Interval estimation for the difference between independent proportions: comparison of eleven methods. Stat Med.

[25] WHO (2020). The immunological basis for immunization series: module 7: measles: update. https://apps.who.int/iris/handle/10665/331533.

[26] WHO (2008). The immunological basis for immunization series: module 11: rubella: update. https://apps.who.int/iris/handle/10665/43922.

[27] Fu C, Chen J, Lu J (2020). Roles of inflammation factors in melanogenesis. Mol Med Rep.

[28] Beals CR, Railkar RA, Schaeffer AK (2016). Immune response and reactogenicity of intradermal administration versus subcutaneous administration of varicella-zoster virus vaccine: an exploratory, randomised, partly blinded trial. Lancet Infect Dis.

[29] Teunissen MB, Haniffa M, Collin MP (2012). Insight into the immunobiology of human skin and functional specialization of skin dendritic cell subsets to innovate intradermal vaccination design. Curr Top Microbiol Immunol.

[30] van den Boogaard J, de Gier B, de Oliveira Bressane Lima P (2021). Immunogenicity, duration of protection, effectiveness and safety of rubella containing vaccines: a systematic literature review and meta-analysis. Vaccine.

[31] Persaud N, Heneghan C Novelty bias. Catalogue Of Bias. https://catalogofbias.org/biases/novelty-bias/.

