# Supplementary material for: A measles and rubella vaccine microneedle patch in The Gambia: a phase 1/2, double-blind, double-dummy, randomised, active-controlled, age de-escalation trial
Source: Lancet. 2024 May 11;403(10439):1879–92. doi: 10.1016/S0140-6736(24)00532-4 (PMC11099471; doi:10.1016/S0140-6736(24)00532-4)
Supplement: Supplementary appendix [file mmc1.pdf]

THE LANCET

Supplementary appendix

This appendix formed part of the original submission and has been peer reviewed. We post it as supplied by the authors.

Supplement to: Adigweme I, Yisa M, Ooko M, et al. A measles and rubella vaccine microneedle patch in The Gambia: a phase 1/2, double-blind, double-dummy, randomised, active-controlled, age de-escalation trial. *Lancet* 2024; published online April 29. https://doi.org/10.1016/S0140-6736(24)00532-4.

Contents

Eligibility criteria 3
Inclusion criteria 3
Exclusion criteria 5
Study schedule..... 9
Adults 9
Toddlers and infants..... 9
Additional immunizations administered to participants by age cohort. 10
Local and systemic adverse event grading..... 11
Adult cohort: local solicited adverse event grading 11
Adult cohort: systemic solicited adverse event grading 12
Toddler and infant cohort: local solicited adverse event grading..... 14
Toddler and infant cohort: systemic solicited adverse event grading 15
Severity grading for unsolicited adverse events 17
CDC Statement: 18
International standards used to calibrate measles and rubella serological results..... 19
Measles: 19
Rubella: 19
Unsolicited adverse event relatedness assessment..... 20
Sample size considerations. 21
Safety and tolerability..... 21
Immunogenicity 26
Analysis populations and missing data 28
Safety population 28
Immunogenicity populations..... 28
Primary immunogenicity population 28
Secondary immunogenicity population 28

Supplementary Figure: Trial profile - adult cohort ... 30

Supplementary Table: Demographic and baseline anthropometric data – adult cohort ... 31

Supplementary Figure: Safety laboratory parameters ... 32

- Adult cohort ... 32
- Toddler cohort ... 37
- Infant cohort ... 43

Supplementary Table: Solicited safety data, day 0 to 13 – adult cohort ... 49

Supplementary Table: Unsolicited adverse event data – adult cohorts ... 50

Supplementary Figure: Unsolicited adverse events – adult cohort ... 51

Supplementary Table: Duration of related unsolicited MNP adverse events ... 52

- Adults ... 52
- Toddlers ... 52
- Infants ... 52

Supplementary Table: Serious adverse event listing ... 53

Supplementary Table: Measles and rubella serum neutralizing antibody data – adult primary immunogenicity population ... 55

Supplementary Figure: Typical mild MRV-MNP application site reaction time series (single infant). ... 57

Supplementary Table: Measles and rubella IgG antibody data – adult primary immunogenicity population ... 58

Supplementary Table: Measles and rubella IgG antibody data – toddler and infant primary immunogenicity populations ... 60

Supplementary Figure: Serum neutralizing antibody seroprotection and geometric mean antibody concentrations – adult cohort ... 63

Supplementary references. ... 64

Eligibility criteria

Participants must meet all the inclusion criteria and none of the exclusion criteria to be eligible to participate. No screening procedures will take place before an individual has provided written informed consent to join the trial.

Inclusion criteria

A prospective participant must meet all the following inclusion criteria to be eligible for enrolment (randomization and vaccination):

Participants must:

- Provide voluntary written/thumb-printed informed consent for trial participation (adult cohort)
- Have voluntary written/thumb-printed informed consent provided for them by a parent (toddler and infant cohort)
- Be between 18 and 40 years inclusive on the day of consent. They will be eligible from the day they reach 18 years of age until the day before they reach 41 years of age (adult cohort)
- Be between 15 and 18 months of age inclusive on the day of consent. They will be eligible from the day they reach 15 months of age until the day before they reach 19 months of age (toddler cohort)
- Be between 9 and 10 months of age inclusive on the day of consent. They will be eligible form the day they reach nine months of age until the day before they reach 11 months of age (infant cohort).
- The identity and age of all prospective participants must be as confirmed from a suitable source document prior to informed consent. Suitable source documents include but are not limited to the birth certificate, national identification card, passport and, in toddlers and infants, the parent held infant welfare card (IWC). Photographic identification is not consistently available and is not required. Participants/parents will be issued with a trial photographic identification card once randomization and vaccination has taken place (V1).
- Be judged to be able to comprehend and comply with study requirements and procedures and must be willing and able to return for all scheduled follow-up visits (adult cohort)
- Have a parent who is judged to be able to comprehend and comply with study requirement and procedures and is willing and able to return for all scheduled follow-up visits (toddler and infant cohort).

- Be willing to avoid consumption (ingestion and topical application) of herbal or other local traditional medications throughout the course of the study. Also be willing to avoid the use of medications (for example those available for purchase at local pharmacies) except those provided by the trial team (unless in an emergency) (adult cohort).

- Have a parent who is willing to ensure they avoid consumption (ingestion and topical application) of herbal or other local traditional medications throughout the course of the study. Also, who is willing to ensure they avoid the use of medications (for example those available for purchase at local pharmacies) except those provided by the trial team (unless in an emergency) (toddler and infant cohort).

- Have a readily identifiable place of residence within a reasonable travelling distance of the clinical trial site.

- This aims to ensure home visits for solicited AE can be undertaken reliably and that the participant is able to present to the trial site or be reviewed at their home in the event of other unsolicited health complaints. No specific geographical limits are set with this regard. Rather such decisions will be made based on the judgement of senior members of the field team based on their detailed knowledge of local geography and transport links.

- Have a consistent means of telephone contact for the duration of trial participation (adult cohort).

- Have a parent with a consistent means of telephone contact for the duration of trial participation (toddler and infant cohort).

- A telephone on a closed user group (CUG) network with the field team will be provided to participants/parents to ensure they are able to contact the investigator team at any time day or night without the need for telephone credit.

- Have a site on one wrist that is judged to be suitable for MNP administration.

- Adult female cohort only: have a negative serum pregnancy test at screening (V0) and negative urine pregnancy test on the day of vaccination (V1).

- Adult female cohort only: employ an effective method of birth control for two months preceding and throughout the study.

- Effective methods of birth control are defined as follows: credible history of continuous abstinence from heterosexual activity as a normal lifestyle choice, hormonal contraceptives (oral, injectable,

implant, patch, and ring), barrier contraceptives (condom or diaphragm, with spermicide) and intrauterine device. When using contraceptives, participants must have been using their current contraceptive for the past 2 months to be eligible. Adult female participants with documented sterilization via tubal ligation or hysterectomy may be enrolled although for completeness all female participants will undergo pregnancy testing as outlined above. Participants will never be encouraged to start using contraception in order to allow them to be eligible to join the study.

- Toddler cohort only: have been parenterally vaccinated against measles and rubella at between nine and 12 months of age (see exclusion criteria section 0 for vaccination requirements in the adult and infant cohorts).

- Be willing to avoid measles and rubella vaccine administration for the duration of enrolment in the study, including in the case of a national measles and rubella vaccination campaign in The Gambia (adult cohort)

- Have a parent who is willing to ensure they avoid measles and rubella vaccine administration for the duration of enrolment in the study, including in the case of a national measles and rubella vaccination campaign in The Gambia (toddler and infant cohort).

- All toddlers and infants in the study will receive an additional SC dose of a measles and rubella vaccine so will not miss out. Adults are not routinely included in measles and rubella vaccination campaigns but will be given any missed doses under these circumstances.

- Be willing to avoid all vaccines not given by the trial team for the duration of the study with the exception of non-measles and rubella vaccines given in national campaigns

- Have a parent who is willing to ensure they avoid all vaccines not given by the trial team for the duration of the study with the exception of non-measles and rubella vaccines given in national campaigns (toddler and infant cohort).

Exclusion criteria

A prospective participant will not be eligible for enrolment if they meet any of the following exclusion criteria.

Participants <u>must not</u>:

- Have used any investigational product within the 90 days prior to study product administration or plan to use any investigational products during the period of study participation.

- Have consumed (by ingestion or topical application) any herbal or other traditional medication within 14 days of study product administration.
- Have a history of serious reactions to any prior vaccination or known hypersensitivity to any component of the MRV-MNP, MRV-SC or PLA-MNP including polyethylene foam with acrylic adhesive, silicone-coated Kraft paper, stainless steel, and severe allergic reactions to cow's milk.
- Have a history of anaphylactic shock or other life-threatening allergic reactions
- Have any chronic, clinically significant pulmonary, cardiovascular, hepatobiliary, gastrointestinal, renal, neurological, or haematological abnormality or illness that requires medical therapy, as determined by medical history, physical examination and laboratory assessment.
- Have a history of administration of any non-study vaccines within the 56 days before the administration of study products or planned vaccination during study participation, except for non-measles and rubella catch-up/national campaign administered through the Gambian Ministry of Health.
- Have a history of chronic administration (defined as more than 14 consecutive days) of immunosuppressant (> 0.5mg/kg/day of prednisolone or equivalent) or other immune modifying drugs within the 12 months prior to the administration of the study vaccine including the use of glucocorticoids. The use of inhaled/per nasal glucocorticoids will be permitted. The use of topical glucocorticoids within 12 months is not permitted (specific enquiry regarding the use of skin lightening creams should be made).
- Have a history of the administration of immunoglobulins and/or any blood products within the 12 months prior to administration of the study vaccine or anticipation of such administration during the study period.
- Have a history of known disturbance of coagulation or blood disorder that could cause anaemia or excess bleeding (e.g., sickle cell disorders, thalassemia, and coagulation factor deficiencies).
- Have a history of keloid formation.
- Have significant scars, tattoos, rashes, or other dermatologic condition in the area of the vaccination site which will interfere with the application of the MNP and assessment of local solicited AE.
- Have human immunodeficiency virus (HIV), hepatitis B or hepatitis C infection based on screening laboratory investigations.

- Have any medical or social condition that in the opinion of the study clinician may interfere with the study objectives, pose a risk to the participant, or prevent the participant from completing the study follow-up.

- Be an employee of, or direct descendant (child or grandchild) of any person employed by the investigator or sponsor.

- Have plans to travel outside the study area for an extended duration during the period of study participation.

- This is particularly critical in the first 42 days following study product administration during which any travel will be actively discouraged. At later time-points, short trips within The Gambia can generally be accommodated.

- Have any screening laboratory test (full blood count including differential white cell count; sodium, potassium, urea, creatinine, albumin, total protein, alanine transaminase, aspartate transaminase, gamma glutamyl transferase, calcium) with a toxicity score of ≥ 2 or with a toxicity score of 1 which is nonetheless judged to be clinically significant by the trial clinician. If judged to be clinically indicated, each laboratory assessment may be repeated once during the screening period, with the most recent laboratory value being used for evaluation of exclusion criteria. However, abnormal laboratory investigations will not 'routinely' be repeated unless there is a clinical indication as to why the initial result was abnormal and it considered likely that the abnormality will have resolved.

- Have any vital sign (heart rate, respiratory rate, non-invasive blood pressure [adult cohort only]) with a toxicity score of > 1. An abnormal vital sign may be repeated once during the screening period for a participant to remain eligible for randomization with the most recent set of vital signs being used to determine final eligibility§.

- Have an axillary temperature of > 37.5°C and have had a documented fever at the same level in the 72 hours preceding randomization and vaccination§.

- Have a history of an illness with a fever and rash suggestive of measles in the preceding two months.

- Have any acute illness (severity grade > 2) §.

- Have a positive rapid diagnostic test (RDT) (or blood film) for malaria. If a participant initially has a positive RDT and is then treated, a blood film will be undertaken if the RDT remains positive, to confirm treatment success given an RDT may remain positive even following successful treatment§
- Adult cohort only: Have been vaccinated against measles or rubella in the preceding four-years.
- Adult cohort only: Have a BMI of < 18.5kg/m2 (underweight) or > 35kg/m2 (severely obese).
- Adult cohort only: Have a recent history (within the past year) or signs of alcohol or substance abuse.
- Adult cohort only: Have a history of major psychiatric disorder.
- Adult cohort only: Have a history of blood donation within three months of study enrolment or plans to donate blood during participation in the study.
- Adult female cohort only: Be pregnant or breast-feeding.
- Toddler and infant cohort only: Have been vertically exposed to HIV based on maternal history (mothers of potential participants will not be tested for HIV as part of screening).
- Toddler and infant cohorts only: Have a weight for height z-score below -2SD (moderate malnutrition).
- Infant cohort only: Have been vaccinated against measles or rubella.

§Participants with an acute illness, fever, other abnormal vital signs or a positive malaria RDT test may return once for a repeat screening visit within the 2-week screening period and still qualify for randomization if the acute illness has resolved. A minimum of 72 hours following a documented fever (axillary temperature ≥ 37.5°C) must pass before a participant can be re-screened and vaccinated. In general, illnesses lasting more than the 2-week screening window will be considered to define a potential participant as a screen failure although a participant providing ongoing informed consent could be fully rescreened under such circumstances in the absence of any other reasons to define them as such.

Specific exclusion criteria (vital signs, clinical examination, history of acute illness, blood test for malaria, urinary pregnancy test [adult female cohort only]) will be reassessed at the time of V1 and prior to confirming final eligibility and proceeding to randomization to ensure only those participants appropriate for vaccination on the day are included in the study.

Study schedule.

Adults

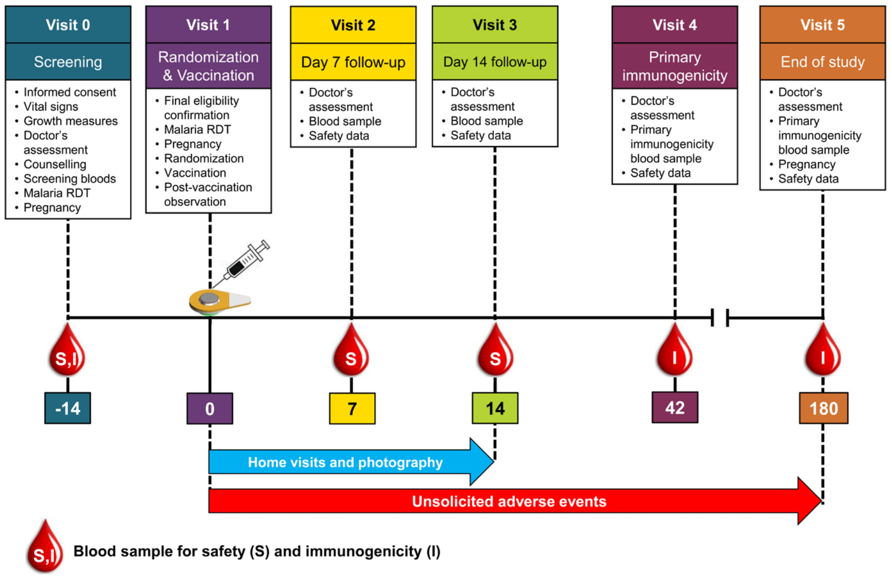

Toddlers and infants

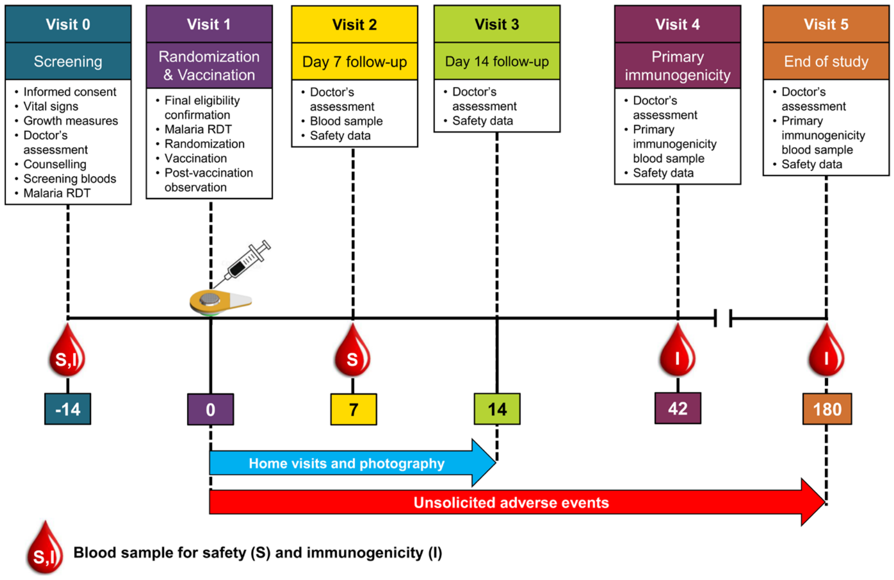

Additional immunizations administered to participants by age cohort.

Cohort	Age/timepoint	Vaccine	Route
**Adults**	After day 42 blood sample	COVID-19 vaccine (according to participant choice)	Intramuscular
**Toddlers**	After day 42 blood sample	Diphtheria-tetanus-pertussis (DTP)	Intramuscular
		Bivalent oral poliovirus vaccine (bOPV)	Oral
	After day 180 blood sample	Measles and rubella vaccine (MRV)	Subcutaneous
**Infants**	After day 42 blood sample	Yellow fever vaccine	Subcutaneous
		Bivalent oral poliovirus vaccine (bOPV)	Oral
	At 12 months of age	Meningococcus serogroup A conjugate vaccine (MenAfriVac™)	Intramuscular
	After day 180 blood sample	Measles and rubella vaccine (MRV)	Subcutaneous

Local and systemic adverse event grading

Adult cohort: local solicited adverse event grading [1]

Local administration site	Grade 0	Grade 1<br>Mild	Grade 2<br>Moderate	Grade 3<br>Severe	Grade 4§<br>Potentially Life Threatening
Pain	No pain	Pain causing no or minimal limitation to use of limb	Pain causing greater than minimal limitation to use of limb	Pain causing inability to perform usual social and functional activities	Pain causing inability to perform basic self-care function or hospitalization indicated
Redness/Erythema‡<br>(size as well as grade will be collected)	< 2.5cm in diameter	2.5cm to < 5cm in diameter OR 6.25 to <25 cm^2 surface area AND symptoms causing no or minimal interference with usual social and functional activities	≥ 5cm to < 10 cm in diameter OR ≥ 25 to <100 cm^2 surface area OR symptoms causing greater than minimal interference with usual social and functional activities	≥ 10 cm in diameter OR ≥ 100 cm^2 surface area OR ulceration OR secondary infection OR phlebitis OR sterile abscess OR drainage OR symptoms causing inability to perform usual social and functional activities	Potentially life-threatening consequences (e.g. abscess, exfoliative dermatitis, necrosis involving dermis or deeper tissues)
Swelling/induration‡<br>(size as well as grade will be collected)	< 2.5cm in diameter	2.5cm to < 5cm in diameter OR 6.25 to <25 cm^2 surface area AND symptoms causing no or minimal interference with usual social and functional activities	≥ 5cm to < 10 cm in diameter OR ≥ 25 to <100 cm^2 surface area OR symptoms causing greater than minimal interference with usual social and functional activities	≥ 10 cm in diameter OR ≥ 100 cm^2 surface area OR ulceration OR secondary infection OR phlebitis OR sterile abscess OR drainage OR symptoms causing inability to perform usual social and functional activities	Potentially life-threatening consequences (e.g. abscess, exfoliative dermatitis, necrosis involving dermis or deeper tissues)
Pruritis (localized to injection site	No pruritis at injection site	Itching localized to injection site AND no or minimal limitation to function	Itching beyond injection site that is not generalized OR itching that causes more than minimal loss of function	Generalized itching that causes inability to perform usual social and functional activities	N/A

§Any AE resulting in death will be defined as grade 5 severity. ‡Grading based on the greatest single diameter or measured surface area

[1]Based on the National Institute of Health, Division of AIDS (DAIDS) Table for Grading the Severity of Adult and Pediatric AEs – Corrected Version 2.1 July 2017

Adult cohort: systemic solicited adverse event grading [2]

Systemic	Grade 0	Grade 1 Mild	Grade 2 Moderate	Grade 3 Severe	Grade 4§ Potentially Life Threatening
Acute allergic reactions‡	No acute allergic reaction	Localized urticaria (wheals) with no intervention indicated†	Localized urticaria (wheals) with intervention indicated OR mild angioedema with no intervention indicated†	Generalized urticaria OR angioedema with intervention indicated OR symptoms of mild bronchospasm (wheeze)	Anaphylaxis OR life-threatening bronchospasm (wheeze) or laryngeal oedema (stridor)
Axillary Temperature	< 37.5°C	37.5 to 38.4˚C	38.5 – 38.9˚C	39.0 – 40.0˚C	> 40˚C
Vomiting	No vomiting	Transient or intermittent AND no or minimal interference with oral intake	Frequent episodes with no or mild dehydration – oral rehydration solution indicated	Persistent vomiting resulting in orthostatic hypotension OR aggressive rehydration indicated (e.g. intravenous fluids)	Life threatening consequences (e.g. hypotensive shock)
Diarrhoea	No diarrhoea	Transient or intermittent episodes of unformed stool OR increase of ≤ 3 stools over baseline per 24-hour period	Persistent episodes of unformed watery stool OR increase of 4 to 6 stools over baseline per 24-hour period	Increase of ≥ 7 stools per 24-hour period OR intravenous fluid replacement indicated	Life threatening consequences (e.g. hypotensive shock)
Headache	No headache	Symptoms causing no or minimal interference with usual social and functional activities	Symptoms causing greater than minimal interference with usual social and functional activities	Symptoms causing inability to perform usual social and functional activities	Symptoms causing inability to perform basic self-care functions OR hospitalization indicated OR headache with significant impairment of alertness or other neurological function
Fatigue	No fatigue	Fatigue causing no or minimal interference with usual social and functional activities	Fatigue causing greater than minimal interference with usual social and functional activities	Fatigue causing inability to perform usual social and functional activities	Incapacitating symptoms of fatigue causing inability to preform basic self-care functions
Myalgia	No muscle pain	Muscle pain causing no or minimal interference with	Muscle pain causing greater than minimal interference	Muscle pain causing inability to perform	Incapacitating muscle pain causing inability to preform

[2] Based on the National Institute of Health, Division of AIDS Table for grading the severity of adult and pediatric AE – Version 2.1 July 2017

**Systemic**	**Grade 0**	**Grade 1**<br>**Mild**	**Grade 2**<br>**Moderate**	**Grade 3**<br>**Severe**	**Grade 4§**<br>**Potentially Life Threatening**
		usual social and functional activities	with usual social and functional activities	usual social and functional activities	basic self-care functions
Arthralgia	No joint pain	Join pain causing no or minimal interference with usual social and functional activities	Joint pain causing greater than minimal interference with usual social and functional activities	Joint pain causing inability to perform usual social and functional activities	Incapacitating joint pain causing inability to preform basic self-care functions
Rash at a site other than product administration site	No rash	Localized rash at a site other than the product administration site.	Diffuse rash OR target lesions	Diffuse rash AND vesicles or limited number of bullae or superficial ulceration of mucous membranes at one site	Extensive of generalized bullous lesions OR ulceration of mucous membranes involving tow or more distinct mucosal sites OR Stevens-Johnson syndrome OR toxic epidermal necrolysis

For all solicited systemic AE the terminology used in local languages will be standardized in advance and age appropriate social and functional activities will be defined.

§Any AE resulting in death will be defined as grade 5 severity.

‡Collected only on the day of vaccination itself (60 +/- 15 minutes post-vaccination). Not collected during day 1 to 13 home visits

†Localized in this case does not need to reflect urticaria localized to the site of the injection itself but rather urticarial localized to one anatomical location

Toddler and infant cohort: local solicited adverse event grading[3]

**Local administration site**	**Grade 0**	**Grade 1 Mild**	**Grade 2 Moderate**	**Grade 3 Severe**	**Grade 4§ Potentially Life Threatening**
Pain	No pain	Pain causing no or minimal limitation to use of limb	Pain causing greater than minimal limitation to use of limb	Pain causing inability to perform usual social and functional activities	Pain causing inability to perform basic self-care function or hospitalization indicated
Redness/Erythema‡<br>(size as well as grade will be collected)	No redness/ erythema	≤ 2.5cm in diameter	> 2.5cm in diameter with < 50% of the surface area of the extremity segment involved (e.g. lower arm or thigh)	≥ 50% of the surface area of the extremity segment involved (e.g. lower arm or thigh) OR ulceration OR secondary infection OR phlebitis OR sterile abscess OR drainage	Potentially life-threatening consequences (e.g. abscess, exfoliative dermatitis, necrosis involving dermis or deeper tissues)
Swelling/induration‡<br>(size as well as grade will be collected)	No swelling/ induration	≤ 2.5cm in diameter	> 2.5cm in diameter with < 50% of the surface area of the extremity segment involved (e.g. lower arm or thigh)	≥ 50% of the surface area of the extremity segment involved (e.g. lower arm or thigh) OR ulceration OR secondary infection OR phlebitis OR sterile abscess OR drainage	Potentially life-threatening consequences (e.g. abscess, exfoliative dermatitis, necrosis involving dermis or deeper tissues)

§Any AE resulting in death will be defined as grade 5 severity; ‡Grading based on the greatest single diameter or measured surface area

[3] Based on the National Institute of Health, Division of AIDS (DAIDS) Table for Grading the Severity of Adult and Pediatric Adverse Events – Corrected Version 2.1 July 2017

Toddler and infant cohort: systemic solicited adverse event grading [4]

**Systemic**	**Grade 0**	**Grade 1**<br>**Mild**	**Grade 2**<br>**Moderate**	**Grade 3**<br>**Severe**	**Grade 4§**<br>**Potentially Life Threatening**
Acute allergic reactions‡	No acute allergic reaction	Localized urticaria (wheals) with no intervention indicated†	Localized urticaria (wheals) with intervention indicated OR mild angioedema with no intervention indicated†	Generalized urticaria OR angioedema with intervention indicated OR symptoms of mild bronchospasm (wheeze)	Anaphylaxis OR life-threatening bronchospasm (wheeze) or laryngeal oedema (stridor)
Axillary Temperature	< 37.5°C	37.5 to 38.4˚C	38.5 – 38.9˚C	39.0 – 40.0˚C	> 40˚C
Vomiting	No vomiting	Transient or intermittent AND no or minimal interference with oral intake	Frequent episodes with no or mild dehydration – oral rehydration solution indicated	Persistent vomiting resulting in orthostatic hypotension OR aggressive rehydration indicated (e.g. intravenous fluids)	Life threatening consequences (e.g. hypotensive shock)
Diarrhoea	No diarrhoea	Liquid stools (less formed than usual) but usual number of stools	Liquid stools with increased number of stools OR mild dehydration	Liquid stool with moderate dehydration	Life threatening consequences (e.g. liquid stool resulting in severe dehydration, hypotensive shock)
Irritability	No irritability	Crying more than normal/irritability but no or minimal interference with usual social and functional activities	Crying more than normal/irritability causing greater than minimal interference with usual social and functional activities	Crying more than normal/irritability preventing usual social and functional activities	Requiring hospitalization due to irritability
Drowsiness	No drowsiness	Sleeping more than normal/drowsiness but no or minimal interference with usual social and functional activities	Sleeping more than normal/drowsiness causing greater than minimal interference with usual social and functional activities	Sleeping more than normal/drowsiness preventing usual social and functional activities	Requiring hospitalization due to drowsiness

[4] Modified from the National Institute of Health, Division of AIDS Table for grading the severity of adult and pediatric AE – Version 2.1 July 2017; US Department of Health and Human Services, Food and Drug Administration, Guidance for Industry, Toxicity Grading Scale for Healthy Adult and Adolescent Volunteers Enrolled in Preventative Vaccine Clinical Trials, Sep 2007.

**Systemic**	**Grade 0**	**Grade 1 Mild**	**Grade 2 Moderate**	**Grade 3 Severe**	**Grade 4§ Potentially Life Threatening**
Appetite	Eating/ feeding normally	Eating/feeding less than normal but no or minimal interference with usual social and functional activities	Eating/feeding less than normal with greater than minimal interference with usual social and functional activities	Eating/feeding less than normal preventing usual social and functional activities	Requiring hospitalization due to not eating/feeding.
Rash at a site other than product administration site	No rash	Localized rash at a site other than the product administration site.	Diffuse rash OR target lesions	Diffuse rash AND vesicles or limited number of bullae or superficial ulceration of mucous membranes at one site	Extensive of generalized bullous lesions OR ulceration of mucous membranes involving tow or more distinct mucosal sites OR Stevens-Johnson syndrome OR toxic epidermal necrolysis

For all solicited systemic AE the terminology used in local languages will be standardized in advance and age appropriate social and functional activities will be defined; §Any AE resulting in death will be defined as grade 5 severity; ‡Collected only on the day of vaccination itself (60 +/- 15 minutes post-vaccination). Not collected during day 1 to 13 home visits; †Localized in this case does not need to reflect urticaria localized to the site of the injection itself but rather urticarial localized to one anatomical location

Severity grading for unsolicited adverse events [5]

All other AE will be graded for severity according to the criteria set out in the Division of AIDS (DAIDS) Table for Grading the Severity of Adult and Paediatric AEs (V2.1 July 2017) or if not included in in these tables, as below:

		Description
Grade 1	Mild	No interference with activity and no or minimal intervention[1] required
Grade 2	Moderate	Some interference with activity or requires more than minimal intervention
Grade 3	Severe	Prevents daily activity and required significant medical intervention
Grade 4	Life-threatening	Life threatening consequences requiring urgent medical intervention
Grade 5	Death	Results in death

[1]e.g. one or two doses of antipyretic or simple analgesic medication or local topical treatment.

[5] Adapted from Cancer Therapy Evaluation Program, Common Terminology Criteria for AEs, Version 3.0, DCTD, NCI, NIH, DHHS March 31 2003, published August 9 2006

CDC Statement:

This study was reviewed by CDC and was conducted consistent with applicable federal law and CDC policy. See e.g., 45 C.F.R. part 46; 21 C.F.R. part 56; 42 U.S.C. §241(d), 5 U.S.C. §552a, 44 U.S.C. §3501 et seq.

International standards used to calibrate measles and rubella serological results.

Measles:

WHO International Standard, 3rd International Standard for Anti-Measles. National Institute of Biological Standards and Control Code 97/648.[1]

Rubella:

WHO International Standard, Anti-Rubella Immunoglobulin, Human.[2]

Unsolicited adverse event relatedness assessment

Other than solicited local reactions which, by definition, are related to vaccination, other adverse events including solicited systemic reactions, abnormal clinical laboratory parameters and vital signs were assessed for relatedness to the study vaccine by a study clinician.

The relatedness of a particular adverse was assessed based on clinical judgment considering the timing of the event in relation to study product administration, the nature of the event, the presence or absence of other illnesses or conditions to explain the event and relevant background history and concomitant medication use.

In the absence of any other apparent illness (e.g. malaria causing a fever), any solicited systemic adverse event documented on day 0 and during the day 1 to 13 home visits was generally defined as being related to vaccine administration. However, the timing following study product administration was also considered as the fever and rash occurring in a small number of measles containing vaccine recipients typically occurs between 7- and 12-days following vaccination

Based on these assessments, the relationship between a given adverse event and study product was defined as:

- Related[6]: There is a reasonable possibility of a causal relationship between the adverse event and the study product administered. The adverse event is more likely to be explained by the administration of the study product than by another cause. Related adverse events are, by definition, adverse reactions.
- Not related: There is not a reasonable possibility of a causal relationship between the adverse event and the study product administered. The adverse event is more likely to be explained by another cause.

[6] Definition based on the revised European Commission 'Detailed guidance on the collection, verification and presentation of AE/reaction reports arising from clinical trials of medicinal products for human use'.

Sample size considerations.

The sample size was not determined by a power calculation based on testing a formal statistical hypothesis. Instead, it has been chosen to provide the required descriptive data on the safety and tolerability of the measles and rubella MNP to guide decisions regarding product development and to provide supporting information regarding the immunogenicity of the measles and rubella MNP.

Safety and tolerability

Table 1 and Figure 1 illustrate the probability that a given safety event will occur at least once or at least twice based on true event rates in the vaccinated population of between 1% and 20% based on the different sample sizes (n).

A sample size of 30 participants provides a probability of 95.8% that at least one episode of a given safety event will occur and a probability of 81.6% that at least two episodes of an event will occur based on a true event rate of 10% in the vaccinated population. The same sample size provides probability of 78.5%, 53.2% and 26.0% that at least one event will occur given a true event rate of 5%, 2.5% and 1% in the population. If a given safety event does not occur in 30 adult participants, we can be 95% confident that the true event rate in the population is less than 11.4%.

A sample size of 60 participants provides a probability of 99.8% that at least one episode of a given safety event will occur and a probability of 98.6% that at least two episodes of an event will occur in each of the given cohorts based on a true event rate of 10% in the vaccinated cohort. The same sample size provides probability of 95.4%, 78.1% and 45.3% that at least one event will occur given a true event rate of 5%, 2.5% and 1% in the cohort. If a given safety event does not occur in a cohort, we can be 95% confident that the true event rate in that cohort is less than 6.0%.

A sample size of 150 participants receiving the measles and rubella MNP overall provides a probability of close to 100.0% that at least two episodes of an event will occur in the whole study population based on a true event rate of 10% in the vaccinated cohort. The same sample size provides probability of close to 100.0%, 97.8% and 79.9% that at least one event will occur given a true event rate of 5%, 2.5% and 1% in the cohort. If a given safety event does not occur in a study, we can be 95% confident that the true event rate in that cohort is less than 2.5%.

Table 2 and Figure 2 indicate the expected precision surrounding the estimates of given safety event rates in each cohort and in all participants who will receive the measles and rubella MNP during the study. For example, if a fever (or any other event) is recorded in 5% of all participants who receive the measles and rubella MNP we will be 95% confident that the true frequency of fever related to measles and rubella MNP administration lies between 2.5% and 9.8%.

Finally, Figure 3 illustrates the expected precision surrounding the estimates of the absolute differences in given safety event rates between those receiving the measles and rubella MNP and those receiving the measles and rubella vaccine SC. For example, if the rate of a given safety event is 1% in the measles and rubella vaccine SC group and 4% (absolute difference 3%) in the measles and rubella MNP group, the 95% CI of the difference between the two groups is expected to exclude 0 (i.e., no difference) (Figure 3 A). Similarly, if the rate of a given safety event is 10% in the measles and rubella vaccine SC group and 18% (absolute difference 8%) in the measles and rubella MNP group, the 95% CI of the difference between the two groups is expected to exclude 0 (i.e., no difference) (Figure 3 C).

Table 1: The probability of any given safety event occurring at least once or at least twice given a true population event rate of between 1.0% and 20.0% according to the indicated samples sizes per group (also illustrated in Figure 1)

True event rate (%)	n = 30		n = 60		n = 150	
	Probability of at least 1 event	Probability of at least 2 events	Probability of at least 1 event	Probability of at least 2 events	Probability of at least 1 event	Probability of at least 2 events
**20**	99.9	98.9	100.0	100.0	100.0	100.0
**10**	95.8	81.6	99.8	98.6	100.0	100.0
**7.5**	90.4	66.9	99.1	94.5	100.0	100.0
**5**	78.5	44.6	95.4	80.8	100.0	99.6
**2.5**	53.2	17.2	78.1	44.4	97.8	89.1
**1**	26.0	3.6	45.3	12.1	77.9	44.3

Figure 1: The probability of a given safety event occurring at least once or at least twice given a true population event rate of between 1.0% and 10.0% according to the indicated samples sizes per group.

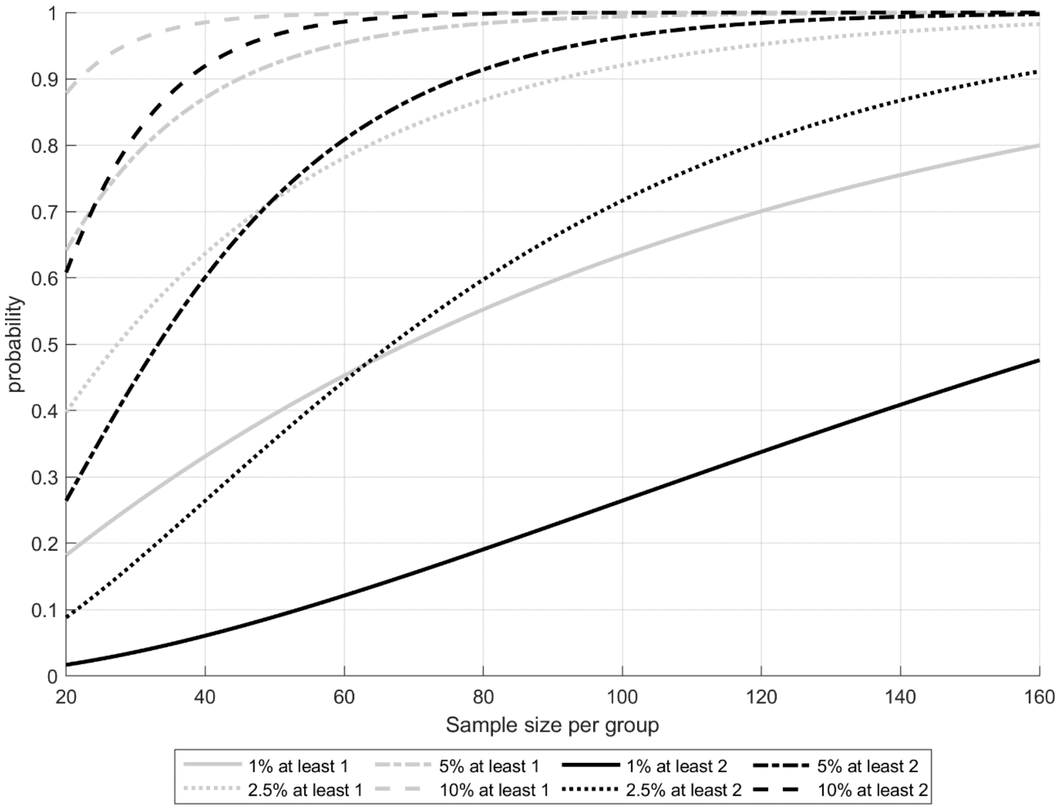

Figure 2: Graphs illustrating the expected precision around given safety event rate estimates in adults, toddlers/infants and the whole measles and rubella MNP population.

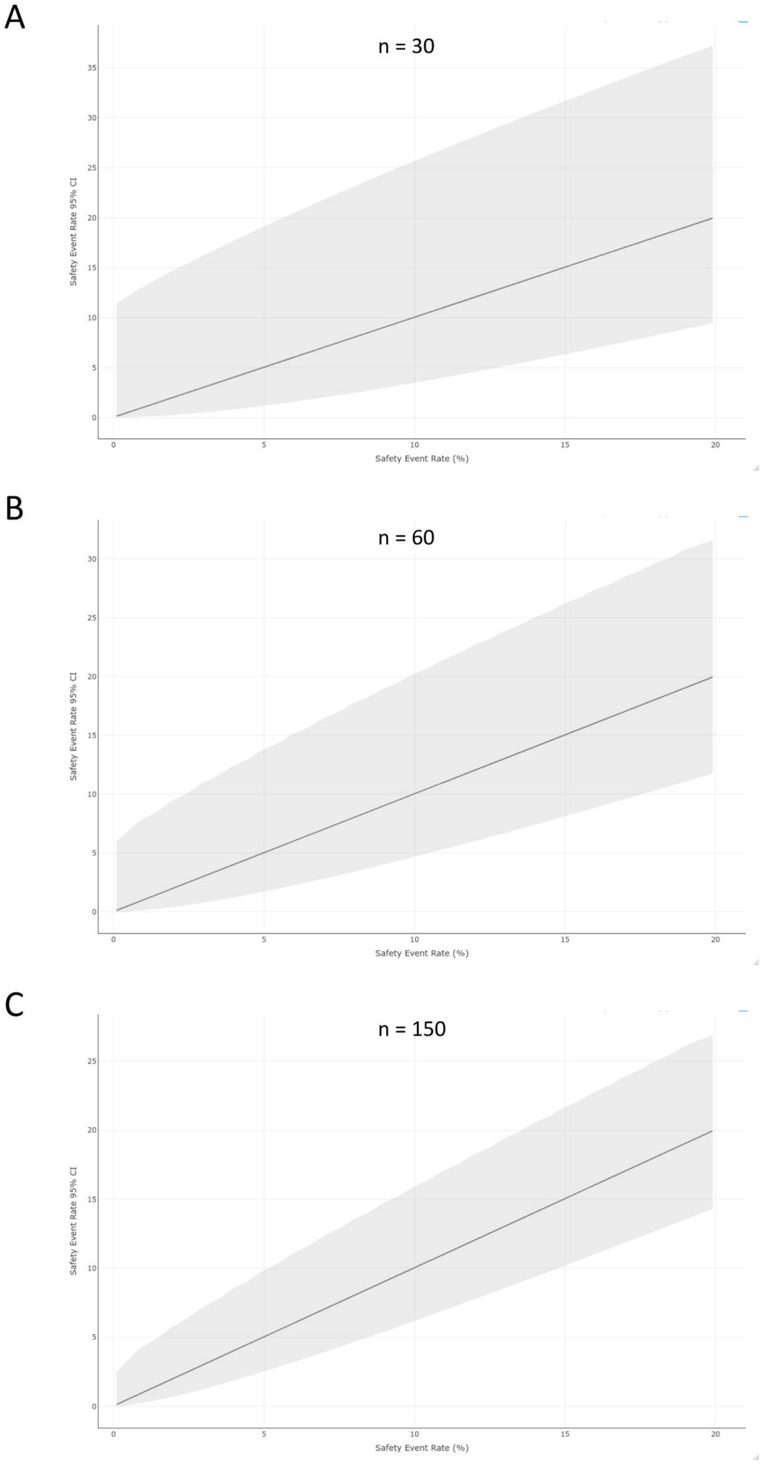

Table 2: Expected precision around the given safety event rate estimates in adults, toddler/infants and the whole population who will receive the measles and rubella MNP (also illustrated in Table 2)

Event rate in sample (%)	n = 30	n = 60	n = 150
	Event Rate (%) (95% CI)	Event rate (95% CI)	Event rate (95% CI)
**20.0**	20.0 (9.5 – 37.3)	20 (11.8 – 31.8)	20.0 (14.4 – 27.1)
**10.0**	10.0 (3.5 – 25.6)	10 (4.7 – 20.2)	10.0 (6.2 – 15.8)
**7.5**	7.5 (22 – 22.4)	7.5 (3.1 – 17.0)	7.5 (4.3 – 12.9)
**5.0**	5.0 (1.2 – 19.1)	5.0 (1.7 – 13.7)	5.0 (2.5 – 9.8)
**2.5**	2.5 (0.3 – 15.4)	2.5 (0.6 – 10.1)	2.5 (1.0 – 6.4)
**1.0**	1.0 (0.0 – 13.1)	1.0 (0.1 – 7.8)	1.0 (0.2 – 4.2)

Figure 3: Graphs illustrating the expected precision around absolute differences in safety event rate estimates between the measles and rubella MNP group (n = 150) and the measles and rubella vaccine SC group (n = 135). (A) 1%, (B) 5%, (C) 10%, (D) 20%.

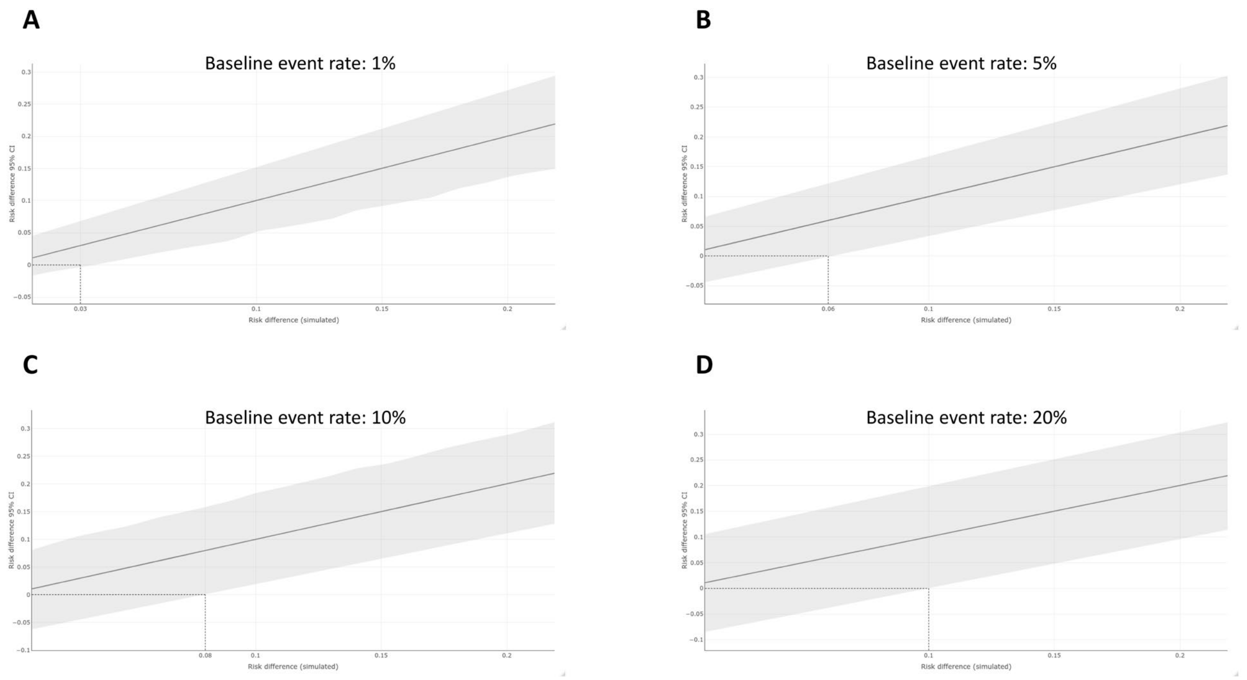

Immunogenicity

Table 3 and Figure 4 indicate the expected precision surrounding the estimates of given immune response rates in each cohort. For example, if the immune response rate in adults vaccinated with measles and rubella MNP is 60% we will be 95% confident that the true immune response rate in adults lies between 42.3% and 75.4%. Similarly, if the immune response rate in infants is 80%, we will be 95% confident that the true immune response rate in infants lies between 68.2% and 88.2%.

Finally, Figure 4 illustrates the expected precision surrounding the estimates of the absolute differences in immune response rate between those receiving the measles and rubella MNP and those receiving the measles and rubella vaccine SC based on a hypothetical immune response rate in the latter group of 80%. In adults, the 95% CI around the difference in immune response rates would be expected to exclude 0 (i.e., no difference) if the immune response rate in the measles and rubella MNP was below 53% (Figure 4 A). In toddlers and infants, the 95% CI around the difference in immune response rates would be expected to exclude 0 (i.e., no difference) if the immune response rate in the measles and rubella MNP group is below 65% (Figure 4 B).

Figure 4: Graphs illustrating the expected precision around given immune response rate estimates in (A) adults and (B) toddlers/infants.

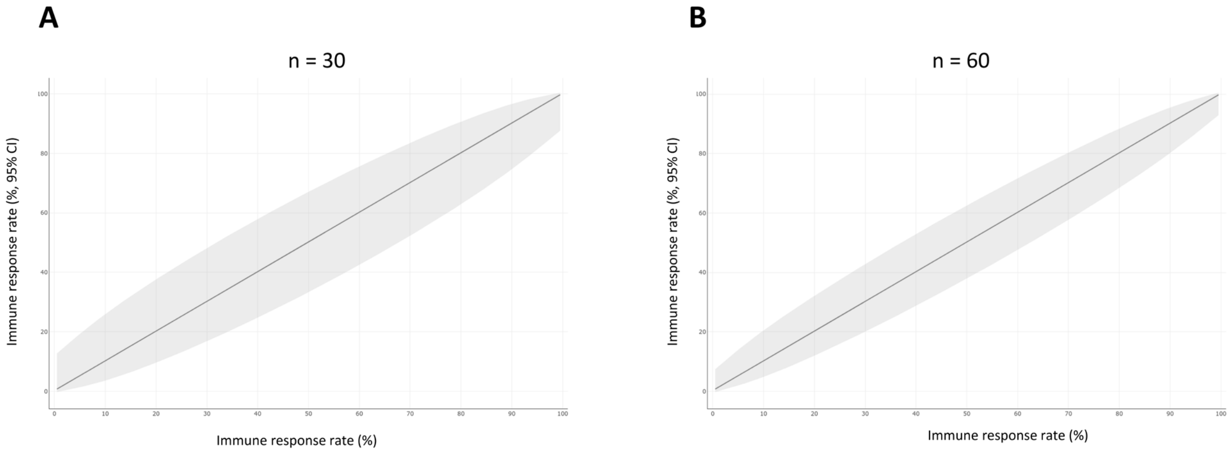

Table 3: Expected precision around the given immune response rate estimates in adults and toddler/infants who will receive the measles and rubella MNP (also illustrated in Figure 4)

Immune response rate in sample (%)	n = 30	n = 60
	Immune response (%) (95% CI)	Immune response (95% CI)
**20.0**	20.0 (9.5 – 37.3)	20.0 (11.8 – 31.8)
**40.0**	40.0 (24.6 – 57.7)	40.0 (28.6 – 52.6)
**60.0**	60.0 (42.3 – 75.4)	60.0 (47.4 – 71.4)
**80.0**	80.0 (62.7 – 90.5)	80.0 (68.2 – 88.2)
**100.0**	100.0 (88.7 – 100.0)	100.0 (94.0 – 100.0)

Figure 5: Graph illustrating the precision around absolute differences in immune response rate estimates in adults (A) and toddlers/infants (B)

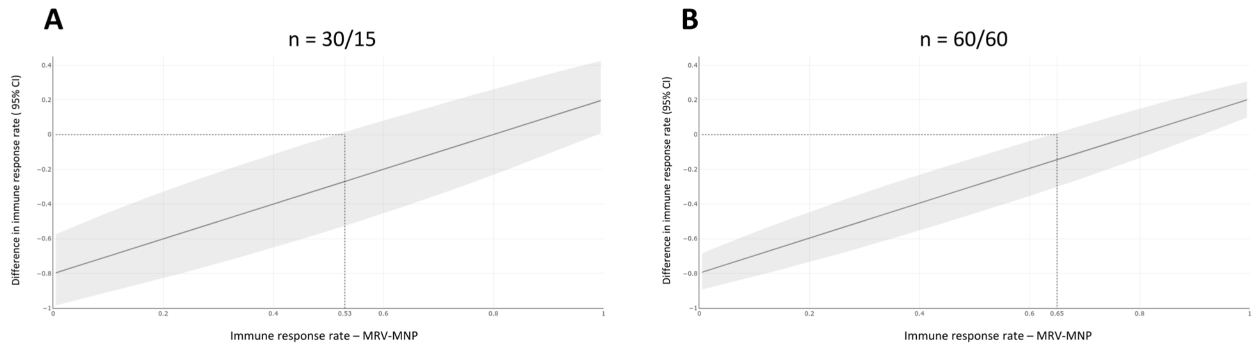

Analysis populations and missing data

Safety population

The safety population will consist of all vaccinated participants. Analysis will be conducted according to route of MRV administration (i.e., MRV-MNP or MRV-SC) and will include all participants irrespective of any subsequent protocol deviations. All safety data collected up to the point of discontinuation, if applicable, will be included in the analyses.

All baseline data (demographics, anthropometrics, medical history, vital signs, clinical laboratory examinations) will be presented for the safety population and may additionally be presented for given immunogenicity populations.

Immunogenicity populations

Primary immunogenicity population

The primary immunogenicity population will consist of all participants vaccinated, who have a baseline and a visit 4 (day 42) immunogenicity result available. Analysis will be conducted according to route of MRV administration (i.e., MRV-MNP or MRV-SC) and will include all participants irrespective of any subsequent protocol deviations.

Analysis will be conducted separately for measles and rubella SNA and IgG therefore four separate populations could be defined if any of the serological results are not available.

A confirmatory per protocol analysis will be conducted which will exclude any participants who experienced a protocol deviation considered on blinded review to potentially interfere with the given immunogenicity endpoint.

Secondary immunogenicity population

Secondary immunogenicity populations will consist of all participants vaccinated who have a serological result available at a given timepoint. Analysis will be conducted according to route of MRV administration (i.e., MRV-MNP or MRV-SC) and will include all participants irrespective of any subsequent protocol deviations.

Analysis will be conducted separately for measles and rubella SNA and IgG therefore four separate populations could be defined if any of the serological results are not available.

A confirmatory per protocol analysis will be conducted which will exclude any participants who experienced a protocol deviation considered on blinded review to potentially interfere with the given immunogenicity endpoint.

A confirmatory per protocol population which excluded participants with a protocol deviation considered on blinded review to potentially interfere with the immunogenicity endpoints. The primary and per protocol immunogenicity populations were identical so are not reported separately.

Any missing data were considered missing completely at random. No data were imputed.

Supplementary Figure: Trial profile - adult cohort

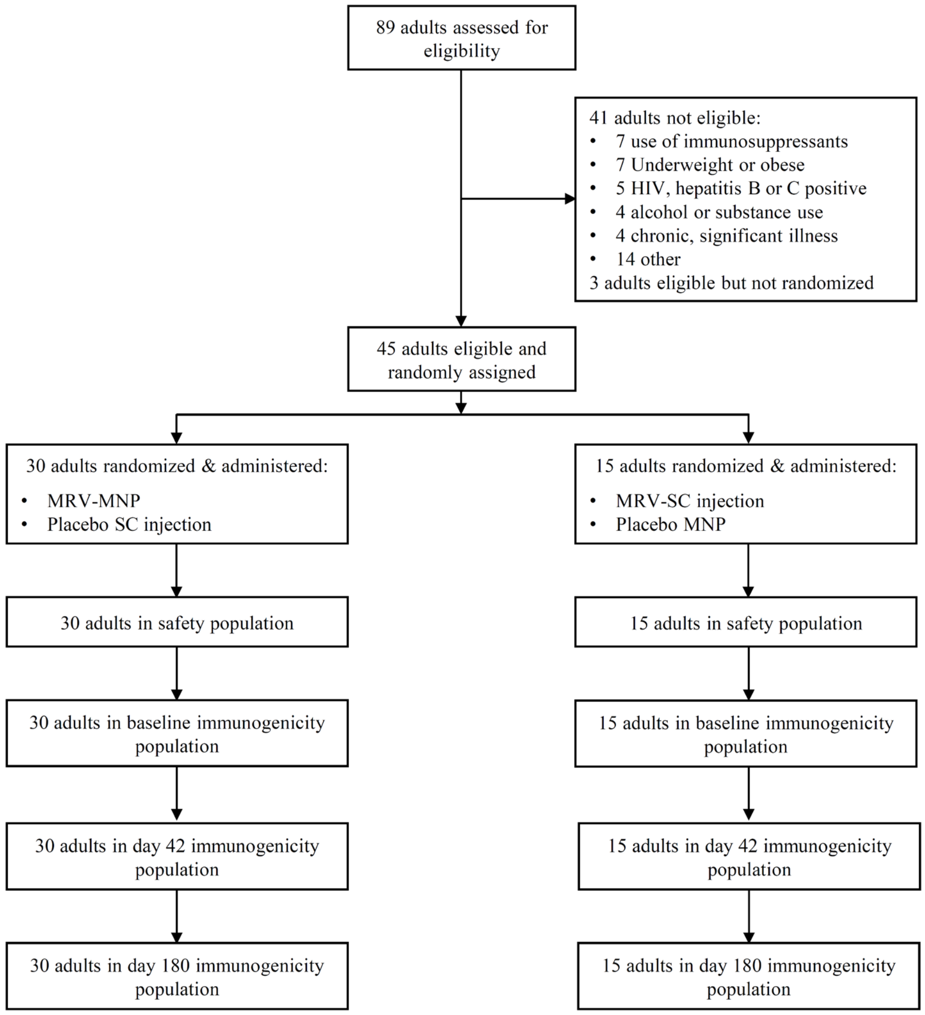

HIV – human immunodeficiency virus; MNP – microneedle patch; SC – subcutaneous;

Supplementary Table: Demographic and baseline anthropometric data – adult cohort

	Adults	
	**MRV-MNP & placebo SC**	**MRV-SC & placebo MNP**
	**N = 30**	**N = 15**
**Age (Years)§**		
- Median (Q1 to Q3)	22 (20 to 30)	21 (20 to 24)
**Sex, n (%)**		
- Male	25 (83.3)	13 (86.7)
- Female	5 (16.7)	2 (13.3)
**Race, n (%)**		
- African	30 (100)	15 (100)
**Tribe, n (%)**		
- Mandinka	15 (50.0)	9 (60.0)
- Wolof	2 (6.7)	2 (13.3)
- Fula	5 (16.7)	1 (6.7)
- Jola	3 (10.0)	2 (13.3)
- Other	5 (16.7)	1 (6.7)
**Weight (kg)‡**		
- Median (Q1 to Q3)	68.9 (63.9 to 76.9)	65.0 (57.2 to 71.0)
**Height (cm)‡**		
- Median (Q1 to Q3)	174.3 (166.8 - 178.8)	177.0 (174.8 - 181.8)
**Body mass index (kg/m^2)‡**		
- Median (Q1 to Q3)	22.1 (20.4 to 25.3)	20.0 (19.5 to 21.4)

Q1 – quartile 1; Q3 – quartile 3; MNP – microneedle patch; SC – subcutaneous; N – number of participants included; kg – kilogrammes; cm – centimetres; kg/m^2 – kilogrammes per metre squared; § on the day of consent; ‡on the day of randomization and vaccination.

Supplementary Figure: Safety laboratory parameters

Adult cohort

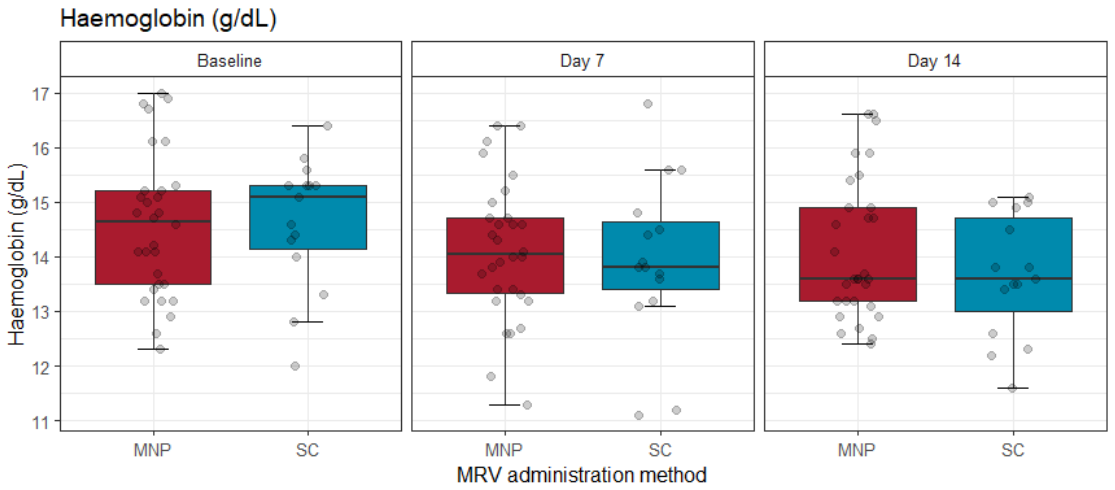

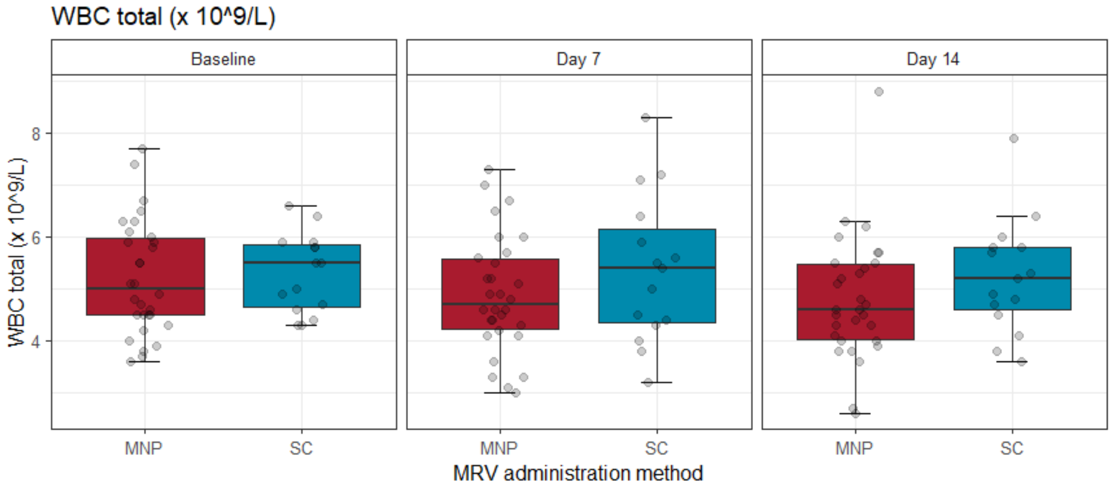

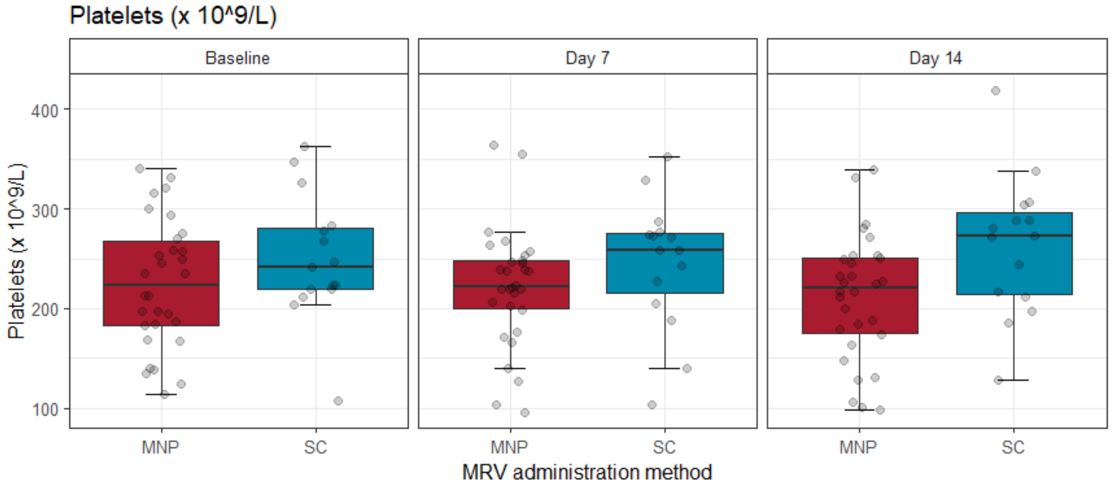

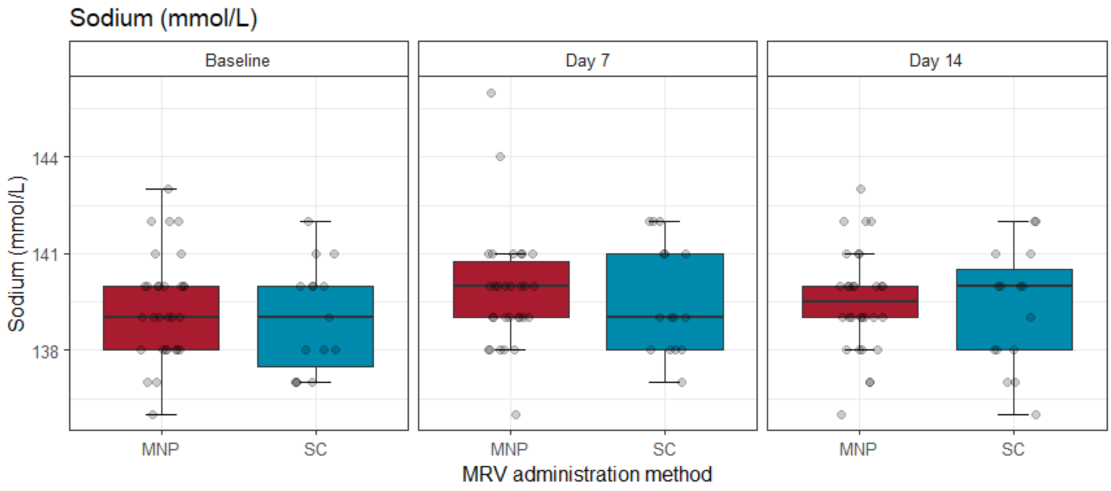
Sodium (mmol/L)
Baseline
Day 7
Day 14
Sodium (mmol/L)
144
141
138
MNP
SC
MRV administration method

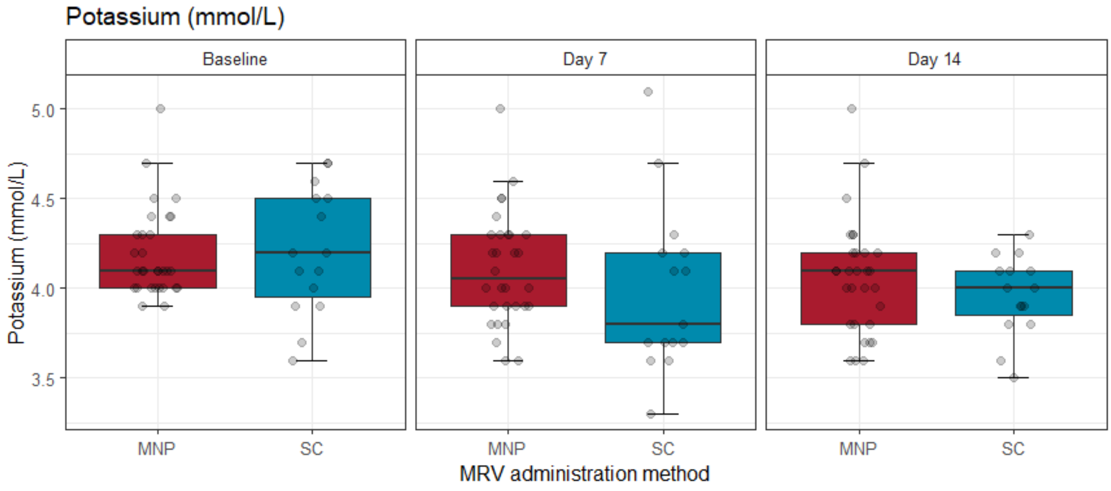
Potassium (mmol/L)
Baseline
Day 7
Day 14
Potassium (mmol/L)
5.0
4.5
4.0
3.5
MNP
SC
MRV administration method

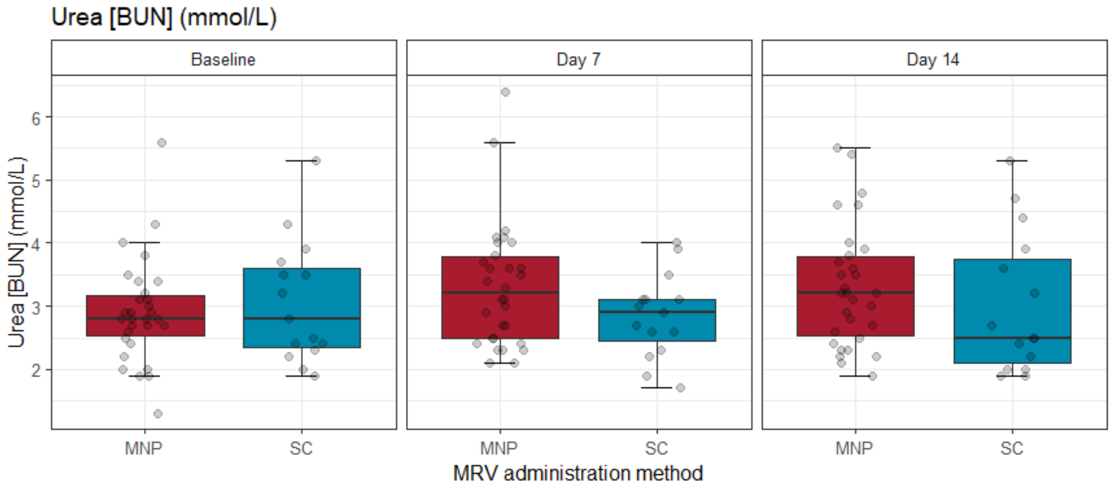
Urea [BUN] (mmol/L)
Baseline
Day 7
Day 14
Urea [BUN] (mmol/L)
6
5
4
3
2
MNP
SC
MRV administration method

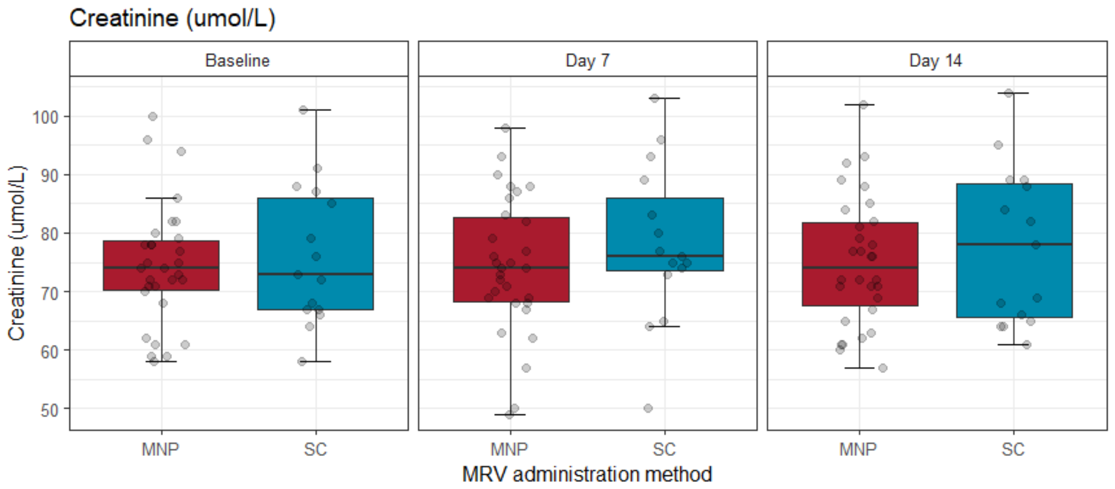
Creatinine (umol/L)
Baseline
Day 7
Day 14
Creatinine (umol/L)
100
90
80
70
60
50
MNP
SC
MRV administration method

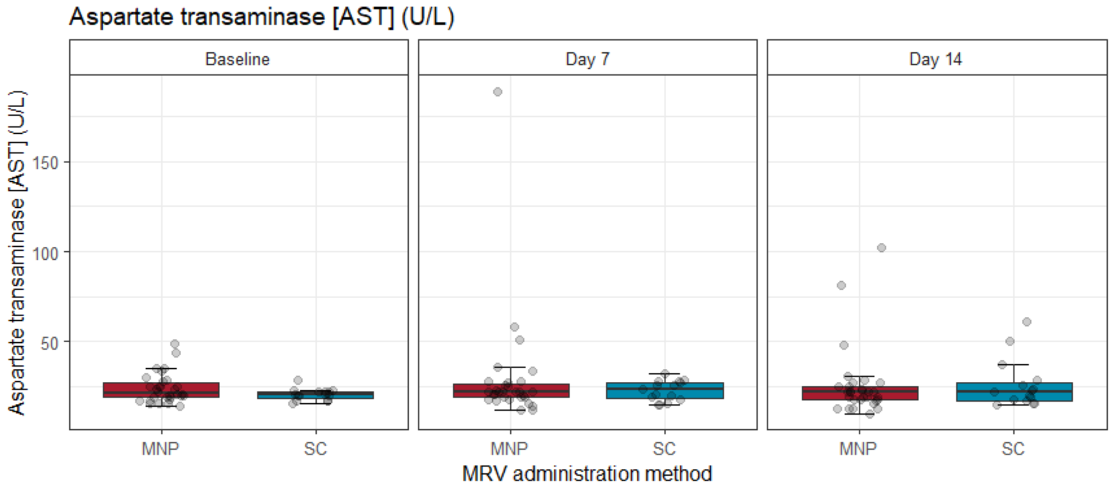
Aspartate transaminase [AST] (U/L)
Baseline
Day 7
Day 14
Aspartate transaminase [AST] (U/L)
150
100
50
MNP
SC
MRV administration method

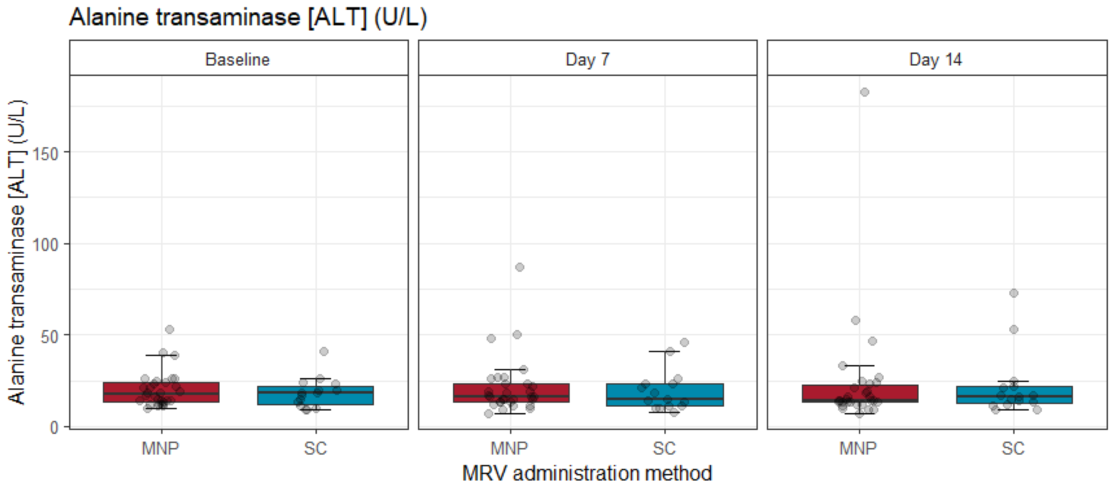
Alanine transaminase [ALT] (U/L)
Baseline
Day 7
Day 14
Alanine transaminase [ALT] (U/L)
150
100
50
0
MNP
SC
MRV administration method

Total bilirubin (umol/L)

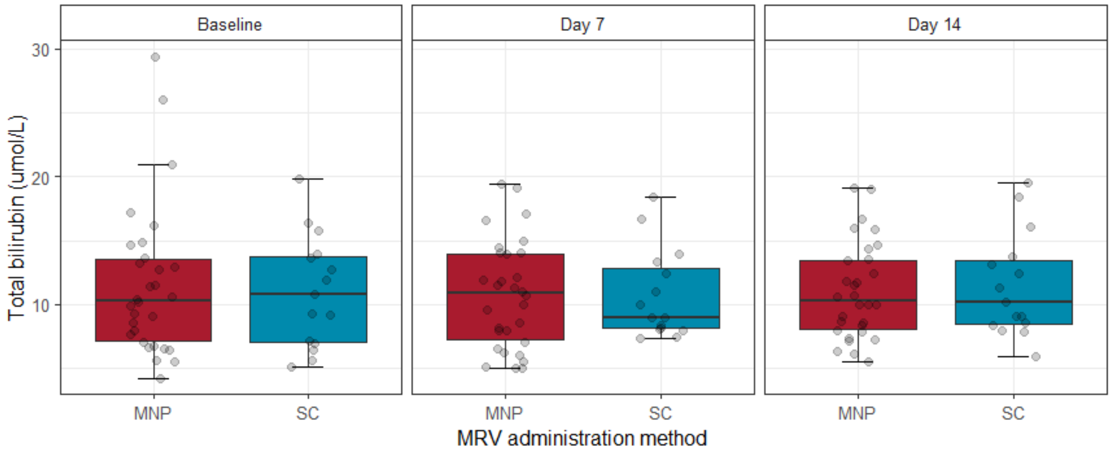

Total protein (g/L)

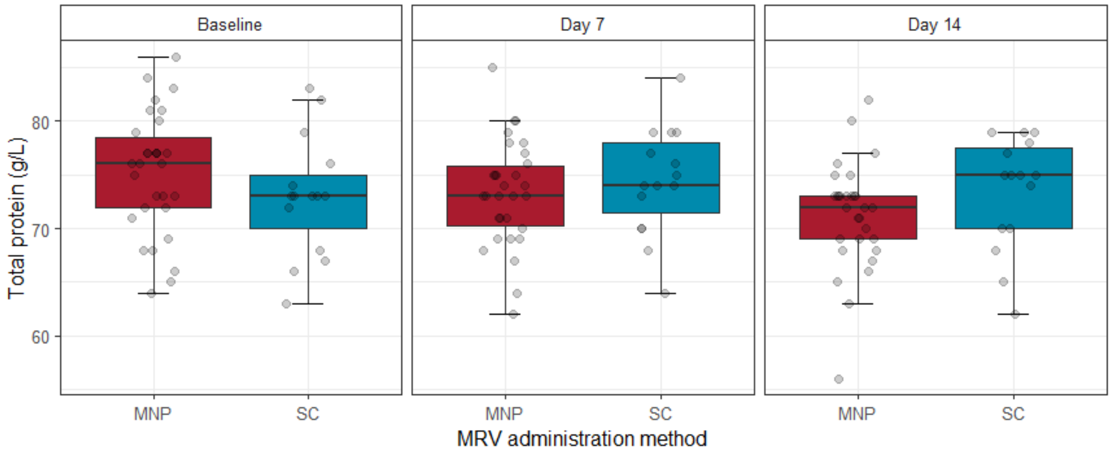

Albumin (g/L)

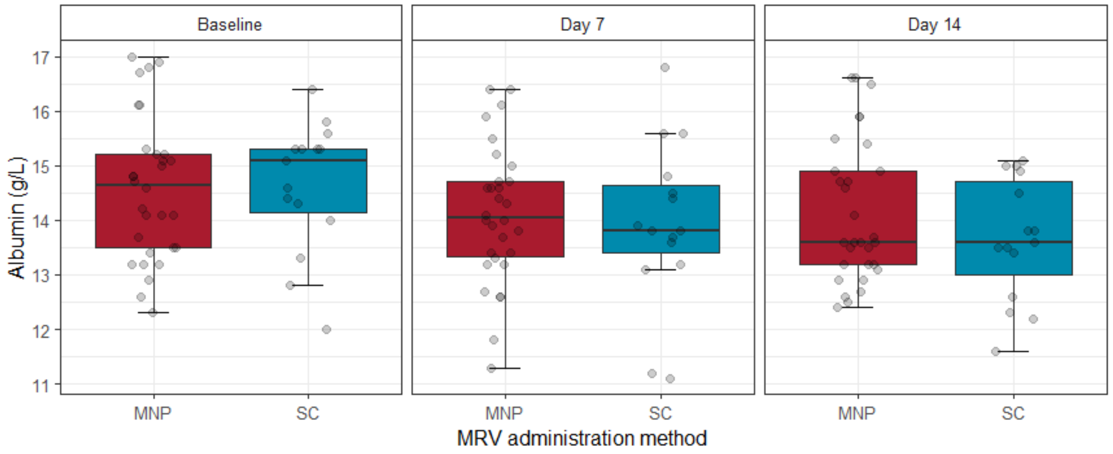

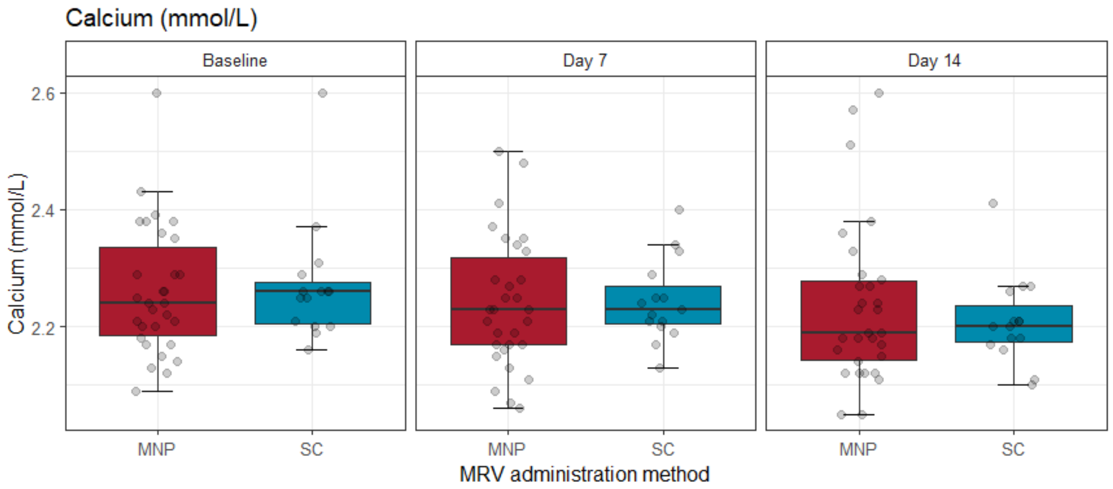

MRV – measles and rubella vaccine; MNP – measles and rubella vaccine microneedle patch; SC – measles and rubella vaccine by subcutaneous injection; g/dL – grams per decilitre; g/L – grams per litre; mmol/L – millimoles per litre; µmol/L – micromole per litre; U/L – units per litre; A box-and-whisker plot is a five-point data summary: bottom whisker = minimum value, box = lower quartile, median, upper quartile, and top whisker = maximum value. Values outside the range of the whiskers are outliers (defined as lower/upper quartile -/+ 1.5*interquartile range).

Toddler cohort

Haemoglobin (g/dL)

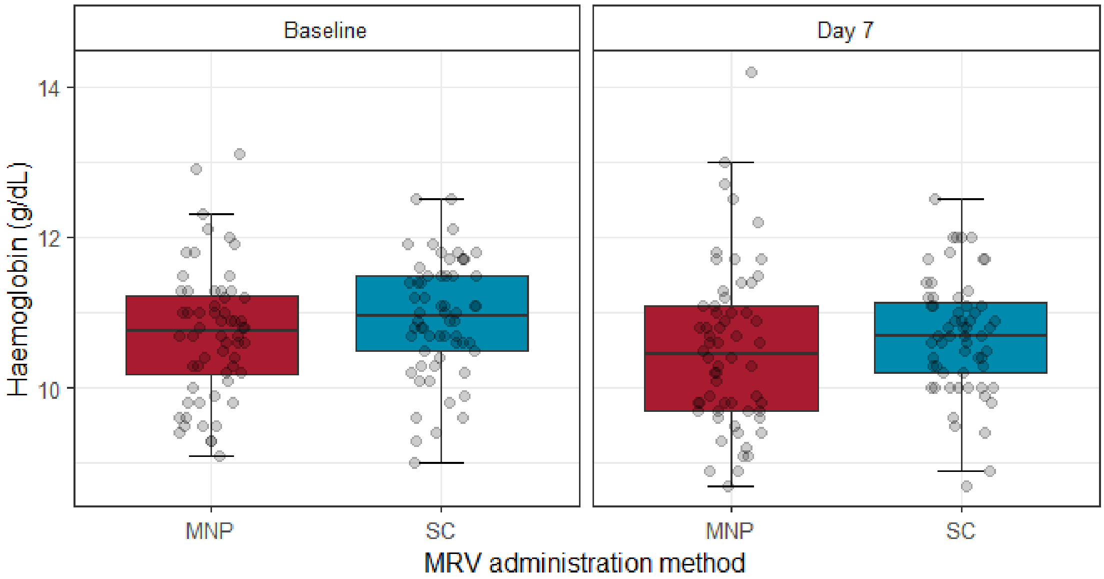

WBC total (x 10^9/L)

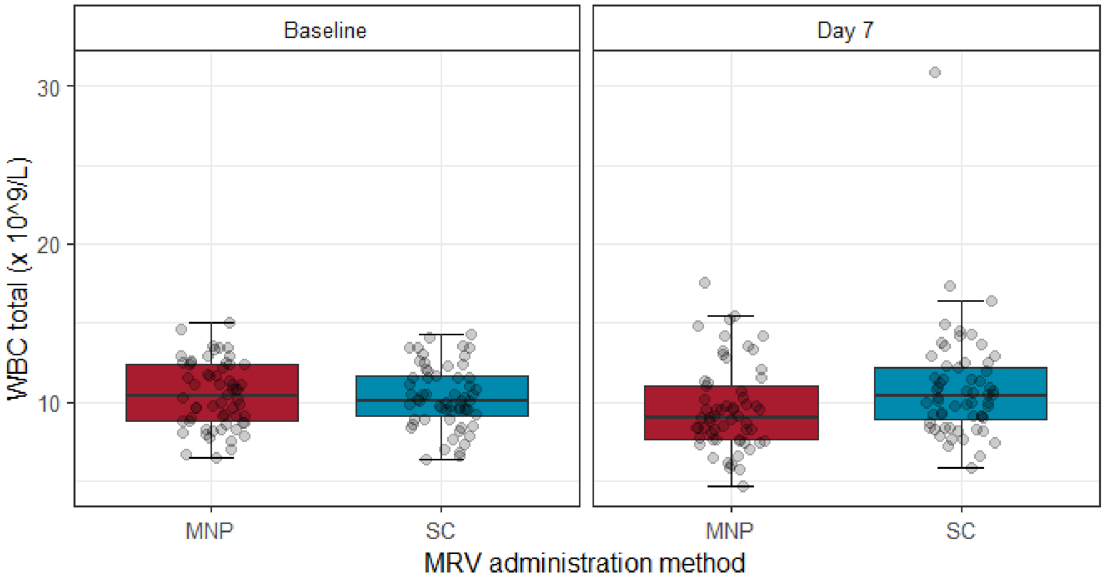

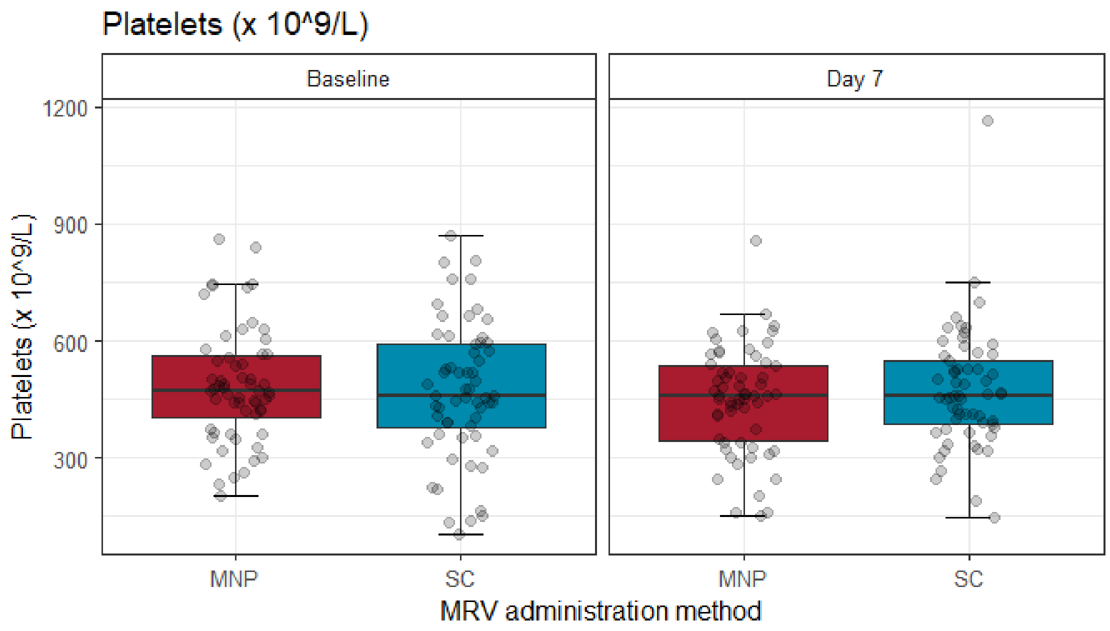
Platelets (x 10^9/L)
Baseline
Day 7
1200
900
600
300
Platelets (x 10^9/L)
MNP
SC
MNP
SC
MRV administration method

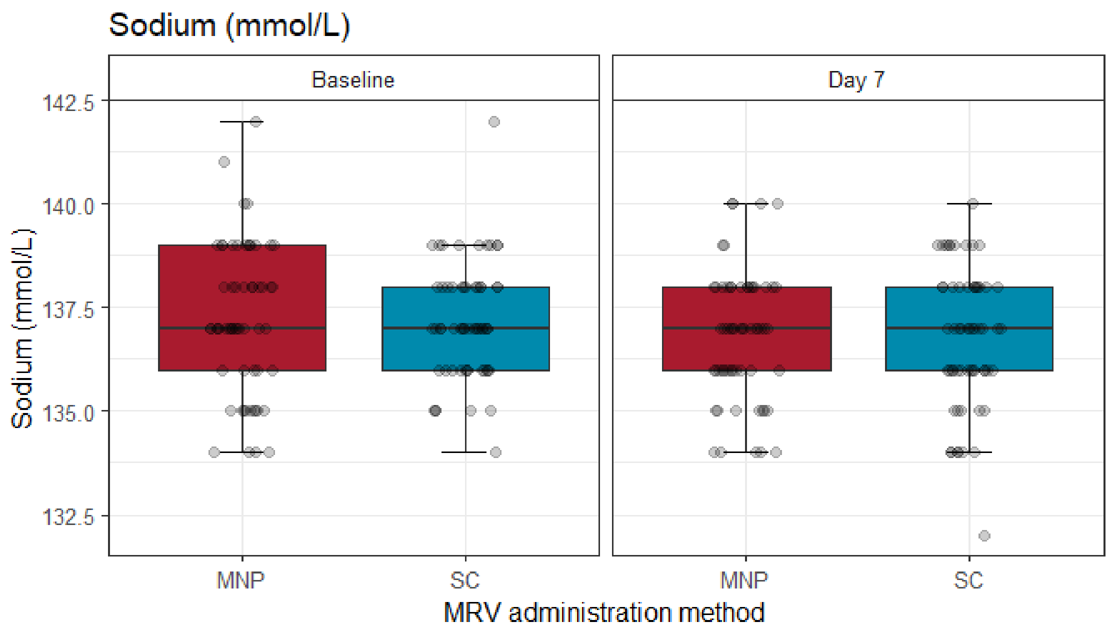
Sodium (mmol/L)
Baseline
Day 7
142.5
140.0
137.5
135.0
132.5
Sodium (mmol/L)
MNP
SC
MNP
SC
MRV administration method

Potassium (mmol/L)

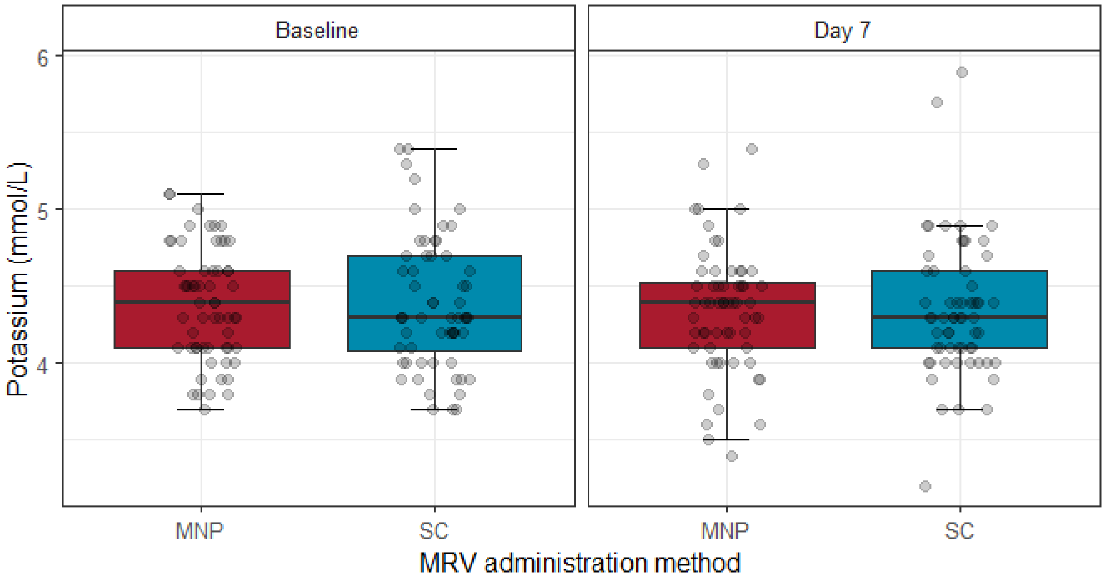

Urea [BUN] (mmol/L)

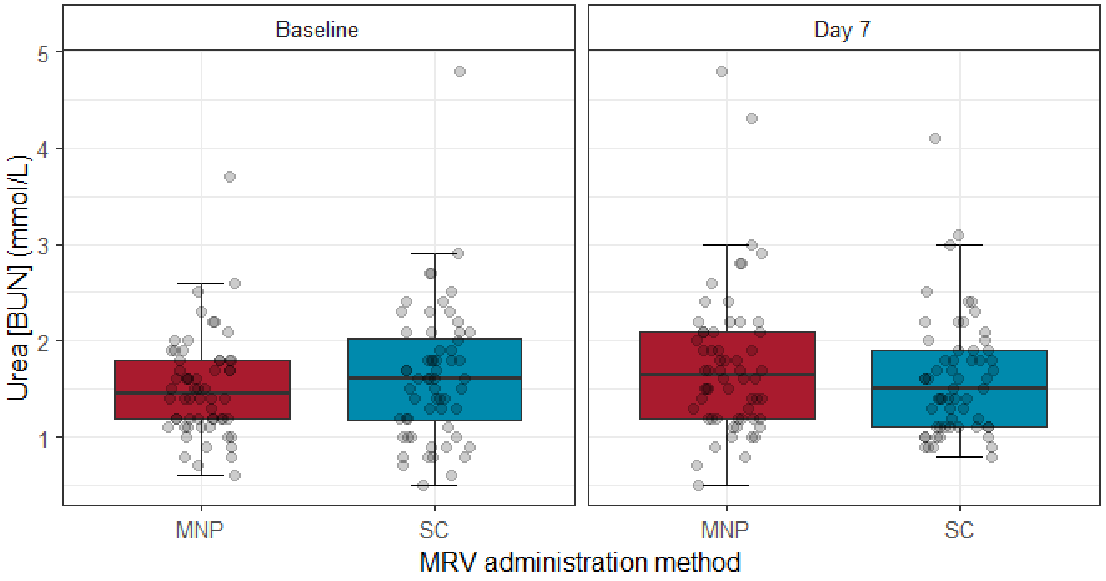

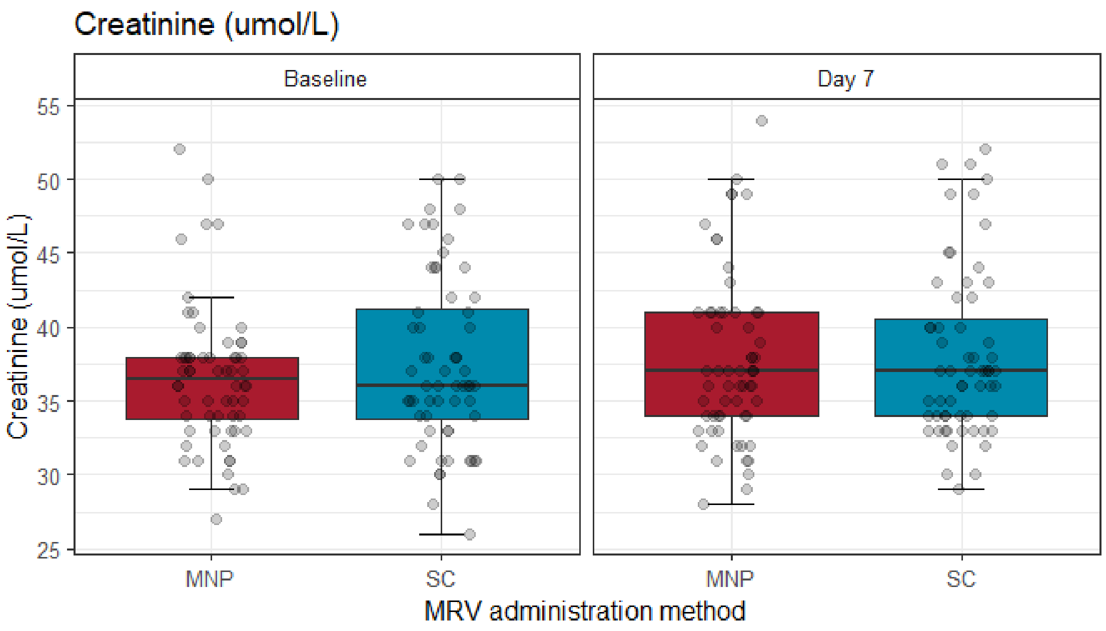
Creatinine (umol/L)
Baseline
Day 7
Creatinine (umol/L)
55
50
45
40
35
30
25
MNP
SC
MNP
SC
MRV administration method

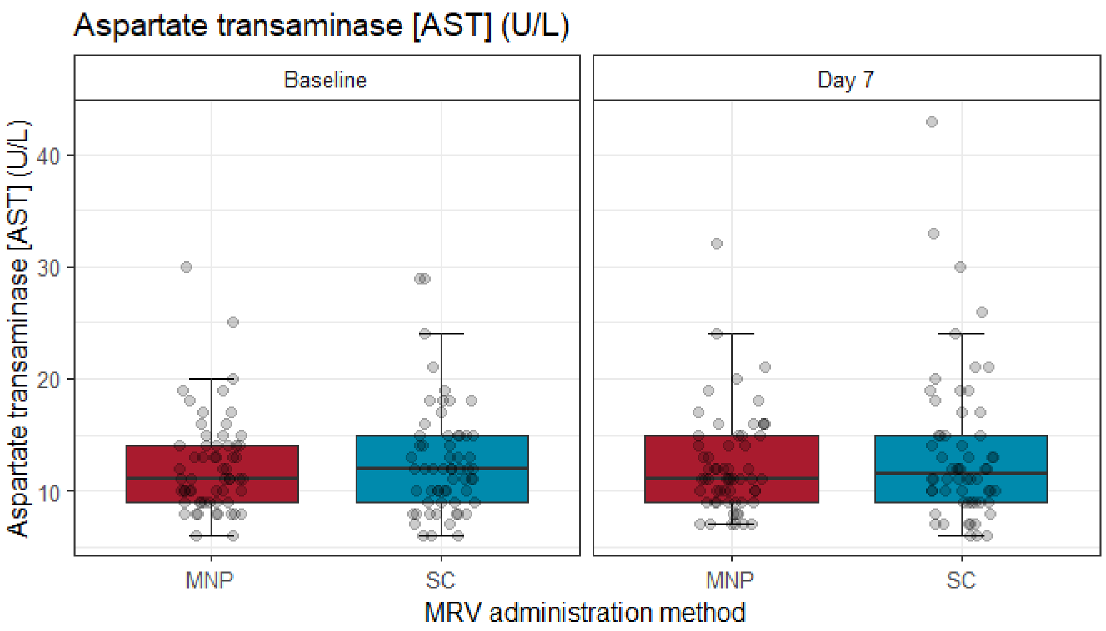
Aspartate transaminase [AST] (U/L)
Baseline
Day 7
Aspartate transaminase [AST] (U/L)
40
30
20
10
MNP
SC
MNP
SC
MRV administration method

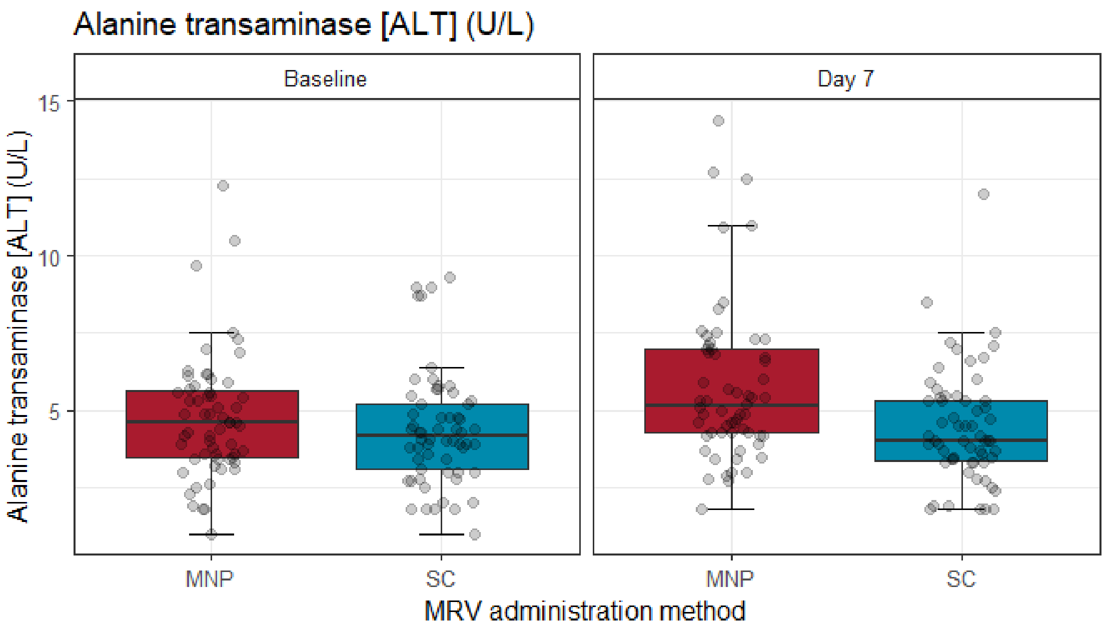
Alanine transaminase [ALT] (U/L)
Baseline
Day 7
Alanine transaminase [ALT] (U/L)
15
10
5
MNP
SC
MNP
SC
MRV administration method

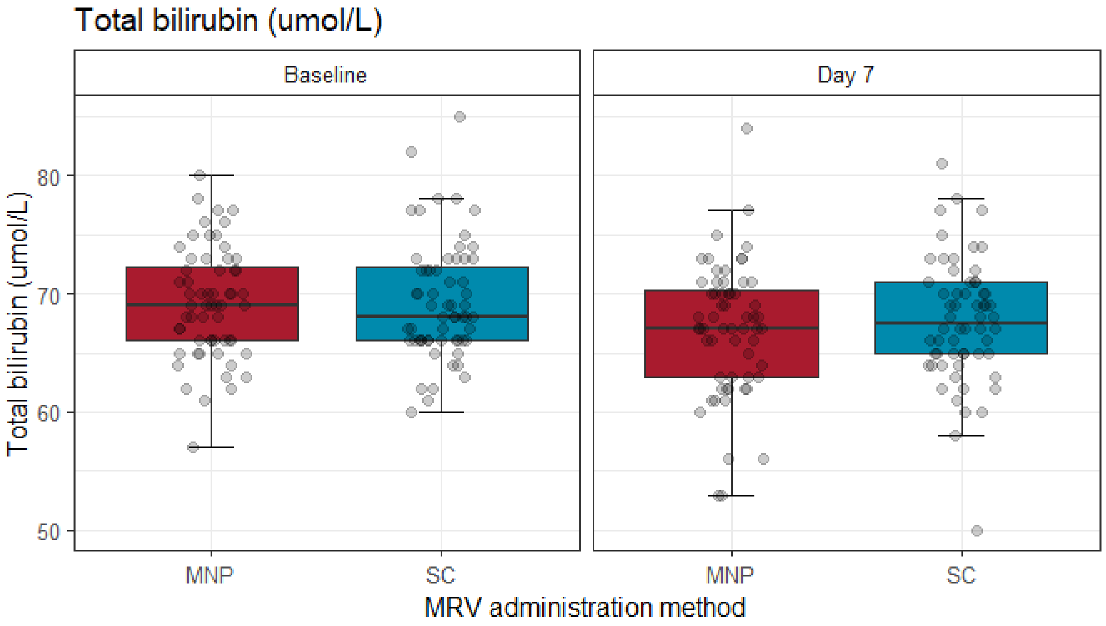
Total bilirubin (umol/L)
Baseline
Day 7
Total bilirubin (umol/L)
80
70
60
50
MNP
SC
MNP
SC
MRV administration method

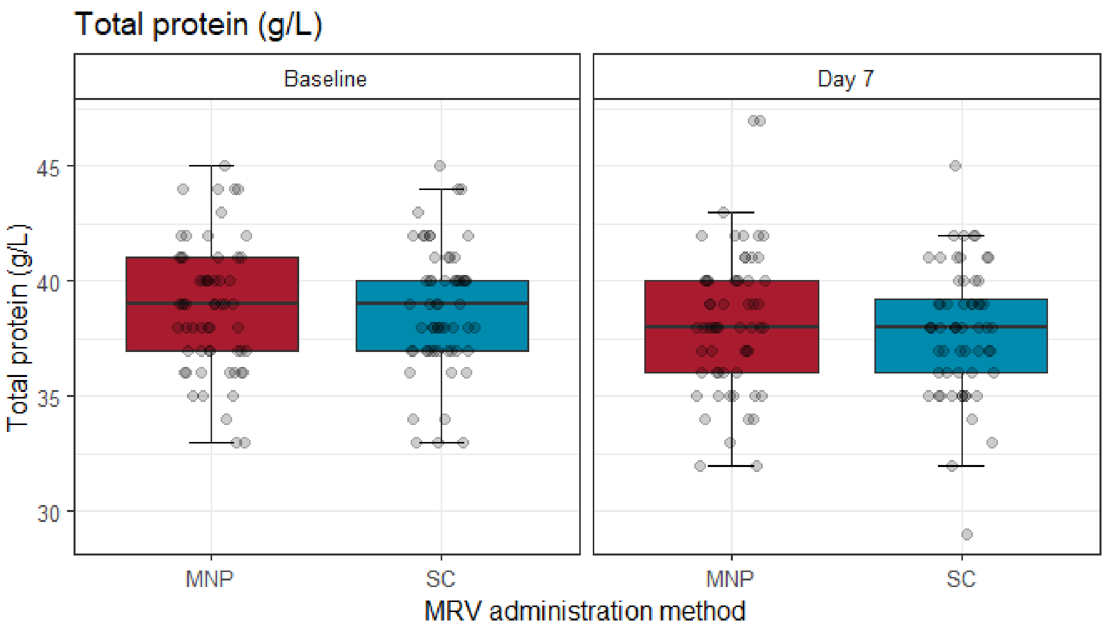

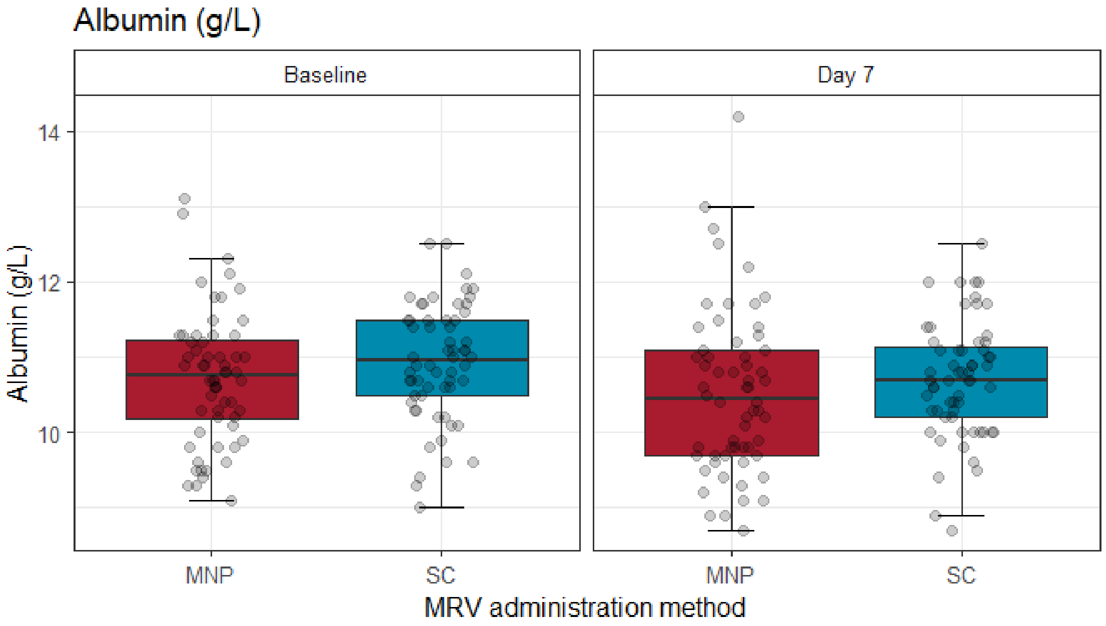

MRV – measles and rubella vaccine; MNP – measles and rubella vaccine microneedle patch; SC – measles and rubella vaccine by subcutaneous injection; g/dL – grams per decilitre; g/L – grams per litre; mmol/L – millimoles per litre; µmol/L – micromole per litre; U/L – units per litre; A box-and-whisker plot is a five-point data summary: bottom whisker = minimum value, box = lower quartile, median, upper quartile, and top whisker = maximum value. Values outside the range of the whiskers are outliers (defined as lower/upper quartile -/+ 1.5*interquartile range).

Infant cohort

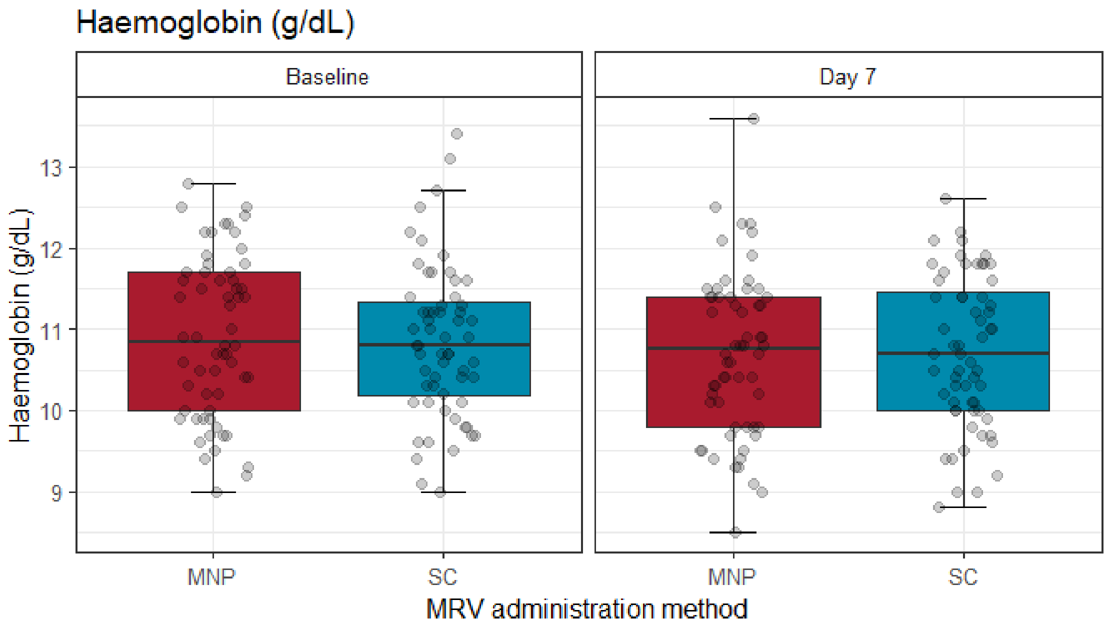

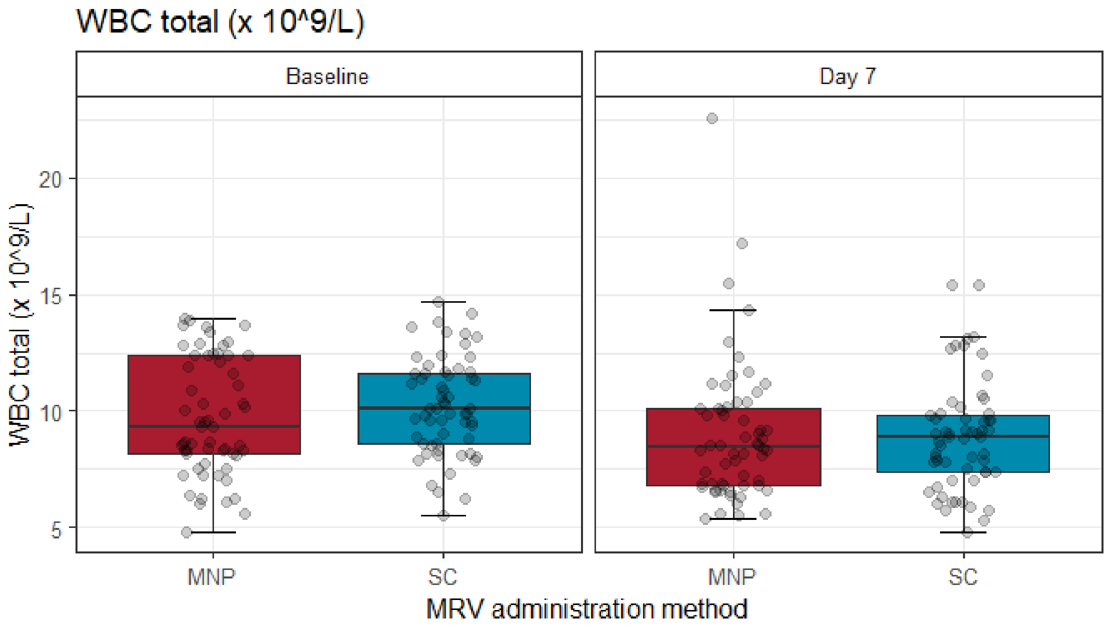

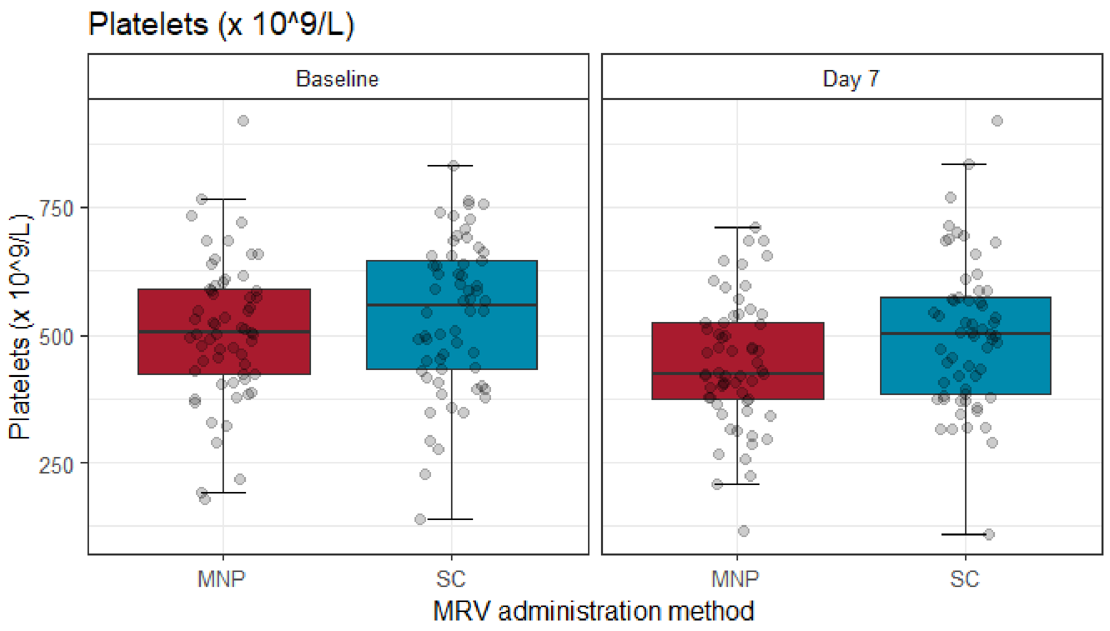
Platelets (x 10^9/L)
Baseline
Day 7
Platelets (x 10^9/L)
750
500
250
MNP
SC
MNP
SC
MRV administration method

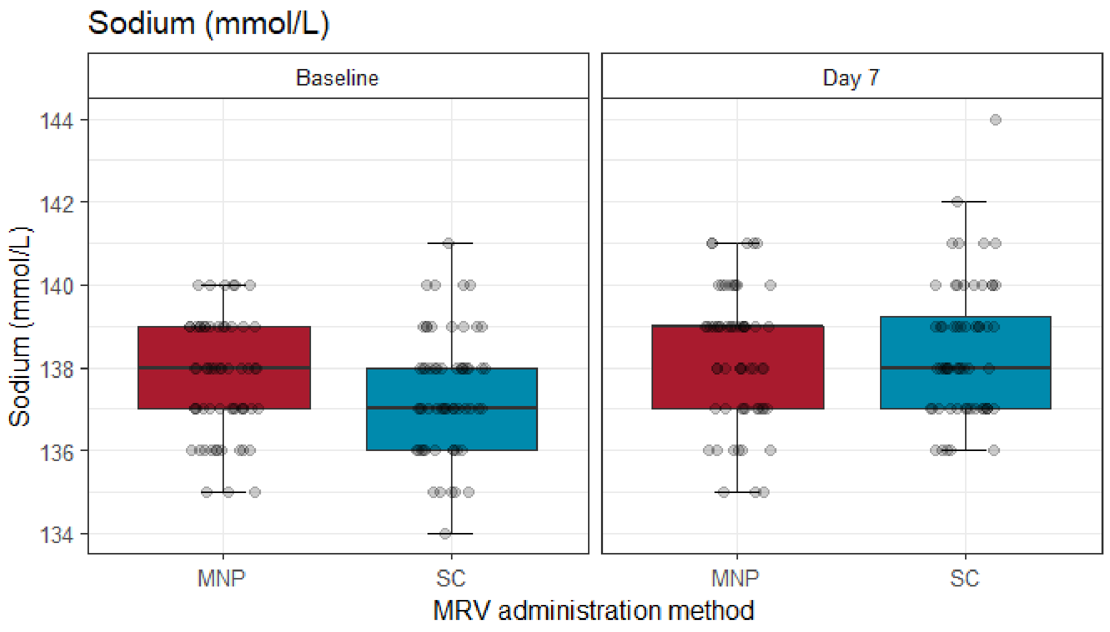
Sodium (mmol/L)
Baseline
Day 7
Sodium (mmol/L)
144
142
140
138
136
134
MNP
SC
MNP
SC
MRV administration method

Potassium (mmol/L)

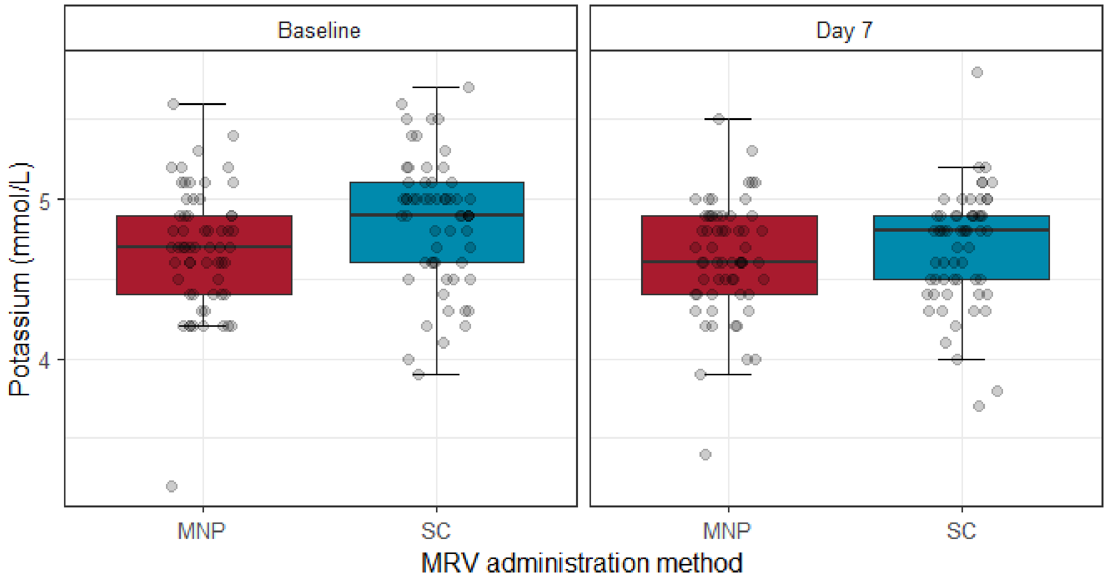

Urea [BUN] (mmol/L)

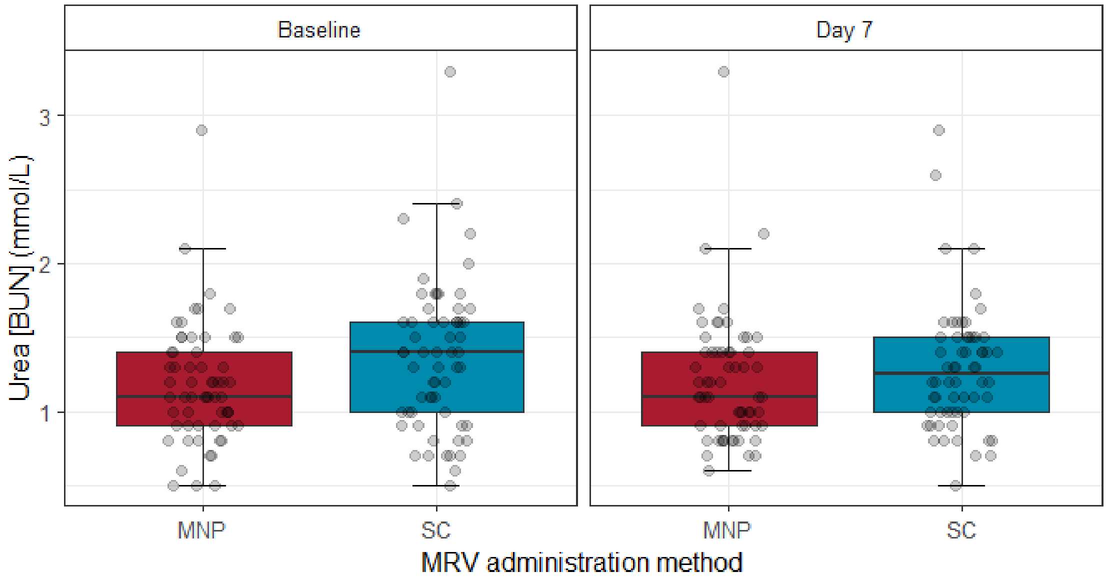

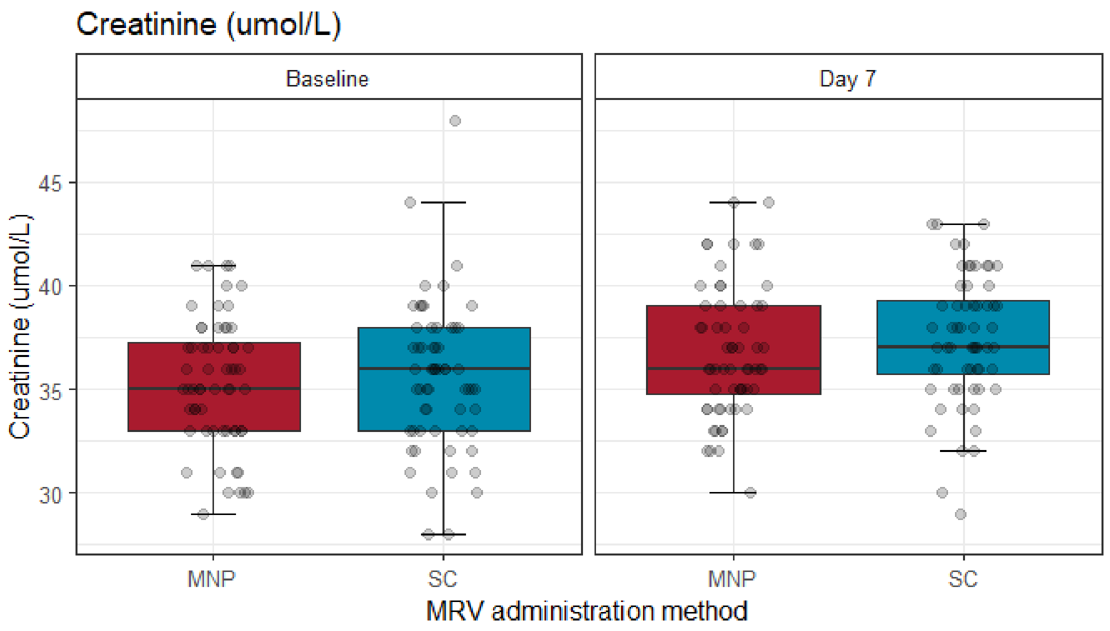
Creatinine (umol/L)
Baseline
Day 7
Creatinine (umol/L)
45
40
35
30
MNP
SC
MRV administration method

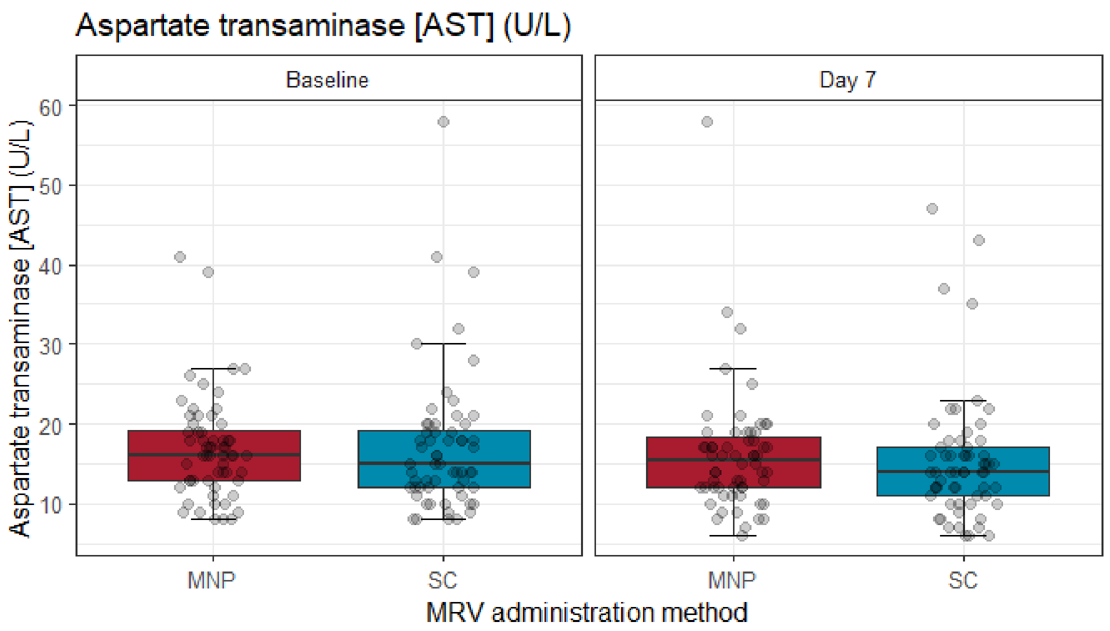
Aspartate transaminase [AST] (U/L)
Baseline
Day 7
Aspartate transaminase [AST] (U/L)
60
50
40
30
20
10
MNP
SC
MRV administration method

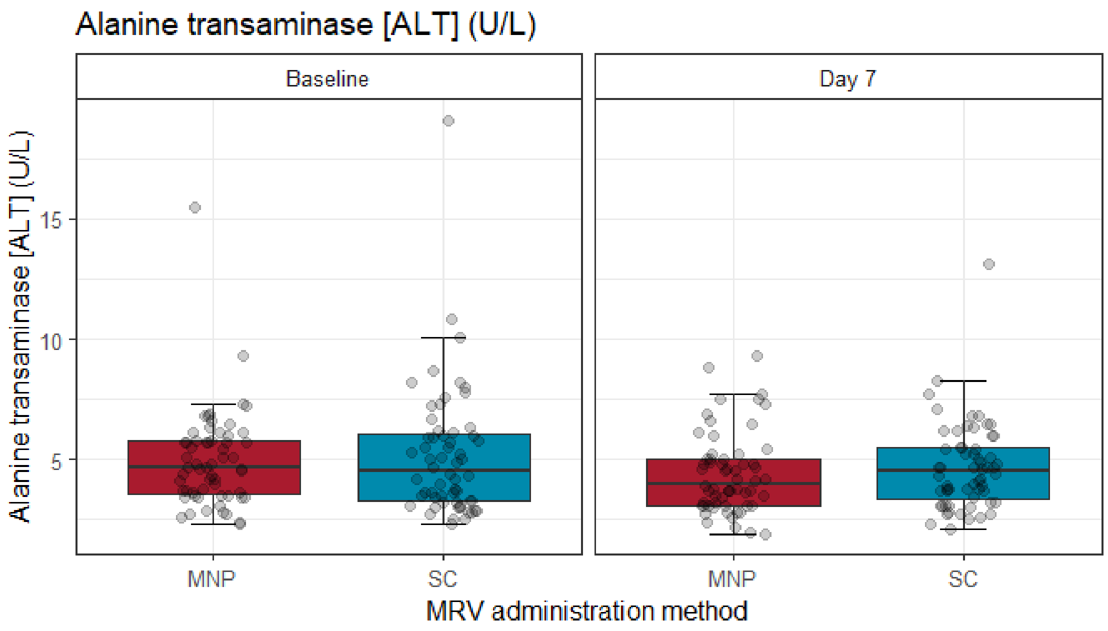
Alanine transaminase [ALT] (U/L)
Baseline
Day 7
Alanine transaminase [ALT] (U/L)
15
10
5
MNP
SC
MNP
SC
MRV administration method

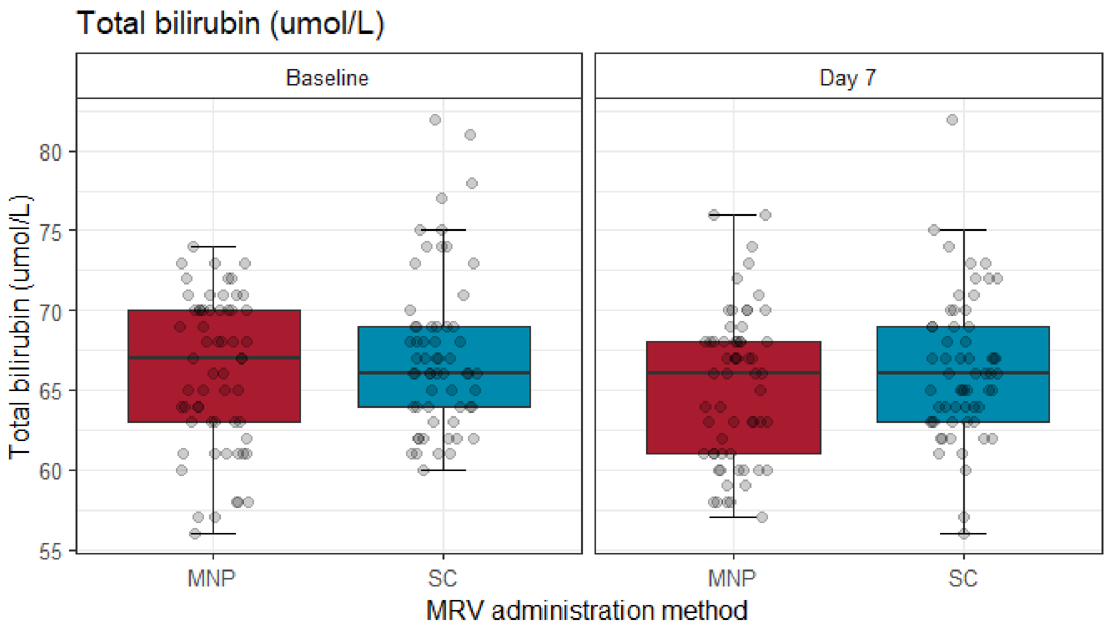
Total bilirubin (umol/L)
Baseline
Day 7
Total bilirubin (umol/L)
80
75
70
65
60
55
MNP
SC
MNP
SC
MRV administration method

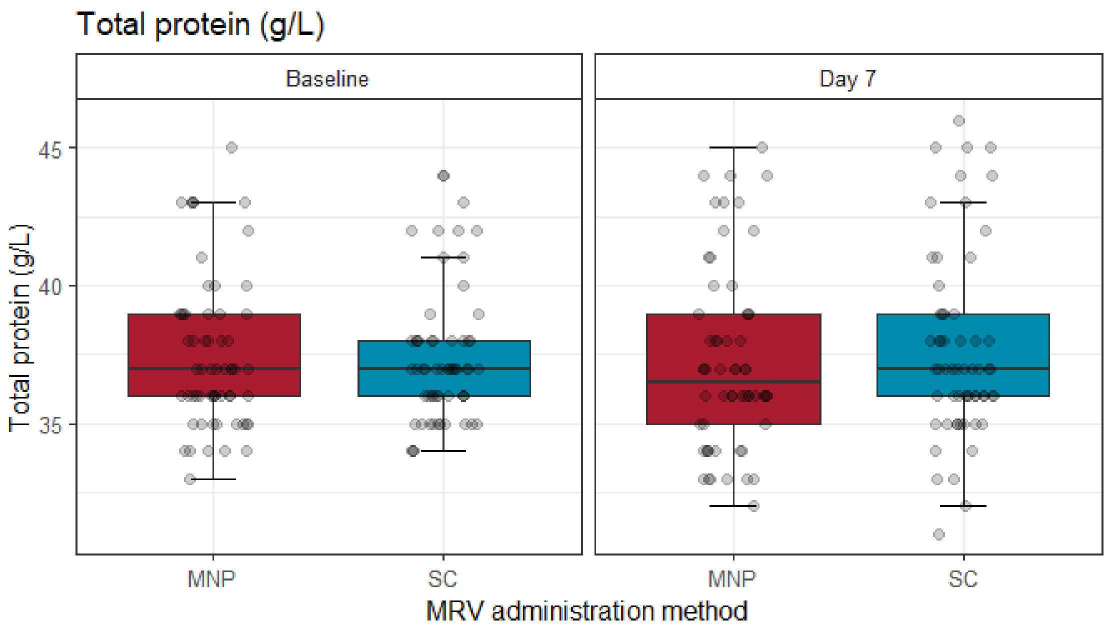

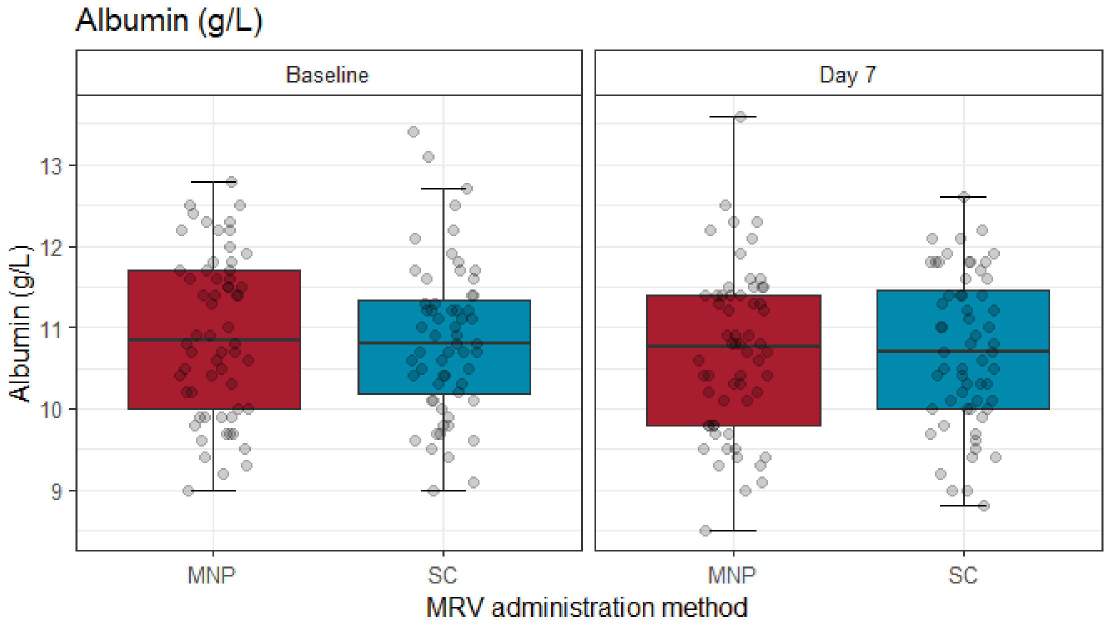

MRV – measles and rubella vaccine; MNP – measles and rubella vaccine microneedle patch; SC – measles and rubella vaccine by subcutaneous injection; g/dL – grams per decilitre; g/L – grams per litre; mmol/L – millimoles per litre; µmol/L – micromole per litre; U/L – units per litre; A box-and-whisker plot is a five-point data summary: bottom whisker = minimum value, box = lower quartile, median, upper quartile, and top whisker = maximum value. Values outside the range of the whiskers are outliers (defined as lower/upper quartile -/+ 1.5*interquartile range).

Supplementary Table: Solicited safety data, day 0 to 13 – adult cohort.

		Adults	
		**MRV-MNP & placebo SC**	**MRV-SC & placebo MNP**
		**N = 30**	**N = 15**
		**n (%)**	**n (%)**
**Acute allergic reaction**		0 (0.0)	0 (0.0)
**Local solicited adverse events**			
**Microneedle patch application site**		**Measles and Rubella MNP**	**Placebo MNP**
Any local solicited event¶	Total	8 (26.7)	3 (20.0)
	Mild (grade 1)	8 (26.7)	3 (20.0)
Pain	Total	3 (10.0)	2 (13.3)
	Mild (grade 1)	3 (10.0)	2 (13.3)
Erythema	Total	0 (0.0)	0 (0.0)
Induration	Total	0 (0.0)	0 (0.0)
Pruritis	Total	5 (16.7)	1 (6.7)
	Mild (grade 1)	5 (16.7)	1 (6.7)
**SC injection site**		**Placebo SC**	**Measles and Rubella SC**
Any local solicited event¶	Any reaction	7 (23.3)	1 (6.7)
	Mild (grade 1)	7 (23.3)	1 (6.7)
**Systemic solicited adverse events**			
Fever	Total	0 (0.0)	0 (0.0)
Any systemic solicited event ‡	Total	15 (50.0)	7 (46.7)
	Mild (grade 1)	13 (43.3)	7 (46.7)
	Moderate (grade 2)	2 (6.7)	0 (0.0)

MNP – microneedle patch; SC – subcutaneous; N – number of participants included in analysis; n – number of participants experiencing event by maximum severity grading; ¶ Pain, erythema, induration, pruritus; ‡ vomiting, diarrhoea, headache, fatigue, myalgia, arthralgia, rash.

Supplementary Table: Unsolicited adverse event data – adult cohorts

		Adult			
		**MRV-MNP & placebo SC**		**MRV-SC & placebo MNP**	
		**N = 30**		**N = 15**	
		**n (%)**	**E**	**n (%)**	**E**
**Adverse events**	Total	25 (83.3)	69	15 (100.0)	46
	Mild (grade 1)	19 (63.3)	61	9 (60.0)	40
	Moderate (grade 2)	5 (16.7)	7	4 (26.7)	4
	Severe (grade 3)	1 (3.3)	1	2 (13.3)	2
**Serious adverse events**		1 (3.3)	1	2 (13.3)	2
**Adverse events resulting in discontinuation from the study**		0 (0.0)	0	0 (0.0)	0
**Related adverse events**	Total	16 (53.5)	17	2 (13.3)	2
	Mild (grade 1)	16 (53.5)	17	2 (13.3)	2
	Microneedle patch site discolouration	12 (40.0)	12	2 (13.3)	2
	Microneedle patch site exfoliation	2 (6.7)	2	0 (0.0)	0
	Microneedle patch site induration	3 (10.0)	3	0 (0.0)	0
**Related serious adverse events**		0 (0.0)	0	0 (0.0)	0

MNP – microneedle patch; SC – subcutaneous; N – number of participants included in analysis; n (%) – number and percentage of participants experiencing event by maximum severity grading; E – number of events by maximum severity grade.

Supplementary Figure: Unsolicited adverse events – adult cohort

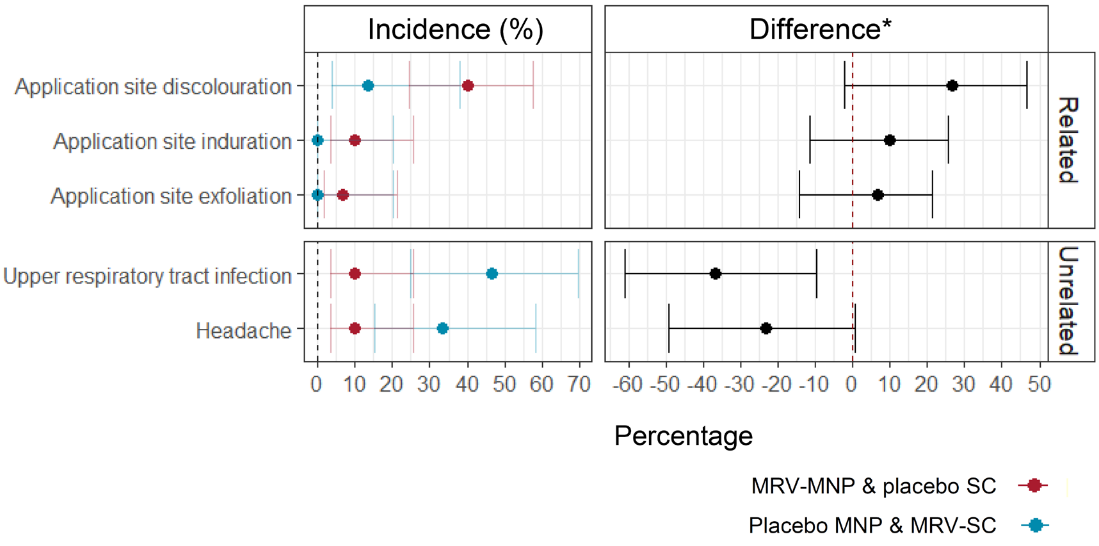

Only events which occurred in at least 3 participants in the adult age cohort are included in the figure; MNP – microneedle patch; * Percentage prevalence in measles and rubella MNP group minus percentage prevalence in placebo MNP group.

Supplementary Table: Duration of related unsolicited MNP adverse events

Tables indicate the number and percentage of all unsolicited adverse events judged to be related to vaccination ongoing at the given timepoints. Application site reactions were only reported as unsolicited events if present on day 14.

Adults

	MRV-MNP (N=30)			Placebo MNP (N=15)		
	n (%) of events ongoing at timepoints					
	Day 14	Day 42	Day 180	Day 14	Day 42	Day 180
**Application site discolouration**	12 (40.0)	2 (6.7)	0 (0.0)	2 (13.3)	0 (0.0)	0 (0.0)
**Application site exfoliation**	3 (10.0)	0 (0.0)	0 (0.0)	0 (0.0)	0 (0.0)	0 (0.0)
**Application site induration**	2 (6.7)	2 (6.7)	0 (0.0)	0 (0.0)	0 (0.0)	0 (0.0)

Toddlers

	MRV-MNP (N=60)			Placebo MNP (N=60)		
	n (%) of events ongoing at timepoints					
	Day 14	Day 42	Day 180	Day 14	Day 42	Day 180
**Application site discolouration**	29 (48.3)	9 (15.0)	0 (0.0)	12 (20.0)	2 (3.3)	0 (0.0)
**Application site exfoliation**	5 (8.3)	0 (0.0)	0 (0.0)	1 (1.7)	0 (0.0)	0 (0.0)
**Application site induration**	3 (5.0)	0 (0.0)	0 (0.0)	0 (0.0)	0 (0.0)	0 (0.0)
**Other**	6 (10.0) ‡	2 (3.3)	1 (1.7)	3 (5.0) ¶	0 (0.0)	0 (0.0)

‡Microneedle patch site papules (n = 2), microneedle patch site pruritis (n = 1), SC injection site induration (n = 2); Generalized papular rash (n = 1). ¶ Microneedle patch site papules (n = 1), generalized maculopapular rash (n = 2)

Infants

	MRV-MNP (N=60)			Placebo MNP (N=60)		
	n (%) of events ongoing at timepoints					
	Day 14	Day 42	Day 180	Day 14	Day 42	Day 180
**Application site discolouration**	50 (83.3)	22 (36.7)	5 (8.3)	32 (53.3)	1 (1.7)	0 (0.0)
**Application site exfoliation**	14 (23.3)	1 (1.7)	0 (0.0)	6 (10.0)	0 (0.0)	0 (0.0)
**Application site induration**	7 (11.7)	0 (0.0)	0 (0.0)	1 (1.7)	0 (0.0)	0 (0.0)
**Other**	4 (6.7) †	0 (0.0)	0 (0.0)	2 (3.3) β	0 (0.0)	0 (0.0)

†Microneedle patch site macule (n = 1), generalized rash (n = 1), generalized papular rash (n = 1); poor infant feeding (n = 1); β Microneedle patch site papules (n = 1), diarrhoea (n = 1).

Supplementary Table: Serious adverse event listing

	Treatment Group	MedDRA Preferred Term	MedDRA System Organ Class	SAE criteria	Interval between MNP administration and SAE onset (days)	Duration (days)β	Outcome	Severity	Relatedness
	**Adults**								
1	Measles and rubella vaccine subcutaneously & placebo MNP	Gastroenteritis	Infections and infestations	Required inpatient hospitalization.	60	3	Resolved without sequelae	Severe	Not related
2	Measles and rubella vaccine subcutaneously & placebo MNP	Pelvic Inflammatory Disease	Infections and infestations	Required inpatient hospitalization.	46	13	Resolved without sequelae	Severe	Not related
3	Measles and rubella vaccine MNP & placebo subcutaneously	Febrile infection	Infections and infestations	Required inpatient hospitalization.	82	12	Resolved without sequelae	Severe	Not related
	**Toddlers**								
1§	Measles and rubella vaccine subcutaneously & placebo MNP	Gastroenteritis	Infections and infestations	Required inpatient hospitalization.	5	10	Resolved without sequelae	Moderate	Not related
2§	Measles and rubella vaccine subcutaneously & placebo MNP	Infant irritability	Nervous system disorders	Required inpatient hospitalization.	177	1	Resolved without sequelae	Severe	Not related
3	Measles and rubella vaccine subcutaneously & placebo MNP	Malaria	Infections and infestations	Required inpatient hospitalization.	22	7	Resolved without sequelae	Moderate	Not related
4	Measles and rubella vaccine subcutaneously & placebo MNP	Pneumonia	Infections and infestations	Required inpatient hospitalization.	7	7	Resolved without sequelae	Severe	Not related
5	Measles and rubella vaccine subcutaneously & placebo MNP	Seizure	Nervous system disorders	Required inpatient hospitalization.	155	1	Resolved without sequelae	Severe	Not related
6	Measles and rubella vaccine subcutaneously & placebo MNP	Cellulitis	Infections and infestations	Required inpatient hospitalization.	27	15	Resolved without sequelae	Moderate	Not related
7	Measles and rubella vaccine subcutaneously & placebo MNP	Bronchiolitis	Infections and infestations	Required inpatient hospitalization.	57	14	Resolved without sequelae	Severe	Not related

	Treatment Group	MedDRA Preferred Term	MedDRA System Organ Class	SAE criteria	Interval between MNP administration and SAE onset (days)	Duration (days)β	Outcome	Severity	Relatedness
8	Measles and rubella vaccine MNP & placebo subcutaneously	Cellulitis	Infections and infestations	Required inpatient hospitalization.	164	20	Resolved without sequelae	Severe	Not related
9	Measles and rubella vaccine subcutaneously & placebo MNP	Febrile convulsion	Nervous system disorders	Required inpatient hospitalization.	27	0‡	Resolved without sequelae	Severe	Not related
	**Infants**								
1	Measles and rubella vaccine MNP & placebo subcutaneously	Chemical poisoning	Injury, poisoning and procedural complications	Required inpatient hospitalization.	33	1	Resolved without sequelae	Moderate	Not related
2	Measles and rubella vaccine subcutaneously & placebo MNP	Lymphadenitis	Blood and lymphatic system disorders	Required inpatient hospitalization.	148	25	Resolved without sequelae	Severe	Not related

MNP – microneedle patch; MedDRA – Medical Dictionary for Regulatory Activities; § two separate SAE in the same participant; ‡ resolved on the same day as onset; β SAE criterion met to AE resolution.

Supplementary Table: Measles and rubella serum neutralizing antibody data – adult primary immunogenicity population

		Measles			Rubella		
		**MRV-MNP, PLA-SC**	**PLA-MNP, MRV-SC**	**Ratio§/ Difference‡**	**MRV-MNP, PLA-SC**	**PLA-MNP, MRV-SC**	**Ratio§/ Difference‡**
**Baseline**	**Median (Q1-Q3)**	250 (100-455)	204 (103-372)		231 (109-327)	247 (168-496)	
	GMC (95% CI)	242.7 (143.4 to 410.7)	199.2 (130.6 to 303.8)	1.22§ (0.634 to 2.342)	172.0 (123.9 to 238.7)	279.0 (194.4 to 400.3)	0.62§ (0.385 to 0.988)
	Seroprotection (n/N) % (95% CI)	(19/30) 63.3 (45.5 to 78.1)	(8/15) 53.3 (30.1 to 75.2)	10.0‡ (-18.2 to 37.5)	(30/30) 100.0 (88.7 to 100.0)	(15/15) 100.0 (79.6 to 100.0)	0.0‡ (-11.4 to 20.4)
	N	30	15		30	15	
**Visit 4 (Day 42)**	**Median (Q1-Q3)**	1160 (742-1683)	660 (434-788)		390 (295-474)	390 (197-508)	
	GMC (95% CI)	1107.4 (839.3 to 1461.3)	590.2 (424.9 to 819.7)	1.88§ (1.238 to 2.843)	354.3 (302.1 to 415.6)	342.0 (227.9 to 513.4)	1.04§ (0.675 to 1.591)
	GMFR (95% CI)	4.6 (3.0 to 6.8)	3.0 (1.9 to 4.7)	1.54§ (0.851 to 2.786)	2.1 (1.6 to 2.7)	1.2 (0.8 to 1.8)	1.68§ (1.080 to 2.618)
	Seroprotection (n/N) % (95% CI)	(29/30) 96.7 (83.3 to 99.4)	(14/15) 93.3 (70.2 to 98.8)	3.3‡ (-11.1 to 26.7)	(30/30) 100.0 (88.7 to 100.0)	(15/15) 100.0 (79.6 to 100.0)	0.0‡ (-11.4 to 20.4)
	Seroconversion (n/N) % (95% CI)	(10/11) 90.9 (62.3 to 98.4)	(6/7) 85.7 (48.7 to 97.4)	5.2‡ (-25.8 to 43.0)	-	-	-
	n (Baseline seronegative)	11	7		0	0	
	4-Fold Rise (n/N) % (95% CI)	(5/19) 26.3 (11.81 to 48.79)	(0/8) 0.0 (0.00 to 32.44)	26.3‡ (-9.22 to 48.79)	(4/30) 13.3 (5.31 to 29.68)	(1/15) 6.7 (1.19 to 29.82)	6.7‡ (-17.83 to 23.91)
	n (Baseline seropositive)	19	8		30	15	
	Immune Response (n/N) % (95% CI)	(15/30) 50.0 (33.2 to 66.9)	(6/15) 40.0 (19.8 to 64.3)	10.0‡ (-19.5 to 36.3)	(4/30) 13.3 (5.3 to 29.7)	(1/15) 6.7 (1.2 to 29.8)	6.7‡ (-17.8 to 23.9)
	Total n	30	15		30	15	
**Visit 5 (Day 180)**	**Median (Q1-Q3)**	**618 (429-1155)**	**370 (324-549)**		**318 (179-429)**	**426 (180-532)**	
	GMC (95% CI)	700.4 (512.4 to 957.4)	372.7 (266.7 to 520.9)	1.88§ (1.207 to 2.926)	282.9 (224.1 to 357.1)	322.2 (213.6 to 486.1)	0.88§ (0.554 to 1.391)
	Seroprotection (n/N) % (95% CI)	(28/30) 93.3 (78.7 to 98.2)	(12/15) 80.0 (54.8 to 93.0)	13.3‡ (-6.2 to 39.0)	(30/30) 100.0 (88.7 to 100.0)	(30/30) 100.0 (79.6 to 100.0)	0.0‡ (-11.4 to 20.4)
	Total n	30	15		30	15	

Q1 – Lower quartile; Q3 – Upper quartile; CI confidence interval; n – number included in given analysis; GMC - geometric mean antibody concentrations; GMFR - geometric mean fold rise; NA – not applicable; baseline and visit 4 (day 42) analysis are in the primary immunogenicity population; visit 5 (day 180) analysis is in the day 180 secondary immunogenicity population; seroconversion is defined as a change from seronegative at baseline to seropositive at day 42; 4-fold rise is defined as a 4-fold rise in antibody concentrations between baseline and day 42 amongst individuals who were seropositive at baseline; immune response includes all those who were seronegative at baseline and seroconverted on day 42 or who were seropositive at baseline and had a 4-fold rise in antibody concentrations; for measles, seronegative is defined as an antibody concentration of < 200 mIU/mL, seropositive/seroprotection is defined as an antibody concentration of ≥ 200 mIU/mL; for rubella, seronegative is defined as an antibody concentration of < 10 IU/mL and seropositive/seroprotection is defined as an antibody concentration of ≥ 10 IU/mL; §Ratio [microneedle patch]/[subcutaneous injection]; ‡ Difference [microneedle patch] – [subcutaneous injection]. Estimates are presented with 95% confidence intervals. CIs for the log2 transformed means assume a Student's t-distribution. CIs for seroprotection and seroconversion were calculated using the Wilson score method without continuity correction. CIs for differences between proportions were calculated using the Newcombe method without continuity correction.

Supplementary Figure: Typical mild MRV-MNP application site reaction time series (single infant).

Day 0
Pre-MNP application

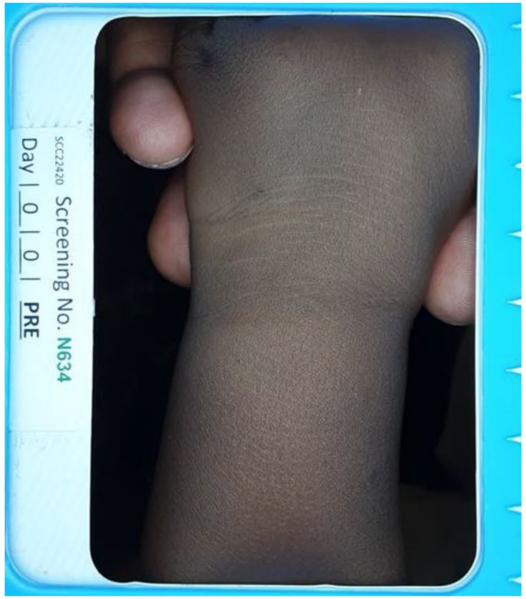

Day 7
Post-MNP application

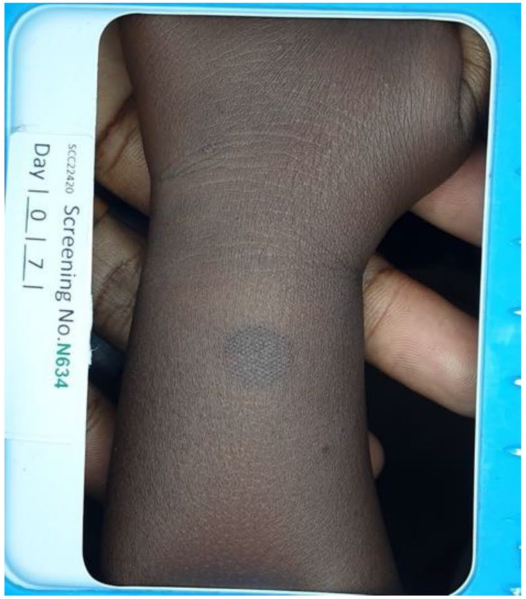

Day 42
Post-MNP application

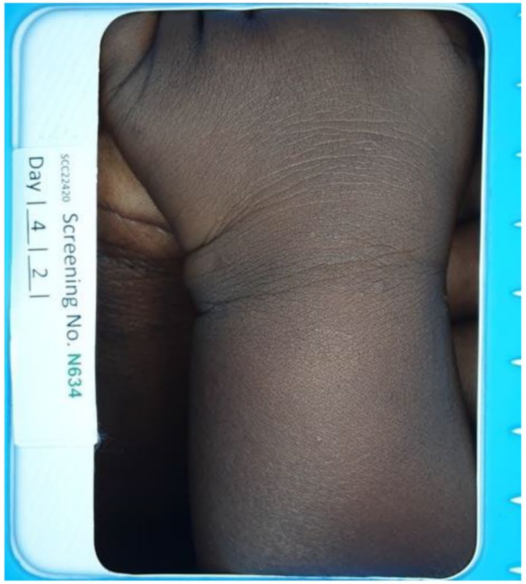

Images were acquired with a Samsung Galaxy Tab A tablet under standardized conditions for composition (5 cm x 8 cm aperture, 12 cm standoff) and lighting (Qiaya Q3). MNP application sites were photographed daily over the day 0 to day 13 solicitation period for the purposes of quality control. The field workers direct assessment of the MNP applications site was used to grade the reactions.

Supplementary Table: Measles and rubella IgG antibody data – adult primary immunogenicity population

		Measles			Rubella		
		**MRV-MNP & placebo SC**	**MRV-SC & placebo MNP**	**Ratio§/ Difference‡**	**MRV-MNP & placebo SC**	**MRV-SC & placebo MNP**	**Ratio§/ Difference‡**
**Baseline**	**Median (Q1-Q3)**	413 (150-780)	228 (188-687)		116 (60-192)	246 (102-410)	
	GMC (95% CI)	373.5 (231.4 to 603.0)	316.3 (202.4 to 494.2)	1.18§ (0.627 to 2.224)	111.7 (84.7 to 147.2)	206.8 (122.7 to 348.5)	0.54§ (0.304 to 0.960)
	Seroprotection (n/N) % (95% CI)	(20/30) 66.7 (48.8 to 80.8)	(10/15) 66.7 (41.7 to 84.8)	0.0‡ (-25.5 to 28.7)	(30/30) 100.0 (88.7 to 100.0)	(15/15) 100.0 (79.6 to 100.0)	0.0‡ (-11.4 to 20.4)
	N	30	15		30	15	
**Visit 4 (Day 42)**	**Median (Q1-Q3)**	2360 (1724-3682)	1175 (854-1651)		230 (182-391)	214 (134-441)	
	GMC (95% CI)	2417.0 (1918.8 to 3044.5)	1143.3 (848.6 to 1540.3)	2.11§ (1.468 to 3.047)	254.1 (203.2 to 317.6)	234.1 (150.1 to 365.1)	1.09§ (0.668 to 1.764)
	GMFR (95% CI)	6.5 (4.3 to 9.8)	3.6 (2.2 to 5.9)	1.79§ (0.956 to 3.354)	2.3 (1.8 to 2.8)	1.1 (0.9 to 1.4)	2.01§ (1.508 to 2.677)
	Seroprotection (n/N) % (95% CI)	(30/30) 100.0 (88.7 to 100.0)	(15/15) 100.0 (79.6 to 100.0)	0.0‡ (-11.4 to 20.4)	(30/30) 100.0 (88.7 to 100.0)	(15/15) 100.0 (79.6 to 100.0)	0.0‡ (-11.4 to 20.4)
	Seroconversion (n/N) % (95% CI)	(10/10) 100.0 (72.3 to 100.0)	(5/5) 100.0 (56.6 to 100.0)	0.0‡ (-27.8 to 43.5)	-	-	-
	N (Baseline seronegative)	10	5		0	0	
	4-Fold Rise (n/N) % (95% CI)	(8/20) 40.0 (21.9 to 61.3)	(2/10) 20.0 (5.7 to 51.0)	20.0‡ (-15.9 to 45.7)	(4/30) 13.3 (5.3 to 29.7)	(0/15) 0.0 (0.0 to 20.4)	13.3‡ (-8.6 to 29.7)
	N (Baseline seropositive)	20	10		30	15	
	Immune Response (n/N) % (95% CI)	(18/30) 60.0 (42.3 to 75.4)	(7/15) 46.7 (24.8 to 69.9)	13.3 (-15.8 to 40.1)	(4/30) 13.3 (5.3 to 29.7)	(0/15) 0. 0 (0.0 to 20.4)	13.3‡ (-8.6 to 29.7)
	Total N	30	15		30	15	
**Visit 5 (Day 180)**	**Median (Q1-Q3)**	1197 (560-1834)	606 (308-904)		189 (130-248)	256 (119-393)	
	GMC (95% CI)	1172.2 (860.5 to 1596.8)	626.9 (453.2 to 867.1)	1.87§ (1.213 to 2.882)	186.8 (144.5 to 241.4)	209.1 (135.8 to 322.0)	0.89§ (0.548 to 1.456)
	Seroprotection (n/N) % (95% CI)	(29/30) 96.7 (83.3 to 99.4)	(14/15) 93.3 (70.2 to 98.8)	3.3‡ (-11.1 to 26.7)	(30/30) 100.0 (88.7 to 100.0)	(15/15) 100.0 (79.6 to 100.0)	0.0‡ (-11.4 to 20.4)
	Total N	30	15		30	15	

Q1 – Lower quartile; Q3 – Upper quartile; CI confidence interval; n – number included in given analysis; GMC - geometric mean antibody concentrations; GMFR - geometric mean fold rise; NA – not applicable; baseline and visit 4 (day 42) analysis are in the primary immunogenicity population; visit 5 (day 180) analysis is in the day 180 secondary immunogenicity population; seroconversion is defined as a change from seronegative at baseline to seropositive at day 42; 4-fold rise is defined as a 4-fold rise in antibody concentrations between baseline and day 42 amongst individuals who were seropositive at baseline; immune response includes all those who were seronegative at baseline and seroconverted on day 42 or who were seropositive at baseline and had a 4-fold rise in antibody concentrations; for measles, seronegative is defined as an antibody concentration of < 200 mIU/mL, seropositive/seroprotection is defined as an antibody concentration of $\geq$ 200 mIU/mL; for rubella, seronegative is defined as an antibody concentration of < 10 IU/mL and seropositive/seroprotection is defined as an antibody concentration of $\geq$ 10 IU/mL; §Ratio [microneedle patch]/[subcutaneous injection]; ‡ Difference [microneedle patch] – [subcutaneous injection]. Estimates are presented with 95% confidence intervals. CIs for the log2 transformed means assume a Student's t-distribution. CIs for seroprotection and seroconversion were calculated using the Wilson score method without continuity correction. CIs for differences between proportions were calculated using the Newcombe method without continuity correction.

Supplementary Table: Measles and rubella IgG antibody data – toddler and infant primary immunogenicity populations

(A) Toddlers		Measles			Rubella		
		MRV-MNP & placebo SC	MRV-SC & placebo MNP	Ratio§/ Difference‡	MRV-MNP & placebo SC	MRV-SC & placebo MNP	Ratio§/ Difference‡
**Baseline**	Median (Q1-Q3)	1083 (628-1600)	1038 (698-1724)		105 (62-142)	97 (44-144)	
	GMC (95% CI)	1111.3 (880.4 to 1402.7)	1024.0 (824.9 to 1271.2)	1.08§ (0.792 to 1.486)	95.9 (79.9 to 115.1)	77.5 (59.6 to 100.8)	1.24§ (0.902 to 1.699)
	Seroprotection (n/N) % (95% CI)	(58/59) 98.3 (91.0 to 99.7)	(59/60) 98.3 (91.1 to 99.7)	-0.0‡ (-7.5 to 7.3)	(58/59) 98.3 (91.0 to 99.7)	(57/60) 95.0 (86.3 to 98.3)	3.3‡ (-4.7 to 12.1)
	N	59	60		59	60	
**Visit 4 (Day 42)**	Median (Q1-Q3)	5896 (3384-7668)	5020 (2731-7918)		171 (124-262)	151 (106-243)	
	GMC (95% CI)	5113.2 (4332.5 to 6034.4)	4887.9 (3981.2 to 6001.1)	1.05§ (0.806 to 1.359)	186.0 (159.7 to 216.6)	162.0 (137.1 to 191.5)	1.15§ (0.918 to 1.436)
	GMFR (95% CI)	4.6 (3.6 to 5.9)	4.8 (3.7 to 6.2)	0.96§ (0.673 to 1.382)	1.9 (1.6 to 2.4)	2.1 (1.7 to 2.6)	0.93§ (0.676 to 1.273)
	Seroprotection (n/N) % (95% CI)	(59/59) 100.0 (93.9 to 100.0)	(60/60) 100.0 (94.0 to 100.0)	0.0‡ (-6.1 to 6.0)	(59/59) 100.0 (93.9 to 100.0)	(60/60) 100.0 (94.0 to 100.0)	0.0‡ (-6.1 to 6.0)
	Seroconversion (n/N) % (95% CI)	(1/1) 100.0 (20.7 to 100.0)	(1/1) 100.0 (20.7 to 100.0)	0.0‡ (-79.4 to 79.4)	(1/1) 100.0 (20.7 to 100.0)	(3/3) 100.0 (43.9 to 100.0)	0.0‡ (-79.4 to 56.2)
	N (Baseline seronegative)	1	1		1	3	
	4-Fold Rise (n/N) % (95% CI)	(35/58) 60.3 (47.49 to 71.91)	(33/59) 55.9 (43.29 to 67.85)	4.4‡ (-13.11 to 21.55)	(7/58) 12.1 (5.97 to 22.88)	(7/57) 12.3 (6.08 to 23.25)	-0.2‡ (-12.76 to 12.25)
	N (Baseline seropositive)	58	59		58	57	
	Immune Response (n/N) % (95% CI)	(36/59) 61.0 (48.3 to 72.4)	(34/60) 56.7 (44.1 to 68.4)	4.3‡ (-13.0 to 21.3)	(8/59) 13.6 (7.0 to 24.5)	(10/60) 16.7 (9.3 to 28.0)	-3.1‡ (-16.2 to 10.1)
	Total N	59	60		59	60	
**Visit 5 (Day 180)**	Median (Q1-Q3)	2356 (1416-4927)	2352 (1682-4103)		149 (103-207)	93 (60-190)	
	GMC (95% CI)	2288.2 (1833.0 to 2856.4)	2551.3 (2155.8 to 3019.3)	0.90§ (0.681 to 1.182)	149.6 (127.6 to 175.3)	100.1 (83.3 to 120.2)	1.49§ (1.176 to 1.899)
	Seroprotection (n/N) % (95% CI)	(56/57) 98.2 (90.7 to 99.7)	(60/60) 100.0 (94.0 to 100.0)	-1.8‡ (-9.3 to 4.4)	(57/57) 100.0 (93.7 to 100.0)	(60/60) 100.0 (93.9 to 100.0)	0.0 ‡ (-6.3 to 6.0)
	Total N	57	60		57	60	

**(A) Infants**		**Measles**			**Rubella**		
		**MRV-MNP & placebo SC**	**MRV-SC & placebo MNP**	**Ratio§/ Difference‡**	**MRV-MNP & placebo SC**	**MRV-SC & placebo MNP**	**Ratio§/ Difference‡**
**Baseline**	Median (Q1-Q3)	13 (10-17)	13 (10-16)		1 (0-1)	1 (1-1)	
	GMC (95% CI)	17.5 (12.9 to 23.9)	16.1 (12.5 to 20.9)	1.09§ (0.730 to 1.620)	1.8 (1.6 to 2.1)	1.8 (1.6 to 1.9)	1.03§ (0.877 to 1.205)
	Seroprotection (n/N) % (95% CI)	(2/59) 3.4 (0.93 to 11.54)	(1/59) 1.7 (0.30 to 9.00)	1.7‡ (-6.01 to 9.97)	(1/59) 1.7 (0.30 to 9.00)	(0/59) 0.0 (0.00 to 6.11)	1.7‡ (-4.58 to 9.00)
	N	59	59		59	59	
**Visit 4 (Day 42)**	Median (Q1-Q3)	2527 (1588-4462)	2679 (1804-4118)		91 (64-173)	151 (82-230)	
	GMC (95% CI)	2633.9 (2203.0 to 3150.0)	2868.3 (2385.0 to 3449.0)	0.92§ (0.713 to 1.184)	104.0 (87.6 to 123.6)	137.1 (114.6 to 163.9)	0.76§ (0.594 to 0.971)
	GMFR (95% CI)	150.2 (114.9 to 196.4)	177.8 (140.5 to 225.0)	0.84§ (0.594 to 1.202)	56.8 (44.9 to 71.9)	77.0 (62.5 to 94.7)	0.74§ (0.541 to 1.007)
	Seroprotection (n/N) % (95% CI)	(59/59) 100.0 (93.9 to 100.0)	(59/59) 100.0 (93.9 to 100.0)	0.0‡ (-6.1 to 6.1)	(59/59) 100.0 (93.9 to 100.0)	(59/59) 100.0 (93.9 to 100.0)	0.0‡ (-6.1 to 6.1)
	Seroconversion (n/N) % (95% CI)	(57/57) 100.0 (93.7 to 100.0)	(58/58) 100.0 (93.8 to 100.0)	0.0‡ (-6.3 to 6.2)	(58/58) 100.0 (93.8 to 100.0)	(59/59) 100.0 (93.9 to 100.0)	0.0‡ (-6.2 to 6.1)
	N (Baseline seronegative)	57	58		58	59	
	4-Fold Rise (n/N) % (95% CI)	(1/2) 50.0 (9.5 to 90.6)	(0/1) 0.0 (0.0 to 79.4)	50.0‡ (-39.1 to 90.6)	(0/1) 0.0 (0.0 to 79.4)	-	-
	N (Baseline seropositive)	2	1		1	0	
	Immune Response (n/N) % (95% CI)	(58/59) 98.3 (91.0 to 99.7)	(58/59) 98.3 (91.0 to 99.7)	0.0‡ (-7.4 to 7.4)	(58/59) 98.3 (91.0 to 99.7)	(59/59) 100.0 (93.9 to 100.0)	-1.7‡ (-9.0 to 4.6)
	Total N	59	59		59	59	
**Visit 5 (Day 180)**	Median (Q1-Q3)	1542 (994-2333)	1667 (884-3018)		129 (93-179)	161 (121-198)	
	GMC (95% CI)	1595.7 (1242.0 to 2051.0)	1725.7 (1369.0 to 2175.0)	0.93§ (0.660 to 1.296)	131.2 (112.0 to 153.8)	158.5 (139.9 to 179.5)	0.83§ (0.678 to 1.011)
	Seroprotection (n/N) % (95% CI)	(57/57) 100.0 (93.7 to 100.0)	(59/59) 100.0 (93.9 to 100.0)	0.0‡ (-6.3 to 6.1)	(57/57) 100.0 (93.7 to 100.0)	(59/59) 100.0 (93.9 to 100.0)	0.0‡ (-6.3 to 6.1)
	Total N	57	59		57	59	

Q1 – Lower quartile; Q3 – Upper quartile; CI confidence interval; n – number included in given analysis; GMC - geometric mean antibody concentrations; GMFR - geometric mean fold rise; NA – not applicable; baseline and visit 4 (day 42) analysis are in the primary immunogenicity population; visit 5 (day 180) analysis is in the day 180 secondary immunogenicity population seroconversion is defined as a change from seronegative at baseline to seropositive at day 42; 4-fold rise is defined as a 4-fold rise in antibody concentrations between baseline and day 42 amongst individuals who were seropositive at baseline; immune response includes all those who were seronegative at baseline and seroconverted on day 42 or who were seropositive at baseline and had a 4-fold rise in antibody concentrations; for measles, seronegative is defined as an antibody concentration of < 200 mIU/mL, seropositive/seroprotection is defined as an antibody concentration of $\geq$ 200 mIU/mL; for rubella, seronegative is defined as an antibody concentration of < 10 IU/mL and seropositive/seroprotection is defined as an antibody concentration of $\geq$ 10 IU/mL; §Ratio [microneedle patch]/[subcutaneous injection]; ‡ Difference [microneedle patch] – [subcutaneous injection]. Estimates are presented with 95% confidence intervals. CIs for the log2 transformed means assume a Student's t-distribution. CIs for seroprotection and seroconversion were calculated using the Wilson score method without continuity correction. CIs for differences between proportions were calculated using the Newcombe method without continuity correction.

Supplementary Figure: Serum neutralizing antibody seroprotection and geometric mean antibody concentrations – adult cohort

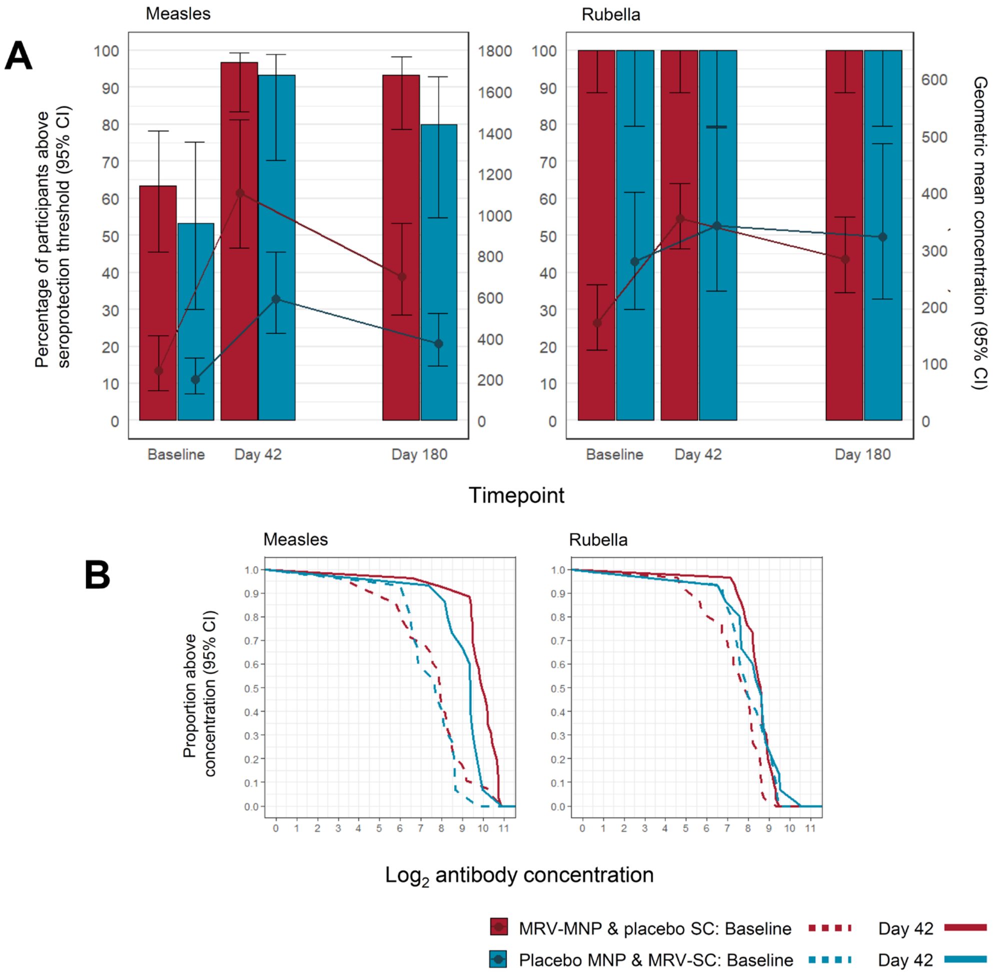

(A) Adult cohort measles and rubella seroprotection rates (solid bars) and 95% confidence intervals; seroprotection rates are defined as the percentage of evaluable participants with an antibody concentration of > 200mIU/mL for measles and > 10IU/mL for rubella. Measles geometric means concentrations are measured in mIU/L. Rubella geometric mean concentrations reported in IU/mL; (B) Adult cohort measles and rubella serum neutralizing antibody baseline and day 42 reverse cumulative distributions curves.

Supplementary references.

1. WHO International Standard, 3rd International Standard for Anti-Measles; NIBSC code: 97/648; Instructions for Use (Version 2.0, Dated 26/02/2008). https://www.nibsc.org/documents/ifu/97-648.pdf (accessed.

2. WHO International Standard; Anti Rubella Immunoglobulin, Human; NIBSC code: RUBI-1-94; Instructions for use; (Version 8.0, Dated 11/03/2019).
